# Simple sequence repeats in zebra finch (*Taeniopygia guttata*) expressed sequence tags: a new resource for evolutionary genetic studies of passerines

**DOI:** 10.1186/1471-2164-8-52

**Published:** 2007-02-14

**Authors:** Jon Slate, Matthew C Hale, Timothy R Birkhead

**Affiliations:** 1 Department of Animal & Plant Sciences, University of Sheffield, Western Bank, Sheffield, S10 2TN, UK

## Abstract

**Background:**

Passerines (perching birds) are widely studied across many biological disciplines including ecology, population biology, neurobiology, behavioural ecology and evolutionary biology. However, understanding the molecular basis of relevant traits is hampered by the paucity of passerine genomics tools. Efforts to address this problem are underway, and the zebra finch (*Taeniopygia guttata*) will be the first passerine to have its genome sequenced. Here we describe a bioinformatic analysis of zebra finch expressed sequence tag (EST) Genbank entries.

**Results:**

A total of 48,862 ESTs were downloaded from GenBank and assembled into contigs, representing an estimated 17,404 unique sequences. The unique sequence set contained 638 simple sequence repeats (SSRs) or microsatellites of length ≥20 bp and purity ≥90% and 144 simple sequence repeats of length ≥30 bp. A chromosomal location for the majority of SSRs was predicted by BLASTing against assembly 2.1 of the chicken genome sequence. The relative exonic location (5' untranslated region, coding region or 3' untranslated region) was predicted for 218 of the SSRs, by BLAST search against the ENSEMBL chicken peptide database. Ten loci were examined for polymorphism in two zebra finch populations and two populations of a distantly related passerine, the house sparrow *Passer domesticus*. Linkage was confirmed for four loci that were predicted to reside on the passerine homologue of chicken chromosome 7.

**Conclusion:**

We show that SSRs are abundant within zebra finch ESTs, and that their genomic location can be predicted from sequence similarity with the assembled chicken genome sequence. We demonstrate that a useful proportion of zebra finch EST-SSRs are likely to be polymorphic, and that they can be used to build a linkage map. Finally, we show that many zebra finch EST-SSRs are likely to be useful in evolutionary genetic studies of other passerines.

## Background

Passerines (perching birds) are one of the most-widely studied taxonomic groups in evolutionary and ecological research [1,2]. They are frequently studied in the wild because they are easy to observe, often breed in nest-boxes or natural cavities, and have short-generation times and large broods. Quantitative genetic studies of passerines have advanced our understanding of natural selection [3,4], sexual selection [5], the effects of inbreeding [6,7], speciation [8,9], the causes of evolutionary stasis [10,11], and the heritability of fitness traits [12,13]. For the latter two areas enormous progress has been made in recent years by quantitative genetic analyses of pedigreed populations [14-19].

While quantitative genetic studies of passerines have set the benchmark in terms of understanding the genetic architecture of fitness-related traits in wild vertebrates, they do suffer from one obvious limitation: they cannot pinpoint the actual loci responsible for adaptive evolution. Indeed, molecular genetic studies of passerines have been somewhat hampered by a lack of genomic resources. In contrast, gene mapping studies of other ecologically relevant vertebrates are becoming increasingly commonplace [20-24]. At present it is not possible to conduct genome-wide mapping or population genomic studies in any passerine species, largely due to insufficient numbers of characterised polymorphic markers such as microsatellites. In fact, only one passerine species, the great reed warbler (*Acrocephalus arundinaceus*), has a genetic linkage map [25], and even then markers cover only ~30% of the genome.

Fortunately, this situation is beginning to be addressed. There are currently ~900 passerine microsatellite markers deposited in GenBank, although the majority are only informative in the species in which they were originally isolated, or closely related species [26]. The recently assembled draft genome of the red junglefowl *Gallus gallus *[27], the progenitor of the domestic chicken, will also facilitate further molecular studies in passerines. Despite a divergence date of ~100 million years ago, Galliformes (the order that includes the chicken) and Passeriformes show highly conserved karyotypes [28-31], which means the chicken genome assembly is a useful comparative resource for molecular studies of passerines. For example, the map location of ~200 passerine microsatellites was recently predicted by using BLAST to identify regions of high sequence similarity between passerine microsatellite flanking regions and the chicken genome assembly[28]. Regions of high homology may prove useful in designing primers to amplify a locus in as diverse an array of passerine species as possible. However, this approach has yet to be successfully attempted on a large scale and it is unclear to what extent the repeat motif is conserved across divergent families.

The prospects for molecular evolution, gene mapping, comparative genomics and population genomic studies in passerines are greatly improved by ongoing efforts to sequence the zebra finch (*Taeniopygia guttata*) genome [32]. Genomics resources for the zebra finch include >50,000 expressed sequence tags (ESTs), mostly generated as part of the Songbird Neurogenomics Initiative [33]. The aim of this paper is to demonstrate that microsatellite loci within zebra finch ESTs are a useful resource for population and comparative genomics studies in the zebra finch and in other passerine species.

In recent years it has become apparent that sequence databases can be used as a tool to rapidly identify microsatellite loci, thereby avoding time-consuming library screening. It is now known that microsatellites are present within most eukaryote genomes, principally in intergenic regions, but also within introns and exons [34]. *In silico *detection [35,36] and validation [35,37] of exonic microsatellites from EST databases (hereafter EST-simple sequence repeats or EST-SSRs) has been achieved in economically important plants, and to a lesser extent in vertebrates [38,39]. In cereals it is estimated that 4–6% of genes contain EST-SSRs of at least 20 bp length [36]. There is some evidence that EST-SSRs lack variation compared to those in introns or intergenic regions [40], but even the lowest estimates suggest that at least 25% of EST-SSRs are polymorphic. EST-SSRs offer two advantages over intergenic microsatellites. First, because they are exonic, their flanking regions will often be functionally-constrained. Therefore, it is likely that PCR primers for EST-SSRs can be used to genotype loci in related species to the source species. Second, because they are exonic, they are more likely than intergenic microsatellites to be in strong linkage disequilibrium with functionally important sites. This makes them well suited to population genomics or gene mapping applications that hope to map genes of economic or adaptive significance.

In this paper we describe an analysis of zebra finch EST accessions deposited in GenBank. Our objectives were to: (i) Identify and describe EST-SSRs in the zebra finch. (ii) Establish whether these loci are likely to be polymorphic within the zebra finch, and a distantly related passerine, the house sparrow (*Passer domesticus*). (iii) Predict the map location on the *Gallus gallus *genome of the homologue of each EST-SSR. Because the passerine and *Gallus *genomes show a high degree of synteny [28-31,41], such an analysis will help predict the location of each EST-SSR in the zebra finch genome. (iv) Predict whether each EST-SSR is within the coding region, the 5' untranslated region or the 3' untranslated region of the exon in which it resides. This information will be useful in predicting which loci are most likely to be polymorphic. Coding region microsatellites are likely to be under the strongest functional constraint, and therefore be the least variable. This represents the first study of its kind in passerines, a diverse and scientifically important taxon, that is widely studied in evolutionary biology and ecological research.

## Results

### Summary of identified EST-SSRs

A total of 48,862 zebra finch ESTs were analysed. 9,845 were unique (hereafter termed singletons) and the remainder were components of a total of 7,559 contigs. Thus, we estimate that 17,404 unique sequences were present in the database. Note that the chicken genome database lists ~30,000 unigenes. This suggests that around 55% of genes or expressed pseudogenes are represented in the zebra finch EST dataset.

1,278 repeats were identified, from a total of 1209 different ESTs (i.e. some ESTs contained more than one repeat). After checking for redundancy, the EST-SSRs were attributed to 426 singleton ESTs and 212 EST-contigs; i.e. 638 unique sequences contained a repeat that was at least 20 bp long (Table 1). One hundred and forty-four of the repeats were at least 30 bp long. Therefore, we estimate that 3.67% (638/17,404) of loci contain a microsatellite of length ≥20 bp and 0.83% of loci contain a microsatellite of at least 30 bp length. These estimates are conservative, as most ESTs or assembled contigs are not full gene transcripts. Di- and tri-nucleotides were more prevalent than tetra or penta-nucleotides. The most common microsatellite motif was the dinucleotide AT; 110 different AT repeats of length ≥20 bp and purity ≥90% were identified. Summaries of each repeat and motif type are provided in Table 1 and Table 2. Detailed descriptions of each EST-SSR are provided in the Additional File 1.

### In silico mapping of EST-SSRs

Of the 638 EST-SSR loci, in total 434 (68%) were assigned a predicted map position with an E value of 1e^-10 ^or better. Orthologues of zebra finch EST-SSR loci were assigned to all assembled chicken chromosomes [see Additional File 3], except the microchromosomes Gga32 and GgaE64. Among mapped EST-SSRs 130 were dinucleotides, 148 were trinucleotides, 49 were tetranucleotides and 107 were pentanucleotides. Assignment success rates did not vary between the different repeat types. The mean sequence similarity between a zebra finch EST and its matching chicken orthologue was 91.6%.

**Figure 1:**
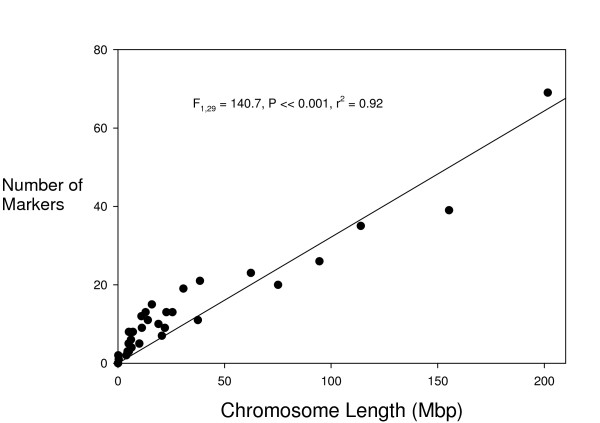
**Regression of number of *in silico *mapped EST-SSRs on chicken chromosome length**. Chicken chromosomes are generally numbered largest first, so chromosome 1 is the data point in the top right corner. Chromosome length is a good predictor of the number of mapped EST-SSR loci. However, microchromosomes are relatively EST-SSR abundant relative to macrochromosomes (chromsomes 1–5 and Z).

*In silico *mapped EST-SSRs were approximately evenly distributed across the chicken genome. A general linear model was fitted to formally examine marker distribution, where the response varaible was the number of markers per chromosome and the predictors were chromosome length and chromosome category (chromosomes 1–5 and Z were regarded as macrochromosomes and all others were regarded as microchromosomes). Chromosome length was a good predictor of the number of EST-SSRs that were mapped to each chromosome (F_1,29 _= 140.7, P << 0.001) and explained ~92% of the variance. However, it was also evident that the density of EST-SSR loci was greater on microchromosomes than macrochromosomes; chromosome cateogry explained an additional 1.4% of the variance (F_1,29 _= 5.99, P = 0.021), although marker density was also relatively high on the largest chromosome, *Gga*1 (Figure 1).

### Exonic location of EST-SSRs

Two hundred and eighteen EST-SSR loci showed significant homology to known or predicted genes in the Ensembl chicken peptide database [see Additional File 1]. Seventeen were dinucleotides, 114 were trinucleotides, 21 were tetranucleotides and 66 were pentanucleotides. It was relatively unusual for the repeat motif to reside within the coding region of an exon (38/218 cases = 17.4%), although frequencies ranged from 0% (dinucleotides) to 30% (trinucleotides). Only four of the coding region EST-SSRs were not trinucleotides.

### Comparison with existing passerine microsatellites

Six EST-SSR loci showed high sequence similarity to sequence flanking published passerine microsatellites (Table 3); i.e. they were homologues of previously known loci. All other EST-SSRs were not previously described.

### Polymorphism of EST-SSRs – *in silico *analysis of contigs

All of the EST contigs containing di, tri- and penta- nucleotide loci that had been sequenced three or more times were examined for repeat length polymorphism (very few tetranucleotides were sequenced more than twice). Eleven of the twenty five dinucleotides were polymorphic, as were 10/26 trinucleotides and 6/13 pentanucleotides, giving a total of 27/64 (42%) polymorphic markers. When the analysis was restricted to contigs with four or more overlapping sequences, the proportion of polymorphic loci was greater (21/41 = 51%). The loci included in these analysis are unbiased with respect to repeat length or purity.

### Polymorphism of EST-SSRs – laboratory data

Eight of the ten (80%) primer pairs produced a polymorphic product in both populations of zebra finch (Table 4). The number of alleles per locus ranged from 2–9, and the observed heterozygosity ranged from 0.25–0.91. Genetic diversity was broadly similar in the two populations. Not surprisingly, the EST-SSR primers had lower amplification success in house sparrow populations, although 7/10 and 6/10 amplified products of the expected size in the Lundy and Aldra populations, respectively. All seven loci in the Lundy population were polymorphic (number of alleles 2–6, observed heterozygosity 0.28–0.64) and four of the loci were polymorphic in the Aldra birds (2–5 alleles, heterozygosity 0.43–0.66). All markers were in Hardy-Weinberg Equilibrium (HWE). One EST-SSR (Contig 206) was predicted to map to the Z chromosome; in support of this prediction all genotyped females (the hemizygous sex) had just one allele. When only male genotypes were considered, the marker was in HWE.

### Linkage mapping of EST-SSRs

Twopoint linkage analysis conducted on the Sheffield population of zebra finches produced highly significant LOD scores between three of the four EST-SSRs that were *in silico *predicted to be linked. Pairwise LOD scores and Kosambi map distances were as follows: DV952125 and DV982809 (LOD = 7.80, distance = 4 cM); DV955012 and DV952125 (LOD = 13.25, distance = 9 cM); DV955012 and DV952809 (LOD = 6.28, distance = 6 cM). The fourth locus, CK304956, provided weaker, but nonetheless some, evidence of linkage (twopoint LOD to DV952125 = 2.39, distance = 14 cM; twopoint LOD to DV955012 = 2.09, distance = 16 cM). The marker order predicted from the chicken genome sequence was CK304956-DV952125-DV952809-DV955012, which was also the marker order that produced the highest likelihood in the mapping population.

## Discussion

### Properties of zebra finch EST-SSRs

SSRs appear to be relatively abundant within zebra finch ESTs. 3.7% of unique sequences contain an SSR greater than 20 bp long and almost 1% of unique sequences contain an SSR greater than 30 bp long. These values are comparable to studies of other species, most of which have been conducted in cereals or other plants [36,42,43]. These are likely to be conservative estimates of the number of SSRs per gene as many ESTs or assembled contigs do not span the entire length of the gene transcript. Estimates of the proportion of ESTs that contain EST-SSRs are generally not available for other vertebrates. However, our unpublished data indicate that approximately 3.8% of chicken unigenes contain EST-SSRs while in mammals the proportion ranges from ~2.0% in sheep to ~15.6% in mouse. Note that inter-genic microsatellites are thought to be much rarer in avian genomes than mammalian genomes [44] but there is little indication that a similar pattern holds for EST-SSRs.

Among EST-SSRs of ≥20 bp length, trinucleotides were the most abundant type of repeat motif, followed by dinucleotides. Among EST-SSRs ≥30 bp, the two repeat types were equally abundant (each ~35% of all SSRs). The proportion of dinucleotides appears to be similar in the zebra finch as in other species for which comparable data are available, e.g. [36,45]. The observation that dinucleotides are relatively more frequent among long EST-SSRs is also consistent with previous studies [36].

The most common motif among EST-SSRs ≥20 bp or ≥30 bp was the dinucleotide AT. Similar observations have been made in rice [36] and several species of pine [35], but this pattern is by no means consistent across species [36,43]. The relative frequency of different trinucleotide motifs was dependent on SSR length. Among SSRs ≥20 bp AGG was the most common (12% of all EST-SSRs), but among the SSRs ≥30 bp AAT was the most common (13%). In other species there is no clear consensus to which trinucleotides are most frequent [35,36,46].

Approximately 1/3 of EST-SSR loci could be assigned to known genes in the Ensembl chicken peptide database. It was possible to predict the within-gene location of these EST-SSRs, which revealed clear differences between the repeat types. Trinucleotides were more often located in coding sequence (CDS) of genes (29.8% of cases) than other repeat types (0%, 10% and 3% for dinucleotides, tetranucleotides and pentanucleotides respectively). This observation is expected as a loss or gain of repeat unit in a trinucleotide will not result in a frameshift mutation. CDS trinucleotide repeats are of particular interest, as in other organisms a number of pathologies and behaviours are associated with triple repeat expansions [47-51]. There were two tetranucleotides and two pentanucleotides that were identified within the CDS of genes. However, all four loci were relatively short (4–5 repeat units long) and two of them were interrupted. Therefore, these loci may have relatively low mutation rates, minimising the probability of frameshifts arising. Among non-CDS EST-SSRs trinucleotides and pentanucleotides were more likely to be in the 5' UTR than the 3'UTR, while the opposite was true for dinucleotides. A similar pattern is observed in pine species [35], although there are relatively fewer EST-SSRs in the CDS of zebra finch, regardless of repeat type. In practical terms this is useful as non-CDS SSRs are the most likely to be polymorphic (see below).

Chromosomal location of two-thirds of EST-SSR loci was predicted by *in silico *mapping to the chicken genome. It should be noted that these predicted chromosomal locations can only be confirmed when the zebra finch genome sequence is assembled or a linkage map constructed. Given that synteny is highly conserved between the chicken and passerine genomes [28,30] it is likely that loci assigned to a particular chicken chromosome will prove to be linked in the zebra finch. Therefore, EST-SSRs appear to be dispersed approximately evenly across the zebra finch genome, although they are probably at a marginally higher density on microchromosomes than macrochromosomes. This observation is consistent with the reports that gene density in chickens is greatest on the microchromosomes [27]. Once these EST-SSRs are assigned a map position in the zebra finch (and other species) they will provide insight into the extent of chromosomal rearrangements between different avian lineages. Note that the four linked loci that we mapped appeared to be in the same order as their linked homologs on chicken chromosome 7.

### Applications of EST-SSRs

The SSRs identified in this study represent a useful resource for evolutionary genetic studies of birds. Most obviously, they can be used to build a zebra finch linkage map, possibly acting as framework loci, used in tandem with SNPs typed at a higher density. We have demonstrated that linkage map construction should be relatively straightforward, and will provide a useful complement to ongoing efforts to construct a physical map. Evolutionary quantitative genetic studies of zebra finches have estimated the heritability of traits such as stress response [52], sperm morphology [19], body condition [53], bill colour [54] and digit ratio [55]. The availability of a linkage map would facilitate the next stage of genetic studies (i.e. mapping the loci that determine additive genetic variance) of an important model organism in evolutionary and ecological research. A linkage map would also represent a useful tool to aid assembly of the zebra finch genome once shotgun sequencing is complete, because it can help identify which contigs reside on particular chromosomes. Assembly of the chicken genome sequence was partially reliant on the consensus chicken linkage map [56].

Use of the EST-SSRs described here need not be restricted to studies of the source species. Previous studies have estimated that less than 10% of passerine microsatellites are polymorphic in species from different taxonomic families [26,57,58]. Therefore, although >900 microsatellites have been isolated in passerines, they were derived from ~75 different species, and the majority are not informative in any one species. The location of these microsatellites relative to genes was unknown, although the majority were probably intergenic. There are several reasons to suspect that EST-SSRs will be much more widely applicable across species than intergenic microsatellites.

First, because EST-SSRs are located within exons they are likely to be under greater functional constraint than intergenic microsatellites. Therefore, sequence that flanks the repeat motif of an EST-SSR is expected to diverge at a slower rate than is commonly observed with intergenic markers. This expectation is demonstrable with our data. Using the BLASTn default settings 68% of EST-SSRs showed significant (E < 1e^-10^) homology to the chicken genome, with an average sequence similarity of 91.6%. This figure compares favourably with a study of passerine intergenic microsatellites [28], where just 14.0% of markers showed sequence similarity to chicken at E < 1e^-10 ^under the same settings.

Secondly, an encouraging proportion of the limited number of zebra finch EST-SSRs that we tested were polymorphic in another passerine species, the house sparrow. Estimating divergence times between passerine species is not straightforward, but zebra finches and house sparrows probably diverged between 20 MYA [59] and 45MYA [60] and are in completely different families (Estrildidae and Passeridae). Therefore, polymorphic zebra finch EST-SSRs appear to be conserved across passerine families.

A third piece of support for the widespread applicability of zebra finch EST-SSRs is provided by the small proportion (6 out of 638) of loci identified in this study that have also been isolated in other passerines. Two of these markers Ase49 (cloned in the Seychelles warbler *Acrocephalus sechellensis *and homologous to contig 26) and MSLP4 (isolated in the Japanese Marsh Warbler *Locustella pryeri *and homologous to DV951916) have been examined with respect to cross-species amplification success rate [61,62]. Both loci are polymorphic in species of different sub-families to the source species, and in fact no other markers cloned in these species have greater cross-species amplification success.

Although, there is substantial evidence that the zebra finch EST-SSRs are conserved across other passerine species, there use in many population genetic studies will be limited unless they are polymorphic (although monomorphic loci may still be useful in molecular evolution studies). Data presented here and elsewhere indicate that a reasonably large proportion of EST-SSRs will be polymorphic in other passerines. Among loci that produced a PCR product 8/8 (100%) were polymorphic in the source species (the zebra finch) and 6/7 (86%) were polymorphic in a distantly related passerine, the house sparrow.

*In silico *analysis of contigs with three or more overlapping sequences indicated that greater than 40% of loci were polymorphic within the zebra finch. This figure is likely to be an underestimate of the proportion of polymorphic markers for two reasons. First, the majority of loci were represented by only 3 or 4 sequences, which means polymorphism will be undetected at some variable loci. This point is illustrated by the fact that an analysis restricted to loci represented by four or more sequences, resulted in a higher estimate of 51%, while a similar analysis of uninterrupted repeats yielded an estimate of 60% [see Additional File 2]. Second, because many of the ESTs come from the same libraries, it is inevitable that some contigs will include multiple sequences from the same individual and will not be independent – thereby making it impossible to detect polymorphism. More generally, there is already good support that ESTs-SSRs are often polymorphic within both the source species and other species [37-40,42].

There are several strategies that could be employed to ensure that future laboratory efforts focus on zebra finch EST-SSRs that are variable in the source species and in other species.

The first way in which polymorphic EST-SSRs could be identified is to concentrate laboratory efforts on dinucleotide repeats. Previous studies have shown dinucleotides to be more polymorphic than longer repeat types [38,46,63,64], although an analysis of passerine intergenic microsatellites did not support this observation [26]. Secondly, it is likely that EST-SSRs within non-coding regions are more variable than those found in coding regions, as they are less likely to be under functional constraint. This prediction does have empirical support from studies of rice [46] and bread wheat [45]. Note that among EST-SSRs identified in this study, dinucleotides were the least likely to be in the coding region, which again supports the maximisation of laboratory efforts on dinucleotides. A third way to enhance the proportion of EST-SSRs that are polymorphic is to focus efforts towards the longest and purest repeats. Among passerine intergenic microsatellites there is a significant positive relationship between repeat length and the probability of being polymorphic [26]. Similarly, there is a positive relationship between repeat length and heterozygosity in a variety of taxa, including birds [65]. This pattern seems to hold for EST-SSRs [38,43]. There is also empirical support for the idea that uninterrupted (ie pure) repeats are more variable than those with interruptions [66,67]. Finally, polymorphism can be detected *in silico *within overlapping EST-SSR sequences. In summary, the 51 dinucleotide EST-SSRs that are ≥30 bp long, and the putatively polymorphic loci reported in Additional File 2 are probably the most likely to be variable in zebra finches and other passerine species.

## Conclusion

An analysis of zebra finch ESTs identified greater than six hundred previously undescribed microsatellites (EST-SSRs). *In silico *mapping of these EST-SSRs to the assembled chicken genome sequence indicated that their homologues are approximately evenly dispersed throughout the chicken genome. Given that Galliformes and Passeriformes share a highly conserved karyotype, these EST-SSRs are expected to also be evenly spread throughout the genomes of the zebra finch and other passerines. The majority of these microsatellites are not found within exonic coding regions, suggesting that they need not be functionally constrained, and therefore may be polymorphic. This prediction appears to be confirmed from a screen of a subset of markers in both the source species (the zebra finch) and a distantly related species (the house sparrow), as well as *in silico *detection of repeat length polymorphism. We have also demonstrated that these EST-SSRs can be used to construct a linkage map of the zebra finch, by genotyping three generations of a pedigreed captive population. Further marker development from these EST-SSRs will complement ongoing evolutionary genetics research in birds, including comparative genomics, gene mapping and population genomic studies of both captive and wild populations.

## Methods

### Estimating the number of unique sequences

All available zebra finch ESTs was downloaded from GenBank. The number of non-redundant gene clusters represented in this sample was estimated by building contigs from all sequences, using the version of the CAP3 program [68], available on the rosaecea genome database site [69].

### EST-SSR identification

All ESTs were checked for repeats using a modified version of the Sputnik program [70], using the settings -s 10 (minimum score = 10) and -L 20 (minimum length = 20 bp). Because there may be redundancy among the identified repeats, we then built contigs from just those ESTs containing SSRs, using the CAP3 contig assembly program implemented on a web browser [71]. In all subsequent analyses we ignored repeats of < 90% purity.

The search strategy outlined above includes interrupted repeats, and is consistent with search parameters used in similar studies of other taxa [36]. Because some researchers may be principally interested in uninterrupted repeats we performed a similar search that restricted the output to sequences with at least five consecutive uninterrupted repeat units. This dataset is not the main focus of the paper, but is reported in Additional File 2.

### In silico detection of polymorphism

Because redundant ESTs were clustered into contigs, we were able to compare the number of repeat units in overlapping sequences and identify polymorphic SSRs. We examined all contigs that were assembled from three or more overlappnig sequences and estimated the proportion that were polymorphic.

### In silico mapping of EST-SSRs to the chicken genome

The predicted location of the orthologue of each EST-SSRs was predicted by a similarity search against the chicken genome. Because synteny is highly conserved in avians, loci that are predicted to map to the same chicken chromosome are also likely to be linked in the zebra finch, and in any other passerine species in which they are informative. Therefore assignments of each EST-SSR to a chicken chromosome will enable researchers to design sets of markers of linked (or unlinked) markers prior to the construction of zebra finch physical or linkage maps. When the zebra finch genome is sequenced and assembled it will also be possible to map each locus in the zebra finch, thereby enabling comparison in marker order between the chicken and zebra finch genomes.

Chromosomal location of EST-SSRs was predicted using the BlastN program [72] implemented locally on a workstation. The chicken genome sequence (version WASHUC2.1, released in June 2006) was downloaded from the Genome Sequencing Center, Washington University School of Medicine chicken genome site [73], and all sequences were placed in a single FASTA-formatted text file. Searchs were performed under the default settings, except that the Expectation Value (E) was decreased from 10 to the more stringent setting of 1e^-5^. A locus was assigned to a location in the chicken genome if it provided a unique match (hit) at 1e^-10 ^or lower. If a locus did not provide a single unique hit but provided multiple matches at 1e^-10 ^then it was unassigned unless the best hit had an E value at least 10 decimal places lower than the next best hit. Repeat motifs were masked using the DUST filter (the default BLASTn filter for masking repetitive or low complexity sequence), otherwise the repeat motif of the EST-SSR would have spuriously matched many microsatellites within the chicken genome. These settings were identical to those used in a study that *in silico *mapped intergenic passerine microsatellites to the chicken genome [28], enabling comparison between the EST-SSRs and intergenic markers.

Any markers that were assigned to the W chromosome of chicken between nucleotides 195,832 and 4,895,451 were not placed on the map because the assembly of the W chromosome was built on the basis of assumed W-specific repeats that were later found to occur elsewhere in the chicken genome (details available via the Ensembl Chicken Genome Browser [74])

### Within-exon location of EST-SSRs

The relative position of each repeat within a gene was determined following assignment of EST-SSR loci to functional genes. Each EST-SSR locus was compared against the Ensembl *Gallus gallus *super-set of translated known or novel genes [75] using the BLASTx program, again implemented on a Windows XP workstation. Comparison of the position and orientation of the coding region to the region that showed homology to the zebra finch EST-SSR meant that the relative location of each SSR could be assigned to one of the following categories: coding sequence (CDS), 5' untranslated region (5' UTR) or 3' untranslated region (3' UTR).

### Comparison with existing passerine microsatellites

We also determined whether any EST-SSRs matched previously published passerine microsatellites. Using the search terms 'passeriformes [orgn] AND microsatellite' we identified >900 sequences from Genbank. Where orthologues of a particular locus were known to have been sequenced in multiple species we retained only the original locus, to avoid redundancy in the database. Any sequences that were clearly not microsatellite loci were also excluded. In total 876 sequences were retained. Sequence similarity between EST-SSRs and the 876 microsatellite sequences was determined using BLASTn, as described above.

### Laboratory testing of a subset of EST-SSRs in two passerine species

Primers were developed to amplify ten EST-SSRs with a repeat purity in excess of 90%. Nine dinucleotide and 1 tetranucleotide loci were investigated. Tested loci were not significantly longer or less interrupted than untested loci, i.e. they should be unbiased with respect to observed levels of polymorphism. Primers were designed with the PRIMER3 software [76] and selected to be in regions with high sequence similarity to the chicken homologue. Four of the dinucleotides and the tetranucleotide were predicted to map to neighbouring regions of chicken chromosome 7. The primers were tested in two populations of zebra finch: one aviary population housed at the University of Sheffield (described in [19]), and a wild population from close to Broken Hill, New South Wales, Australia (31°57'S, 141°26'E). The provenance of the aviary population is not well known, as no live birds have been imported to the UK since the 1960s. However, the population is known to have been founded from multiple sources within the UK, and all birds are homozygous for the wild type genotype. In order to examine cross-species utility the primers were also tested in two wild populations of house sparrow (*Passer domesticus*) from the Isle of Lundy, Britain (51°10'N, 4°39'W) [5], and from Aldra Island, Norway (66°24'N, 13°5'E) [77]. Each primer pair was tested in 24 individuals from each of the populations studied.

DNA was extracted using standard ammonium acetate procedures from blood stored in 95% ethanol. PCR amplification was performed in 10 μl reactions consisting of 1 μl of template DNA, plus 2.0 mM MgCl_2_, 0.8 Mm dNTPs, 1 μm of each primer, 1 × NH_4 _reaction buffer and 0.5 units of Taq (Bioline). Each reaction was amplified using the same PCR protocol of 3 min initial denaturation at 95°C, then 35 cycles of 30 seconds at 95°C, 30 seconds at 58°C and one minute at 72°C. PCRs were terminated with a final 5 minute extension phase at 72°C. PCR reaction mixtures were initially checked for successful amplification on a 1.5 % agarose gel stained with ethidium bromide, and viewed under UV light. Successful amplification products were then run on an ABI3730 capillary sequencer. Allele calling was performed with the GENEMAPPER (v 3.7) software. The GenAlEx Excel macro [78] was used to measure diversity indices and to test for deviations from Hardy-Weinberg equilibrium.

### Linkage mapping of EST-SSRs in a captive zebra finch population

All four dinucleotide EST-SSRs predicted to map to chicken chromosome 7 produced a polymorphic product in the Sheffield zebra finch population. The markers were subsequently typed and analysed in a mapping panel of 350 pedigreed individuals spanning three generations. Pedigree inconsistencies and genotyping errors were checked and resolved with PEDCHECK [79]. Linkage analysis was performed with CRIMAP [80]. The TWOPOINT command was used to test for linkage between each pair of markers, with a LOD score of 3.0 regarded as evidence for linkage. The predicted marker order from the chicken genome assembly was initially chosen as the most likely order, and alternative orders were tested using the FLIPS option.

## Authors' contributions

JS planned and performed the bioinformatic analyses. MCH conducted the laboratory work. TRB established and guided the maintenance of the mapping population. All authors were involved in writing the manuscript. All authors read and approved the final manuscript.

## Supplementary Material

Additional File 1 EST-SSRs identified from zebra finch ESTs deposited in GenBank. Details of all identified EST-SSRs, including GenBank accession numbers, predicted chromosomal locations, motif type and length, and within-exonic location. Click here for file

Additional File 2 Sequences containing uninterrupted microsatellties of at least 5 repeats long. Details of all identified EST-SSRs with a minimum of five consecutive perfect repeat units. GenBank Accession numbers, and motif type and length are reported. Putatively polymorphic loci in zebra finch are indicated. Click here for file

Additional File 3 Predicted location of orthologs of zebra finch EST-SSRs in the chicken genome. Map indicating location of orthologs on zebra finch EST-SSRs on Assembly 2.1 of the chicken genome. Click here for file

## Figures and Tables

**Figure 2:**
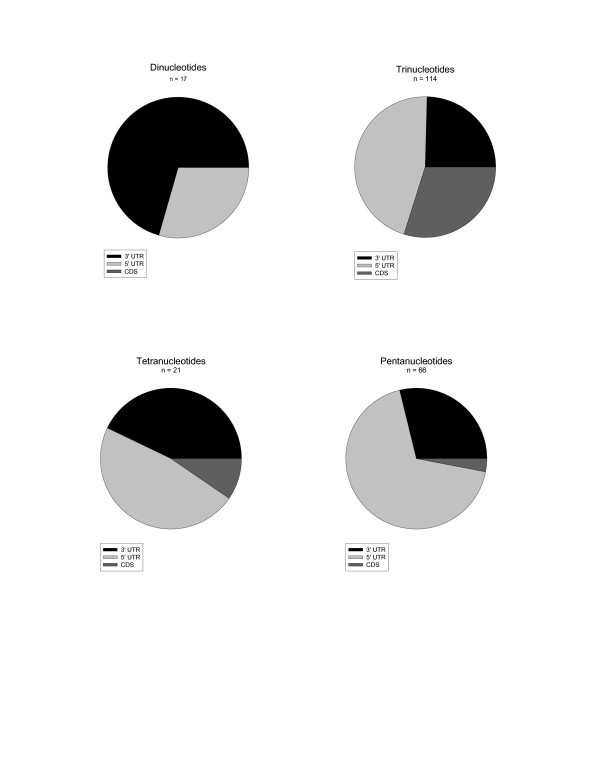
**Exonic distribution of different motifs**. The majority of EST-SSRs are within the 3'UTR or 5'UTR, although coding sequence (CDS) trinucleotide EST-SSRs are relatively common.

**Table 1:** Relative abundance of SSR repeats within zebra finch ESTs.

SSR type	≥ 20 bp		≥ 30 bp	
	N	Freq	N	Freq

di-	173 (58)	0.27	51 (14)	0.35
tri-	224 (115)	0.35	50 (16)	0.35
tetra-	86 (60)	0.13	9 (2)	0.06
penta-	155 (122)	0.24	34 (15)	0.24

Total	638 (355)		144 (47)	

Prop.	3.7%		0.8%	

The number of SSRs of length greater than or equal to 20 bp and 30 bp are reported. The number in parentheses is the number of perfect, i.e. uninterrupted, repeats. Repeats with less than 90% purity are unreported. Prop. is the proportion of all unique sequences that contained an SSR of at least 20 bp or 30 bp.

**Table 2:** The most abundant motifs among EST-SSRs ≥ 20 bp and ≥ 30 bp

SSR ≥ 20 bp			SSR ≥ 30 bp		
Motif	N	Freq	Motif	N	Freq

AT	110	0.17	AT	34	0.24
AGG	74	0.12	AAT	18	0.13
CCG	56	0.08	AGG	12	0.08
AC	43	0.07	AC	11	0.08
AAAAC	37	0.06	AGC	10	0.07
AGC	35	0.05	CCG	8	0.06
AAT	28	0.04	AGCCG	7	0.05
CCCGG	26	0.04	AG	6	0.04
AAAC	21	0.03	ACGGC	5	0.03
AG	19	0.03	AAAAC	4	0.03
			AAAAT	4	0.03

Total	449	0.70	Total	119	0.83

Frequency is the proportion of all EST-SSRs represented by each motif.

**Table 3:** EST-SSR loci with significant homology to previously described passerine markers

EST-SSR	Passerine marker	GenBank Accession	% similarity	E value
DV951916	*Locustella pryeri *MSLP4	AB031376	91	5e^-44^
DV958593	*Sayornis Phoebe *SAP47	AY823673	79	1e^-112^
Contig 26	*Acrocephalus sechellensis *Ase49	AJ276778	93	9e^-52^
Contig 35	*Pomatostomus temporalis *Pte24	DQ234870	96	1e^-127^
Contig 125	*Diglossa cyanea *DcyAAGG142	DQ008009	82	1e^-130^
Contig 131	*Dendroica kirtlandii *DkiD126	AY769692	79	3e^-30^

**Table 4:** Amplification success of ten EST-SSR markers in two zebra finch and two house sparrow populations.

GI number	Primers	Expected size	Zebra finch					House sparrow				
			Size range	Sheffield		Australia		Size range	Lundy		Aldra	
			
				k	H_o_	k	H_o_		k	H_o_	k	H_o_
			
CK304956	F-GGAATGCGTGGACCTTCTGGT R-TAGATTCTTTCATCAAGTTTCTGCCC	184	184–187	3	0.444	Not tested	Not Tested	181–185	4	0.60	4	0.76
DV952809 (contig23)	F-CACATCAAAGGATGTGGTGG R-CAATGTGAATTGCAGGGTCA	192	189–191	2	0.25	2	0.38	195–197	2	0.44	1	0.00
DV952125	F-TGGATTACCTGTCTGAAAGACC R-TTCACTGTCTAGTCCAACCCTGT	206	202–210	5	0.86	5	0.5	151–171	2	0.28	2	0.43
DV955012	F-TCTCAGCATCAGTGTGTTCTGTAC R-TTTGCTTGGGATTGAAGTGA	289	282–307	6	0.91	3	0.52	296–298	2	0.35	1	0.00
CK313552	F-GGAGTTGGAGCGCAAGAAGC R-CGTCTTCCAAATCCGTGAGT	235	-	-	-	-	-	-	-	-	-	-
DV946651	F-CACAGAAAAGTGAGTGCATTCC R-TGGGAAAACATCTTTACCATCA	249	243–262	3	0.35	5	0.89	287–308	5	0.64	5	0.60
CK307697 (contig35)	F-CGAGTAGCGTATTTGTAGCGA R-AGGAGCGGTGATTGTTCAGT	180	170–183	6	0.79	4	0.62	171–181	6	0.55	4	0.66
DV948303	F-CTTCCTGCTATAAGGCACAGG R-AAGTGATCACATTTATTTGAATAT	118	107–117	3	0.38	6	0.74	96–105	3	0.50	-	-
CK312585 (contig 73)	F-ATTCTCAGGTCAATCGCTCACACT R-CAATGCAACAATCTACTCAACACC	434	-	-	-	-	-	-	-	-	-	-
DV959049 (contig 206)	F-GGGGACGTTTTATCTGTTACC R-GCAGTGCCCAAGTTCAGAGT	244	207–230	4	0.47	9	0.59	-	-	-	-	-

GI number is the Genbank identification number of each EST sequence. k = number of alleles, H_o _= observed heterozygosity. 24 birds were typed from each population. All were in Hardy-Weinberg Equilibrium. All are dinucleotides except CK313552 which is a tetranucleotide. Further details on these EST-SSRs (including repeat motif and length, and predicted chromosomal assignments) are available in Additional Files 1 and 2.
