# Supplementary material for: Simple sequence repeats in zebra finch (Taeniopygia guttata) expressed sequence tags: a new resource for evolutionary genetic studies of passerines
Source: BMC Genomics. 2007 Feb 14;8:52. doi: 10.1186/1471-2164-8-52 (PMC1804275; doi:10.1186/1471-2164-8-52)
Supplement: Additional File 1 — EST-SSRs identified from zebra finch ESTs deposited in GenBank. Details of all identified EST-SSRs, including GenBank accession numbers, predicted chromosomal locations, motif type and length, and within-exonic location. [file 1471-2164-8-52-S1.doc]

**Additional File 1**

**EST-SSRs identified from zebra finch ESTs deposited in GenBank.**

Each locus is named according to its database accession name (i.e. on the GenBank nucleotide database), unless a contig was built from several ESTs, in which case it is named Contig *n*. For each contig the accession number of the longest EST within that contig is also provided. Repeat length refers to the length in nucleotides of the repeat motif. % purity is the percentage of the repeat motif that is uninterrupted, and the maximum number of uninterrupted repeats for each locus is also reported. *Gga* chrom is the chicken *Gallus gallus* chromosome to which the chicken ortholog of each EST-SSR locus is predicted to map. Within-chromosome position (in base pairs) is also reported. E value is the similarity statistic obtained when each EST-SSR was BLASTed against the chicken genome. Protein match gives the accession number of the peptide within the EnSembl chicken peptide (ftp://ftp.ensembl.org/pub/release-40/gallus_gallus_40_1o/data/fasta/pep/) database that matched the EST-SSR. Exonic region indicates whether the SSR is within the coding region (CDS), the 3’UTR or the 5’UTR.

Loci highlighted in yellow gave identical hits to the named chromosome and also sequence that has currently been assigned to ‘Gga chromosome_random’. Chromosome_random represents sequence that is not yet assigned to any particular chromosome on the current chicken genome assembly. It is likely that the assignment to a ‘true’ chromosome will be confirmed on future assemblies of the chicken genome sequence.

Loci highlighted in bold font were screened in zebra finch and house sparrow populations and are reported in Table 4 of the manuscript.

| **Locus** | **Accession** | **Repeat Type** | **Motif** | **Repeat Length** | **% Purity** | **Max consecutive Repeats** | ***Gga* chrom** | **Position** | **E value** | **Protein Match ( ENSGALP#)** | **Location within Exon** |
| --- | --- | --- | --- | --- | --- | --- | --- | --- | --- | --- | --- |
| CK234301.1 |  | penta- | CCCGG | 20 | 100 | 4 | 20 | 1,662,935 | 7.3E-43 | 00000003175 | 5UTR |
| CK234301.1 |  | tri- | AAG | 24 | 95 | 4 | 20 | 1,662,935 | 7.3E-43 | 00000003175 | CDS |
| CK234324.1 |  | tri- | AGG | 21 | 100 | 7 | 3 | 13,487,929 | 3.4E-20 | 00000032560 | 5UTR |
| CK234328.1 |  | di- | AC | 20 | 95 | <6 |  |  |  |  |  |
| CK234581.1 |  | di- | AT | 20 | 95 | <6 | 6 | 26,143,701 | 6.9E-42 |  |  |
| CK234800.1 |  | di- | AC | 22 | 90 | <6 |  |  |  |  |  |
| CK234814.1 |  | tri- | AGC | 33 | 100 | 11 | 7 | 36,312,896 | 7.7E-37 | 00000020324 | 5UTR |
| CK234875.1 |  | penta- | AGCGG | 20 | 100 | 4 |  |  |  |  |  |
| CK234877.1 |  | tetra- | AAAT | 24 | 100 | 6 |  |  |  |  |  |
| CK235160.1 |  | penta- | AAAAC | 20 | 100 | 4 | 21 | 5,037,633 | 3.7E-51 | 00000022150 | 3UTR |
| CK235805.1 |  | tri- | AAT | 30 | 96 | 6 | 4 | 5,283,096 | 1.3E-63 |  |  |
| CK235818.1 |  | tetra- | ATCC | 20 | 100 | 5 |  |  |  |  |  |
| CK235858.1 |  | penta- | CCCGG | 20 | 100 | 4 | 4 | 16,713,747 | 1.1E-97 | 00000014116 | 5UTR |
| CK301218.1 |  | tri- | AGG | 24 | 100 | 8 |  |  |  |  |  |
| CK301390.1 |  | di- | AT | 22 | 100 | 11 | 1 | 83,904,455 | 1E-143 | 00000024260 | 3UTR |
| CK301407.1 |  | penta- | AGCGG | 25 | 96 | <4 | 1 | 37,112,384 | 2E-65 | 00000016120 | 5UTR |
| CK301512.1 |  | penta- | AAAAC | 20 | 100 | 4 | 1 | 163,900,555 | 3.3E-83 |  |  |
| CK301512.1 |  | di- | AT | 32 | 93 | 8 | 1 | 163,900,555 | 3.3E-83 |  |  |
| CK301536.1 |  | penta- | AAGGG | 30 | 100 | 6 |  |  |  |  |  |
| CK301583.1 |  | di- | AT | 22 | 95 | 6 | 1 | 123,970,315 | 0 |  |  |
| CK301608.1 |  | tri- | AAG | 21 | 95 | <4 | 11 | 17,513,266 | 1E-27 | 00000008785 | CDS |
| CK301722.1 |  | penta- | AAAAC | 20 | 100 | 4 | 14 | 4,738,454 | 4.6E-26 |  |  |
| CK301759.1 |  | di- | AC | 24 | 95 | 7 | 1 | 54,068,469 | 4.2E-14 |  |  |
| CK301777.1 |  | penta- | AAAAC | 25 | 96 | 3 | 14 | 4,110,984 | 2.1E-40 |  |  |
| CK301907.1 |  | tetra- | AGGG | 20 | 100 | 5 | 21 | 629,254 | 2.1E-14 |  |  |
| CK302040.1 |  | tri- | CCG | 21 | 100 | 7 |  |  |  | 00000016899 | CDS |
| CK302109.1 |  | di- | AT | 22 | 95 | <6 | 26 | 1,866,106 | 2.7E-30 |  |  |
| CK302256.1 |  | tetra- | AAAC | 20 | 95 | <4 | 10 | 17,666,409 | 2.9E-77 |  |  |
| CK302333.1 |  | tri- | AGC | 60 | 93 | 7 | 4 | 2,338,328 | 4E-101 |  |  |
| CK302478.1 |  | penta- | AAAAT | 40 | 100 | 8 | Un_random | 30,799,551 | 2.1E-19 | 00000021929 | 3UTR |
| CK302536.1 |  | penta- | AAAAT | 20 | 100 | 4 | 1 | 109,126,492 | 2.3E-59 |  |  |
| CK302671.1 |  | tri- | AGG | 24 | 100 | 8 |  |  |  |  |  |
| CK302794.1 |  | di- | AT | 20 | 100 | 10 |  |  |  |  |  |
| CK302822.1 |  | di- | AC | 24 | 95 | 8 | 14 | 5,256,303 | 3E-12 |  |  |
| CK302859.1 |  | tri- | AAT | 51 | 100 | 17 |  |  |  |  |  |
| CK302873.1 |  | tri- | CCG | 21 | 100 | 7 |  |  |  |  |  |
| CK302873.1 |  | tri- | CCG | 21 | 95 | 7 |  |  |  |  |  |
| CK303059.1 |  | tri- | CCG | 21 | 95 | 5 | 23 | 5,571,978 | 7.6E-17 |  |  |
| CK303130.1 |  | di- | AC | 20 | 95 | <6 | Z | 52,837,232 | 0 |  |  |
| CK303492.1 |  | tri- | AGC | 27 | 100 | 9 | 3 | 113,413,304 | 6.2E-32 | 00000026946 | CDS |
| CK303575.1 |  | tri- | AGC | 33 | 100 | 11 |  |  |  |  |  |
| CK303592.1 |  | tetra- | AATT | 20 | 95 | <4 |  |  |  |  |  |
| CK303727.1 |  | penta- | AAAAT | 115 | 100 | 23 | 5 | 58,484,133 | 5E-23 |  |  |
| CK303727.1 |  | penta- | AAATG | 35 | 100 | 7 | 5 | 58,484,133 | 5E-23 |  |  |
| CK303735.1 |  | tetra- | ACAG | 24 | 100 | 6 | 2 | 60,220,819 | 2.7E-40 | 00000020659 | 5UTR |
| CK304087.1 |  | tri- | ACC | 24 | 100 | 8 | 1 | 96,319,025 | 3.1E-86 | 00000024945 | CDS |
| CK304156.1 |  | di- | AT | 32 | 96 | 9 | 24 | 1,684,275 | 1.7E-35 |  |  |
| CK304183.1 |  | di- | AT | 22 | 95 | 6 |  |  |  |  |  |
| CK304193.1 |  | tri- | CCG | 30 | 100 | 10 |  |  |  |  |  |
| CK304225.1 |  | tri- | CCG | 21 | 95 | 4 |  |  |  |  |  |
| CK304403.1 |  | tri- | AGG | 21 | 95 | <4 | 15 | 5,036,003 | 1.2E-14 |  |  |
| CK304524.1 |  | penta- | AATCC | 60 | 100 | 12 |  |  |  |  |  |
| CK304609.1 |  | tri- | AAT | 24 | 95 | 4 |  |  |  |  |  |
| CK304841.1 |  | di- | AT | 24 | 100 | 12 |  |  |  |  |  |
| CK304843.1 |  | tetra- | AAAT | 24 | 95 | <4 |  |  |  |  |  |
| CK304857.1 |  | di- | AT | 20 | 95 | <6 | 26 | 4,194,760 | 1.7E-78 |  |  |
| CK304920.1 |  | tri- | CCG | 21 | 100 | 7 |  |  |  |  |  |
| **CK304956.1** |  | **di-** | **AT** | **24** | **95** | **8** | **7** | **2,669,411** | **5.9E-35** |  |  |
| CK305005.1 |  | di- | AT | 26 | 100 | 13 |  |  |  |  |  |
| CK305147.1 |  | di- | AC | 20 | 100 | 10 | 1 | 86,285,124 | 1.2E-88 |  |  |
| CK305172.1 |  | penta- | AAAAC | 20 | 95 | 3 | 26 | 3,742,885 | 1.7E-37 |  |  |
| CK305233.1 |  | di- | AT | 24 | 95 | 7 | 2 | 80,089,639 | 3E-160 |  |  |
| CK305335.1 |  | tri- | AAT | 30 | 96 | 5 | 17 | 3,035,817 | 3.6E-33 |  |  |
| CK305630.1 |  | penta- | ACCGG | 20 | 100 | 4 |  |  |  |  |  |
| CK305634.1 |  | tri- | CCG | 21 | 95 | 5 | 4 | 47,898,760 | 2.1E-23 |  |  |
| CK305992.1 |  | tetra- | ACCC | 20 | 100 | 5 |  |  |  |  |  |
| CK306159.1 |  | di- | AT | 22 | 95 | 12 | 2 | 92,921,756 | 6.4E-44 |  |  |
| CK306159.1 |  | di- | AT | 24 | 100 | 12 | 2 | 92,921,756 | 6.4E-44 |  |  |
| CK306250.1 |  | tri- | AGC | 24 | 100 | 8 | 15 | 8,555,890 | 1E-51 | 00000010367 | 3UTR |
| CK306323.1 |  | tri- | AGG | 30 | 96 | 7 | 15 | 8,106,578 | 2.8E-40 | 00000009414 | CDS |
| CK306467.1 |  | tri- | AGG | 27 | 100 | 9 |  |  |  |  |  |
| CK306520.1 |  | tetra- | AAAG | 24 | 100 | 6 | 7 | 19,237,910 | 1.5E-66 | 00000006636 | 3UTR |
| CK306569.1 |  | tetra- | AAAC | 24 | 100 | 6 | 17 | 6,845,794 | 5E-122 |  |  |
| CK306631.1 |  | di- | AT | 32 | 93 | <6 | 1 | 135,576,504 | 6E-103 |  |  |
| CK306636.1 |  | di- | AT | 36 | 94 | 9 |  |  |  |  |  |
| CK306783.1 |  | penta- | AAAAC | 25 | 100 | 5 | 19 | 8,690,121 | 1E-47 | 00000008856 | 3UTR |
| CK306996.1 |  | di- | AT | 26 | 96 | 7 | 4 | 2,460,916 | 3.8E-24 |  |  |
| CK307137.1 |  | penta- | AGGGC | 20 | 100 | 4 | 1 | 647,371 | 4.8E-14 | 00000032751 | 3UTR |
| CK307143.1 |  | penta- | AGCGG | 20 | 100 | 4 | 2 | 26,614,859 | 6.4E-38 | 00000017428 | 5UTR |
| CK307202.1 |  | tri- | CCG | 21 | 90 | 4 | 26 | 3,039,150 | 3.2E-49 | 00000002186 | 5UTR |
| CK307207.1 |  | tri- | CCG | 21 | 90 | <4 | 18 | 5,062,394 | 7.9E-28 | 00000034078 | 5UTR |
| CK307258.1 |  | penta- | CCCCG | 20 | 95 | 3 |  |  |  |  |  |
| CK307877.1 |  | tri- | AGC | 24 | 100 | 8 | Un_random | 5,921,477 | 7E-19 |  |  |
| CK307881.1 |  | penta- | AAGAG | 50 | 92 | <4 |  |  |  |  |  |
| CK307891.1 |  | penta- | AGCCG | 20 | 100 | 4 | 8 | 4,022,456 | 6.5E-29 | 00000004365 | 5UTR |
| CK308028.1 |  | di- | AT | 22 | 90 | 8 | 5 | 35,196,867 | 1E-177 |  |  |
| CK308096.1 |  | di- | AT | 26 | 96 | 6 | 11 | 11,693,857 | 3E-159 |  |  |
| CK308237.1 |  | tetra- | AAAT | 20 | 100 | 5 |  |  |  |  |  |
| CK308660.1 |  | tri- | AAT | 30 | 96 | 5 | 8 | 5,413,143 | 1.9E-16 |  |  |
| CK308723.1 |  | tri- | AAT | 123 | 100 | 41 |  |  |  |  |  |
| CK308861.1 |  | tri- | AGC | 24 | 95 | <4 |  |  |  | 00000000746 | 3UTR |
| CK308923.1 |  | tri- | CCG | 27 | 100 | 9 | 5 | 18,215,352 | 1.4E-60 |  |  |
| CK308927.1 |  | tri- | CCG | 24 | 95 | 5 | 10 | 367,624 | 8.9E-59 | 00000003877 | 5UTR |
| CK308949.1 |  | tetra- | AGGG | 20 | 95 | <4 | 5 | 59,018,935 | 3.8E-73 | 00000019899 | 5UTR |
| CK309076.1 |  | tri- | AGC | 21 | 100 | 7 | 5 | 58,372,341 | 8E-124 | 00000031824 | 5UTR |
| CK309124.1 |  | penta- | CCCGG | 20 | 100 | 4 |  |  |  |  |  |
| CK309134.1 |  | penta- | AAAAC | 20 | 100 | 4 | 5 | 45,846,481 | 1E-100 |  |  |
| CK309141.1 |  | tri- | AGC | 21 | 95 | 4 | 7 | 9,294,216 | 6.5E-41 | 00000012565 | 5UTR |
| CK309206.1 |  | penta- | AAAGG | 25 | 100 | 5 |  |  |  |  |  |
| CK309274.1 |  | penta- | AAAAC | 20 | 100 | 4 |  |  |  |  |  |
| CK309291.1 |  | penta- | CCGCG | 20 | 100 | 4 |  |  |  |  |  |
| CK309291.1 |  | tri- | CCG | 21 | 100 | 7 |  |  |  |  |  |
| CK309310.1 |  | tetra- | AAAG | 20 | 100 | 5 | 13 | 18,491,423 | 5.4E-11 |  |  |
| CK309433.1 |  | di- | AT | 24 | 95 | 7 | 1 | 80,483,435 | 9.1E-16 |  |  |
| CK309486.1 |  | di- | AT | 20 | 95 | 7 | 5 | 15,430,468 | 9.3E-39 |  |  |
| CK309535.1 |  | tri- | AAG | 21 | 100 | 7 | 15 | 9,362,179 | 3.7E-18 | 00000011269 | 3UTR |
| CK309535.1 |  | tri- | AAG | 21 | 95 | 7 | 15 | 9,362,179 | 3.7E-18 | 00000011269 | CDS |
| CK309761.1 |  | tri- | CCG | 24 | 95 | 5 | 2 | 29,772,919 | 9.7E-37 | 00000017640 | 5UTR |
| CK309774.1 |  | penta- | AAAAC | 25 | 100 | 5 |  |  |  |  |  |
| CK309775.1 |  | tri- | AAG | 21 | 100 | 7 | 28 | 1,984,638 | 3.3E-42 |  |  |
| CK309909.1 |  | di- | AT | 22 | 100 | 11 |  |  |  |  |  |
| CK310020.1 |  | penta- | AAAAT | 75 | 100 | 15 |  |  |  |  |  |
| CK310065.1 |  | penta- | AAAAC | 30 | 100 | 6 | 13 | 12,395,333 | 2.4E-62 |  |  |
| CK310112.1 |  | penta- | AAAAT | 20 | 100 | 4 | 3 | 52,256,841 | 0 | 00000022143 | 3UTR |
| CK310166.1 |  | penta- | CCCGG | 20 | 100 | 4 | 1 | 94,575,269 | 1E-73 | 00000009877 | 5UTR |
| CK310299.1 |  | penta- | AAGGG | 20 | 100 | 4 |  |  |  |  |  |
| CK310343.1 |  | penta- | AAAAC | 20 | 95 | <4 | 12 | 15,737,238 | 8.5E-34 | 00000021837 | 5UTR |
| CK310389.1 |  | di- | AT | 20 | 95 | <6 | 20 | 1,393,126 | 2.7E-55 |  |  |
| CK310394.1 |  | tri- | AGC | 51 | 94 | 6 | 2 | 35,137,523 | 0 | 00000018340 | CDS |
| CK310420.1 |  | tetra- | ACGG | 20 | 95 | <4 |  |  |  |  |  |
| CK310425.1 |  | di- | AT | 34 | 94 | 6 |  |  |  |  |  |
| CK310514.1 |  | tetra- | AAAC | 20 | 100 | 5 |  |  |  |  |  |
| CK310672.1 |  | di- | AC | 20 | 95 | <6 | 7 | 17,852,161 | 8E-164 |  |  |
| CK310699.1 |  | di- | AC | 26 | 92 | <6 | 11 | 2,304,447 | 4.4E-68 | 00000004997 | 5UTR |
| CK310730.1 |  | penta- | AGCTC | 20 | 100 | 4 | 23 | 2,701,473 | 6.8E-16 | 00000022147 | 3UTR |
| CK310749.1 |  | tetra- | ATCC | 24 | 95 | 4 | 8 | 24,318,610 | 2E-124 | 00000032444 | 5UTR |
| CK310881.1 |  | penta- | AAGGG | 20 | 100 | 4 |  |  |  |  |  |
| CK310969.1 |  | penta- | AGCCG | 30 | 93 | 3 |  |  |  |  |  |
| CK310969.1 |  | penta- | AGCCG | 100 | 93 | 5 |  |  |  |  |  |
| CK311100.1 |  | tetra- | AAAT | 20 | 100 | 5 | 5 | 15,429,743 | 2E-173 |  |  |
| CK311260.1 |  | di- | AT | 28 | 96 | 7 | 3 | 40,012,285 | 1E-144 | 00000017886 | 3UTR |
| CK311336.1 |  | penta- | ACGGC | 50 | 96 | 6 | 1 | 64,775,928 | 1E-113 | 00000021321 | 5UTR |
| CK311529.1 |  | penta- | AGGGC | 25 | 100 | 5 | 5 | 26,157,169 | 5E-162 | 00000013735 | 5UTR |
| CK311554.1 |  | tetra- | ACAG | 24 | 100 | 6 | 5 | 10,700,501 | 4.5E-45 |  |  |
| CK311674.1 |  | tetra- | AGGG | 20 | 100 | 5 |  |  |  |  |  |
| CK311704.1 |  | tetra- | AAAC | 24 | 100 | 6 |  |  |  |  |  |
| CK311728.1 |  | penta- | AAAAC | 20 | 100 | 4 | 1 | 53,993,159 | 1.5E-35 |  |  |
| CK311745.1 |  | tri- | AAT | 33 | 93 | 4 | 14 | 14,811,183 | 3.6E-39 |  |  |
| CK311766.1 |  | penta- | CCCGG | 25 | 100 | 5 | Z | 70,929,701 | 8.1E-47 | 00000033924 | 5UTR |
| CK311766.1 |  | penta- | CCCCG | 20 | 100 | 4 | Z | 70,929,701 | 8.1E-47 | 00000033924 | 5UTR |
| CK311789.1 |  | tetra- | AAAC | 24 | 100 | 6 |  |  |  |  |  |
| CK312132.1 |  | tri- | CCG | 21 | 100 | 7 |  |  |  |  |  |
| CK312294.1 |  | penta- | AAAAT | 25 | 96 | <4 | 1 | 6,074,177 | 1E-168 |  |  |
| CK312347.1 |  | penta- | AAAGG | 145 | 100 | 29 |  |  |  |  |  |
| CK312577.1 |  | tetra- | AAAC | 24 | 95 | 4 |  |  |  |  |  |
| CK312587.1 |  | di- | AT | 34 | 97 | 12 | 3 | 36,376,463 | 9E-130 |  |  |
| CK312607.1 |  | tri- | AGC | 27 | 100 | 9 | 5 | 58,954,552 | 2.3E-59 |  |  |
| CK312608.1 |  | di- | AT | 32 | 93 | 7 |  |  |  |  |  |
| CK312608.1 |  | di- | AT | 30 | 100 | 15 |  |  |  |  |  |
| CK312668.1 |  | di- | AT | 28 | 96 | 10 | 2 | 33,428,258 | 7.6E-41 |  |  |
| CK312982.1 |  | di- | AT | 30 | 93 | <6 | 7 | 6,174,613 | 2.8E-39 |  |  |
| CK313022.1 |  | di- | AT | 30 | 96 | 10 | 9 | 21,494,997 | 3.8E-70 |  |  |
| CK313312.1 |  | tetra- | AAAC | 20 | 100 | 5 | 3 | 19,761,495 | 1.3E-14 |  |  |
| CK313424.1 |  | tri- | AGG | 24 | 95 | 5 | 5 | 1,595,806 | 4.6E-73 | 00000006751 | CDS |
| **CK313552.1** |  | **tetra-** | **AAAG** | **20** | **95** | **<4** | **7** | **19,407,016** | **1.4E-32** | **00000015531** | **CDS** |
| CK313639.1 |  | penta- | AAAAC | 20 | 95 | 3 |  |  |  |  |  |
| CK313891.1 |  | tri- | AGC | 24 | 100 | 8 |  |  |  |  |  |
| CK314083.1 |  | tri- | AGG | 24 | 95 | 5 | 1 | 139,693,590 | 1E-30 |  |  |
| CK314212.1 |  | tri- | ACC | 21 | 100 | 7 | Z | 13,009,690 | 2E-16 | 00000023930 | CDS |
| CK314276.1 |  | di- | AC | 20 | 95 | 6 | 10 | 1,061,322 | 2.9E-77 |  |  |
| CK314425.1 |  | di- | AT | 32 | 93 | 8 | 5 | 59,348,595 | 3.3E-89 |  |  |
| CK314428.1 |  | di- | AT | 22 | 95 | 6 | 8 | 24,356,390 | 2E-143 | 00000032444 | 3UTR |
| CK314588.1 |  | penta- | AGCGG | 20 | 95 | 3 | Un_random | 33,581,736 | 2.4E-83 | 00000013568 | 5UTR |
| CK314588.1 |  | penta- | AGCGG | 30 | 93 | 4 | Un_random | 33,581,736 | 2.4E-83 | 00000013568 | 5UTR |
| CK314588.1 |  | penta- | CCCGG | 20 | 100 | 4 | Un_random | 33,581,736 | 2.4E-83 | 00000013568 | 5UTR |
| CK314608.1 |  | tri- | CCG | 21 | 95 | <4 |  |  |  |  |  |
| CK314716.1 |  | tri- | AGG | 27 | 92 | <4 | 2 | 129,791,658 | 1E-150 |  |  |
| CK314723.1 |  | penta- | AGAGC | 20 | 100 | 4 |  |  |  | 00000012295 | CDS |
| CK314824.1 |  | tri- | AGC | 21 | 100 | 7 |  |  |  |  |  |
| CK314847.1 |  | penta- | CCCGG | 20 | 100 | 4 | 10 | 21,113,281 | 1.9E-40 | 00000012868 | 5UTR |
| CK314884.1 |  | tri- | AGG | 21 | 100 | 7 |  |  |  |  |  |
| CK314992.1 |  | di- | AC | 22 | 95 | 6 | 1 | 85,582,772 | 1E-58 |  |  |
| CK315036.1 |  | di- | CG | 20 | 100 | 10 | 1 | 104,452,719 | 0 |  |  |
| CK315053.1 |  | di- | AG | 20 | 100 | 10 |  |  |  |  |  |
| CK315131.1 |  | tri- | AGG | 27 | 100 | 9 |  |  |  |  |  |
| CK315233.1 |  | tri- | AGG | 21 | 95 | <4 | 3 | 88,433,747 | 0 | 00000026187 | 5UTR |
| CK315250.1 |  | tri- | AGG | 21 | 95 | 4 | 1 | 51,632,661 | 6E-37 | 00000019492 | 5UTR |
| CK315344.1 |  | di- | AT | 22 | 100 | 11 | 1 | 163,215,271 | 6E-171 |  |  |
| CK315491.1 |  | di- | AT | 38 | 100 | 19 | 12 | 11,302,813 | 7.9E-16 |  |  |
| CK315649.1 |  | di- | AT | 22 | 95 | 7 |  |  |  |  |  |
| CK315726.1 |  | tri- | CCG | 30 | 100 | 10 | 3 | 32,925,813 | 1.3E-85 | 00000017145 | 5UTR |
| CK315950.1 |  | tri- | CCG | 27 | 96 | 6 | 2 | 91,707,968 | 2E-118 | 00000030340 | 3UTR |
| CK315952.1 |  | tri- | ATC | 21 | 95 | <4 |  |  |  |  |  |
| CK315984.1 |  | tri- | CCG | 21 | 100 | 7 |  |  |  |  |  |
| CK316117.1 |  | tri- | AAT | 27 | 96 | 5 | 14 | 4,122,846 | 1.5E-72 |  |  |
| CK316202.1 |  | penta- | AAAAC | 20 | 100 | 4 | 17 | 8,902,297 | 3.3E-98 |  |  |
| CK316314.1 |  | di- | AC | 22 | 100 | 11 |  |  |  |  |  |
| CK316380.1 |  | di- | AG | 20 | 95 | <6 | 4 | 41,462,119 | 1E-159 |  |  |
| CK317200.1 |  | penta- | AAAAC | 50 | 94 | 7 |  |  |  |  |  |
| CK317333.1 |  | di- | AT | 46 | 93 | 10 | 22 | 529,865 | 1.8E-90 |  |  |
| CK317388.1 |  | tetra- | AAAC | 20 | 100 | 5 | 1 | 200,072,220 | 4.1E-20 |  |  |
| CK317536.1 |  | tri- | AAC | 24 | 100 | 8 | 5 | 5,947,809 | 5.8E-23 |  |  |
| CK317544.1 |  | di- | AG | 32 | 93 | 7 | Z | 57,282,873 | 2.8E-46 |  |  |
| CK317545.1 |  | tri- | AAT | 87 | 98 | 25 |  |  |  |  |  |
| DV570793.1 |  | tri- | AGG | 27 | 96 | 5 | 17 | 2,826,759 | 5.3E-18 | 00000011635 | 5UTR |
| DV570808.1 |  | tri- | AGG | 30 | 100 | 10 |  |  |  |  |  |
| DV571687.1 |  | tri- | AAT | 21 | 95 | <4 |  |  |  |  |  |
| DV571740.1 |  | tri- | CCG | 21 | 100 | 7 |  |  |  |  |  |
| DV572340.1 |  | penta- | CCGCG | 20 | 95 | <4 | 5 | 40,540,062 | 9.2E-65 | 00000016737 | CDS |
| DV576713.1 |  | tri- | ATC | 75 | 100 | 25 | 26 | 2,090,305 | 1.4E-60 | 00000027817 | 3UTR |
| DV578095.1 |  | tri- | AGG | 21 | 100 | 7 | 3 | 32,156,617 | 4.4E-42 | 00000016843 | CDS |
| DV579380.1 |  | di- | AC | 24 | 100 | 12 | 4 | 36,788,454 | 1E-106 | 00000016907 | 5UTR |
| DV579388.1 |  | tri- | AGG | 30 | 96 | 5 |  |  |  |  |  |
| DV579388.1 |  | tri- | AGG | 21 | 100 | 7 |  |  |  |  |  |
| DV579597.1 |  | tri- | AGG | 21 | 100 | 7 | 18 | 4,632,346 | 4.1E-36 | 00000003470 | 5UTR |
| DV579750.1 |  | penta- | ACGGC | 20 | 100 | 4 | 1 | 88,656,123 | 4.2E-73 |  |  |
| DV579905.1 |  | tri- | AGC | 36 | 100 | 12 | 17 | 691,674 | 1.2E-51 | 00000014813 | 5UTR |
| DV580335.1 |  | tri- | AGC | 24 | 95 | 5 | 2 | 110,807,446 | 8E-93 | 00000024507 | 5UTR |
| DV581283.1 |  | tetra- | AGCG | 20 | 100 | 5 | 8 | 27,143,410 | 2E-148 |  |  |
| DV582611.1 |  | tri- | CCG | 24 | 95 | <4 |  |  |  |  |  |
| DV582773.1 |  | penta- | ACGGC | 30 | 93 | <4 | 1 | 173,432,892 | 3.8E-67 | 00000027416 | 5UTR |
| DV584100.1 |  | penta- | CCCGG | 25 | 100 | 5 | 1 | 80,303,144 | 2.5E-63 |  |  |
| DV944981.1 |  | di- | AT | 36 | 97 | 9 |  |  |  |  |  |
| DV945041.1 |  | di- | AC | 32 | 100 | 16 | 18 | 4,961,921 | 6.4E-16 |  |  |
| DV945082.1 |  | tri- | AGG | 21 | 100 | 7 | 18 | 9,168,836 | 4.9E-14 |  |  |
| DV945222.1 |  | penta- | AAAAC | 25 | 96 | 4 | 3 | 107,321,477 | 3.9E-23 |  |  |
| DV945287.1 |  | penta- | AAAAC | 20 | 100 | 4 | 1 | 6,369,656 | 5.7E-26 |  |  |
| DV945380.1 |  | tri- | AAT | 42 | 100 | 14 | 18 | 9,708,577 | 4.6E-13 |  |  |
| DV945405.1 |  | tetra- | AACC | 20 | 100 | 5 |  |  |  |  |  |
| DV945417.1 |  | tri- | CCG | 24 | 100 | 8 | 9 | 21,260,711 | 3.2E-39 | 00000015133 | 5UTR |
| DV945670.1 |  | di- | AT | 24 | 100 | 12 | Z | 37,359,037 | 7E-146 |  |  |
| DV945713.1 |  | tetra- | AAAT | 24 | 100 | 6 |  |  |  |  |  |
| DV945744.1 |  | di- | AC | 30 | 100 | 15 |  |  |  |  |  |
| DV945818.1 |  | tri- | AGG | 21 | 100 | 7 | 25 | 888,998 | 6.5E-38 | 00000021582 | 3UTR |
| DV946043.1 |  | tri- | CCG | 21 | 95 | <4 | 11 | 2,137,033 | 1.2E-11 | 00000004839 | 5UTR |
| DV946288.1 |  | di- | AT | 28 | 96 | 7 | 4 | 4,186,430 | 2E-108 | 00000009935 | 3UTR |
| DV946369.1 |  | penta- | AGATG | 100 | 100 | 20 |  |  |  |  |  |
| DV946462.1 |  | tri- | AGG | 24 | 100 | 8 |  |  |  | 00000001784 | 3UTR |
| DV946606.1 |  | penta- | CCCGG | 20 | 100 | 4 | 6 | 11,728,120 | 2.6E-68 | 00000006457 | 5UTR |
| **DV946651.1** |  | **di-** | **AT** | **40** | **95** | **8** | **1** | **71,055,819** | **1.6E-32** |  |  |
| DV946652.1 |  | di- | AC | 20 | 100 | 10 |  |  |  |  |  |
| DV946835.1 |  | di- | AG | 20 | 100 | 10 | 19 | 4,231,031 | 3.9E-41 |  |  |
| DV946910.1 |  | tri- | CCG | 30 | 93 | 5 |  |  |  |  |  |
| DV946910.1 |  | tri- | AGG | 39 | 94 | 5 |  |  |  |  |  |
| DV946995.1 |  | tri- | AGG | 27 | 96 | 5 |  |  |  |  |  |
| DV946995.1 |  | penta- | AGCCG | 20 | 100 | 4 |  |  |  |  |  |
| DV947044.1 |  | tri- | AAT | 21 | 95 | <4 | 8 | 13,001,979 | 1.3E-81 | 00000008774 | 3UTR |
| DV947160.1 |  | tri- | AGG | 24 | 95 | 5 | 2 | 92,587,338 | 3.7E-27 | 00000022145 | 5UTR |
| DV947251.1 |  | di- | AT | 20 | 95 | <6 | 10 | 6,528,512 | 1.5E-41 |  |  |
| DV947577.1 |  | di- | AG | 20 | 100 | 10 |  |  |  |  |  |
| DV947627.1 |  | tri- | AGG | 27 | 96 | 6 |  |  |  |  |  |
| DV947629.1 |  | tri- | CCG | 21 | 100 | 7 | Z | 19,553,396 | 5.1E-20 | 00000023730 | 5UTR |
| DV947660.1 |  | tri- | AGG | 27 | 100 | 9 | 19 | 8,737,881 | 1.6E-59 |  |  |
| DV947669.1 |  | di- | AT | 20 | 100 | 10 | 5 | 3,413,878 | 2.3E-74 |  |  |
| DV947804.1 |  | penta- | CCCGG | 25 | 100 | 5 |  |  |  |  |  |
| DV947923.1 |  | tri- | CCG | 27 | 96 | 6 |  |  |  |  |  |
| DV947938.1 |  | tri- | AGG | 21 | 100 | 7 | 7 | 36,638,086 | 5.6E-41 | 00000020361 | 3UTR |
| DV947947.1 |  | penta- | CCCGG | 20 | 100 | 4 | 2 | 90,873,761 | 6.5E-38 | 00000030346 | 3UTR |
| DV947948.1 |  | tri- | AGG | 21 | 95 | <4 | 3 | 97,427,242 | 8.4E-93 |  |  |
| DV948030.1 |  | penta- | AGAGC | 20 | 100 | 4 | 15 | 6,177,093 | 3.3E-15 |  |  |
| DV948210.1 |  | di- | AT | 28 | 96 | 10 | 7 | 22,591,818 | 1.7E-81 |  |  |
| **DV948303.1** |  | **di-** | **AC** | **44** | **97** | **14** | **6** | **24,725,850** | **3.3E-24** |  |  |
| DV948306.1 |  | penta- | AAAAC | 20 | 95 | <4 | 1 | 69,732,513 | 3.2E-52 |  |  |
| DV948306.1 |  | tetra- | AAAC | 20 | 95 | <4 | 1 | 69,732,513 | 3.2E-52 |  |  |
| DV948378.1 |  | tri- | AAT | 39 | 97 | 7 | 2 | 3,792,167 | 1.9E-47 | 00000008825 | 3UTR |
| DV948447.1 |  | penta- | AAAAC | 20 | 100 | 4 | 1 | 185,467,399 | 4.3E-17 |  |  |
| DV948467.1 |  | di- | AT | 26 | 96 | 7 | 3 | 81,193,225 | 2E-127 |  |  |
| DV948513.1 |  | di- | AC | 24 | 95 | 7 | 15 | 2,649,121 | 2.7E-46 |  |  |
| DV948584.1 |  | tri- | CCG | 21 | 100 | 7 | 4 | 4,003,078 | 5.1E-51 | 00000009819 | 5UTR |
| DV948632.1 |  | tri- | CCG | 21 | 100 | 7 |  |  |  |  |  |
| DV948711.1 |  | penta- | AAAAC | 20 | 100 | 4 | 2 | 114,921,010 | 3E-117 |  |  |
| DV948892.1 |  | di- | AT | 34 | 91 | <6 | 9 | 15,901,361 | 8.1E-99 |  |  |
| DV948966.1 |  | di- | AG | 28 | 96 | 7 | 1 | 94,999,066 | 1E-140 | 00000024864 | 5UTR |
| DV949023.1 |  | tri- | AGG | 21 | 100 | 7 | 14 | 4,930,643 | 2.9E-12 |  |  |
| DV949035.1 |  | di- | AT | 58 | 94 | 8 | Z | 39,991,371 | 4.4E-85 |  |  |
| DV949158.1 |  | tri- | AAT | 27 | 100 | 9 | 4 | 88,229,339 | 1.2E-79 |  |  |
| DV949226.1 |  | penta- | ACGGC | 20 | 100 | 4 |  |  |  |  |  |
| DV949246.1 |  | tri- | CCG | 27 | 100 | 9 | 7 | 4,625,418 | 1.1E-33 | 00000006035 | 5UTR |
| DV949413.1 |  | di- | AT | 24 | 95 | 8 | 4 | 92,084,421 | 8.6E-28 |  |  |
| DV949447.1 |  | tri- | AAT | 72 | 93 | 5 | 2 | 5,170,626 | 3.8E-36 |  |  |
| DV949447.1 |  | di- | AT | 20 | 95 | 10 | 2 | 5,170,626 | 3.8E-36 |  |  |
| DV949447.1 |  | di- | AT | 20 | 100 | 10 | 2 | 5,170,626 | 3.8E-36 |  |  |
| DV949483.1 |  | di- | AC | 28 | 96 | 7 | 7 | 33,025,545 | 4E-45 |  |  |
| DV949559.1 |  | penta- | CCCGG | 25 | 100 | 5 | 13 | 17,763,033 | 1.2E-36 | 00000011819 | 3UTR |
| DV949638.1 |  | penta- | CCCGG | 30 | 96 | 3 | 1 | 117,473,228 | 4E-113 | 00000026188 | 5UTR |
| DV949822.1 |  | tri- | AGC | 21 | 100 | 7 |  |  |  |  |  |
| DV949929.1 |  | tri- | AGC | 27 | 92 | <4 | 20 | 9,503,163 | 1.4E-78 | 00000009725 | 3UTR |
| DV950070.1 |  | di- | AC | 38 | 94 | 9 |  |  |  |  |  |
| DV950084.1 |  | tri- | AGG | 21 | 95 | <4 | 5 | 29,147,153 | 5E-110 | 00000015267 | CDS |
| DV950145.1 |  | penta- | AGCCG | 20 | 100 | 4 | 7 | 14,772,016 | 3.4E-61 | 00000032724 | 5UTR |
| DV950155.1 |  | penta- | AAACC | 35 | 100 | 7 |  |  |  |  |  |
| DV950249.1 |  | tri- | AGG | 27 | 100 | 9 | 2 | 461,011 | 1E-76 |  |  |
| DV950356.1 |  | penta- | AGCCC | 20 | 100 | 4 | 9 | 13,495,454 | 6E-127 |  |  |
| DV950391.1 |  | di- | AT | 32 | 93 | 9 | 1 | 104,459,087 | 0 | 00000028718 | 3UTR |
| DV950668.1 |  | di- | AC | 36 | 91 | 7 | 1 | 149,496,138 | 3.3E-89 |  |  |
| DV950733.1 |  | tetra- | AAAT | 24 | 100 | 6 | 21 | 6,144,119 | 1.2E-45 |  |  |
| DV950818.1 |  | penta- | AAACC | 20 | 100 | 4 |  |  |  |  |  |
| DV950884.1 |  | tri- | AGC | 24 | 100 | 8 | 22 | 441,003 | 9.1E-25 |  |  |
| DV951038.1 |  | penta- | AAAAT | 20 | 100 | 4 | 9 | 23,700,556 | 3.1E-54 |  |  |
| DV951056.1 |  | tri- | CCG | 30 | 96 | 5 | 2 | 89,430,178 | 2E-49 | 00000021522 | CDS |
| DV951067.1 |  | tri- | AGG | 24 | 95 | <4 | 3 | 78,371,851 | 2E-110 | 00000025390 | 5UTR |
| DV951355.1 |  | tetra- | AACG | 24 | 95 | 3 |  |  |  |  |  |
| DV951461.1 |  | penta- | AAGGG | 25 | 100 | 5 |  |  |  | 00000025010 | 3UTR |
| DV951496.1 |  | tri- | AGG | 24 | 95 | 5 | 18 | 4,476,493 | 3.3E-31 | 00000003123 | CDS |
| DV951550.1 |  | tetra- | ATCC | 36 | 100 | 9 |  |  |  |  |  |
| DV951579.1 |  | tri- | CCG | 21 | 95 | 5 | 1 | 95,765,675 | 7.8E-22 | 00000024926 | 5UTR |
| DV951601.1 |  | penta- | AGGGC | 20 | 100 | 4 | 1 | 6,512,313 | 2.3E-28 | 00000022598 | 5UTR |
| DV951916.1 |  | di- | AC | 32 | 96 | 10 |  |  |  |  |  |
| DV951923.1 |  | penta- | ATCCC | 20 | 100 | 4 |  |  |  |  |  |
| DV952025.1 |  | tri- | AAT | 21 | 100 | 7 |  |  |  |  |  |
| DV952112.1 |  | tri- | AGC | 30 | 96 | 6 | 8 | 13,644,225 | 2.9E-86 | 00000008892 | CDS |
| **DV952125.1** |  | **di-** | **AT** | **20** | **100** | **10** | **7** | **21,195,721** | **5.3E-88** |  |  |
| DV952394.1 |  | tri- | AGG | 21 | 95 | <4 |  |  |  |  |  |
| DV952490.1 |  | tri- | AAT | 30 | 100 | 10 |  |  |  |  |  |
| DV952581.1 |  | di- | AG | 22 | 100 | 11 | 3 | 69,886,127 | 3.2E-20 |  |  |
| DV952600.1 |  | tetra- | AGGG | 20 | 95 | <4 | 23 | 64,852 | 2.2E-34 |  |  |
| DV952619.1 |  | tri- | AGG | 24 | 100 | 8 | 26 | 4,193,390 | 1.7E-50 |  |  |
| DV952643.1 |  | tetra- | AAAT | 24 | 100 | 6 |  |  |  |  |  |
| DV952680.1 |  | penta- | CCCGG | 25 | 100 | 5 | 2 | 24,416,622 | 7.1E-87 | 00000011234 | 5UTR |
| DV952792.1 |  | tri- | CCG | 30 | 100 | 10 | 28 | 542,058 | 1.2E-60 | 00000000555 | 5UTR |
| DV952810.1 |  | di- | AT | 24 | 95 | 9 | 6 | 33,812,480 | 4.2E-39 |  |  |
| DV952837.1 |  | tri- | AAT | 36 | 91 | 5 | 5 | 59,347,278 | 4E-33 |  |  |
| DV952847.1 |  | penta- | AGCCC | 25 | 100 | 5 | 14 | 12,577,893 | 1.2E-33 |  |  |
| DV952847.1 |  | penta- | CCCGG | 20 | 100 | 5 | 14 | 12,577,893 | 1.2E-33 |  |  |
| DV952886.1 |  | penta- | AAAAG | 85 | 92 | 9 | Z | 6,876,038 | 1.2E-85 |  |  |
| DV953042.1 |  | tri- | AGC | 24 | 95 | <4 | 20 | 7,687,880 | 2.2E-87 | 00000008211 | 3UTR |
| DV953075.1 |  | penta- | AAAAC | 20 | 100 | 4 | Z | 2,413,451 | 1.8E-38 |  |  |
| DV953173.1 |  | tri- | AGC | 24 | 95 | <4 |  |  |  |  |  |
| DV953416.1 |  | penta- | ACGGC | 20 | 100 | 4 | Un_random | 4,306,530 | 1E-45 |  |  |
| DV953866.1 |  | tri- | ATC | 21 | 95 | <4 | 20 | 507,636 | 1E-110 | 00000001791 | 5UTR |
| DV953928.1 |  | tri- | AGC | 21 | 100 | 7 | 4 | 51,435,841 | 9.3E-40 | 00000018737 | CDS |
| DV954238.1 |  | tri- | AGG | 27 | 100 | 9 | 14 | 315,563 | 4.9E-57 | 00000004649 | 5UTR |
| DV954320.1 |  | tetra- | AGAT | 60 | 100 | 15 |  |  |  |  |  |
| DV954346.1 |  | tri- | CCG | 21 | 95 | 4 |  |  |  |  |  |
| DV954348.1 |  | tri- | CCG | 27 | 96 | 5 | 20 | 10,701,122 | 9.9E-71 | 00000011718 | 5UTR |
| DV954349.1 |  | tri- | CCG | 21 | 100 | 7 | 2 | 88,294,048 | 1.7E-40 | 00000021519 | 5UTR |
| DV954445.1 |  | tri- | AGG | 24 | 100 | 8 | 10 | 14,456,839 | 3E-169 | 00000010517 | 5UTR |
| DV954489.1 |  | tri- | AGG | 21 | 100 | 7 | 28 | 3,840,622 | 8.3E-30 |  |  |
| DV954568.1 |  | tri- | AGG | 21 | 100 | 7 | 18 | 9,884,356 | 1.8E-12 | 00000019147 | CDS |
| DV954733.1 |  | tetra- | AAAC | 20 | 100 | 5 | 14 | 14,333,585 | 6.1E-23 |  |  |
| DV954922.1 |  | penta- | CCCGG | 20 | 100 | 4 |  |  |  |  |  |
| DV954971.1 |  | di- | AT | 24 | 95 | 8 |  |  |  |  |  |
| **DV955012.1** |  | **di-** | **AC** | **22** | **100** | **11** | **7** | **24,798,031** | **8.3E-40** |  |  |
| DV955253.1 |  | di- | AG | 22 | 100 | 11 |  |  |  |  |  |
| DV955333.1 |  | tetra- | AAAC | 20 | 100 | 5 |  |  |  |  |  |
| DV955730.1 |  | tri- | AGG | 33 | 93 | 6 |  |  |  |  |  |
| DV955770.1 |  | tri- | ATC | 21 | 95 | <4 |  |  |  |  |  |
| DV955772.1 |  | tri- | AAT | 30 | 96 | 5 | 23 | 5,315,319 | 1.5E-32 | 00000005159 | 3UTR |
| DV955878.1 |  | penta- | CCCGG | 20 | 100 | 4 | 3 | 70,752,732 | 1E-42 | 00000024715 | 5UTR |
| DV956003.1 |  | tri- | AAT | 30 | 96 | 6 |  |  |  |  |  |
| DV956013.1 |  | tri- | AAG | 21 | 100 | 7 | 6 | 5,485,123 | 6.2E-13 | 00000033611 | 3UTR |
| DV956025.1 |  | penta- | AGCGG | 20 | 100 | 4 | 1 | 48,107,915 | 3.2E-52 |  |  |
| DV956088.1 |  | di- | AC | 28 | 96 | 9 |  |  |  |  |  |
| DV956088.1 |  | di- | AC | 26 | 96 | 8 |  |  |  |  |  |
| DV956106.1 |  | tetra- | AAAC | 28 | 100 | 7 | 6 | 18,669,788 | 7E-47 | 00000009294 | 3UTR |
| DV956212.1 |  | di- | AT | 22 | 100 | 11 | 19 | 9,476,722 | 3.2E-39 |  |  |
| DV956331.1 |  | penta- | ACGGC | 50 | 100 | 10 | Z | 74,124,520 | 8.4E-34 | 00000008610 | 3UTR |
| DV956346.1 |  | tri- | CCG | 21 | 100 | 7 | 10 | 21,230,415 | 0 |  |  |
| DV956391.1 |  | di- | AC | 36 | 100 | 18 | 6 | 24,700,956 | 3.8E-57 |  |  |
| DV956490.1 |  | penta- | AAAAT | 20 | 100 | 4 | 3 | 106,890,896 | 1E-147 |  |  |
| DV956499.1 |  | tri- | CCG | 33 | 93 | 7 | 1 | 92,450,684 | 1.1E-11 |  |  |
| DV956499.1 |  | tetra- | ATCC | 52 | 94 | 3 | 1 | 92,450,684 | 1.1E-11 |  |  |
| DV956516.1 |  | tri- | AGG | 27 | 100 | 9 |  |  |  |  |  |
| DV956571.1 |  | di- | AT | 24 | 95 | <6 |  |  |  |  |  |
| DV956582.1 |  | tetra- | AAAC | 20 | 100 | 5 |  |  |  |  |  |
| DV956723.1 |  | di- | AT | 28 | 92 | <6 |  |  |  |  |  |
| DV956795.1 |  | tri- | AGG | 21 | 100 | 7 | 1 | 116,301,088 | 1.2E-48 | 00000026156 | 5UTR |
| DV956804.1 |  | penta- | ACCGG | 30 | 93 | <4 |  |  |  |  |  |
| DV956852.1 |  | tetra- | AGGG | 24 | 95 | 3 | 1 | 70,700,322 | 1.2E-20 |  |  |
| DV956863.1 |  | penta- | AAAAC | 20 | 100 | 4 |  |  |  |  |  |
| DV957076.1 |  | tetra- | AAAC | 20 | 100 | 5 |  |  |  |  |  |
| DV957260.1 |  | tetra- | AAAC | 20 | 100 | 5 |  |  |  |  |  |
| DV957308.1 |  | penta- | CCCGG | 25 | 100 | 5 | 15 | 4,547,744 | 5.5E-20 | 00000004761 | 5UTR |
| DV957384.1 |  | tetra- | ATCC | 36 | 94 | 5 |  |  |  |  |  |
| DV957408.1 |  | penta- | CCCGG | 20 | 100 | 4 | 20 | 365,688 | 2.6E-15 |  |  |
| DV957552.1 |  | tetra- | AACC | 20 | 100 | 5 | 2 | 34,233,951 | 4.6E-11 |  |  |
| DV957612.1 |  | penta- | AGCCG | 30 | 100 | 6 | 7 | 29,788,565 | 1.7E-56 | 00000019678 | 5UTR |
| DV957622.1 |  | penta- | CCCGG | 20 | 100 | 4 | 5 | 26,759,190 | 2.8E-83 | 00000013981 | 5UTR |
| DV957622.1 |  | tri- | CCG | 21 | 100 | 7 | 5 | 26,759,190 | 2.8E-83 | 00000013981 | 5UTR |
| DV957674.1 |  | di- | AG | 42 | 95 | 10 | 1 | 65,560,167 | 0 |  |  |
| DV957774.1 |  | di- | AT | 22 | 95 | 7 | 5 | 51,923,094 | 3.2E-98 |  |  |
| DV958066.1 |  | tetra- | ACAG | 20 | 100 | 5 | 21 | 1,001,263 | 9.1E-19 |  |  |
| DV958184.1 |  | tetra- | ACCT | 20 | 100 | 5 | 3 | 33,880 | 1.9E-59 | 00000013142 | 3UTR |
| DV958499.1 |  | penta- | ACGGG | 20 | 100 | 4 |  |  |  |  |  |
| DV958521.1 |  | tri- | AGG | 24 | 100 | 8 |  |  |  |  |  |
| DV958593.1 |  | di- | AC | 22 | 95 | 16 | 1 | 139,250,749 | 1.2E-14 |  |  |
| DV958593.1 |  | di- | AT | 32 | 100 | 16 | 1 | 139,250,749 | 1.2E-14 |  |  |
| DV958737.1 |  | tri- | AAT | 24 | 95 | <4 | 8 | 21,647,652 | 2.4E-40 |  |  |
| DV958877.1 |  | di- | AC | 22 | 100 | 11 |  |  |  |  |  |
| DV958928.1 |  | penta- | AATGC | 25 | 96 | <4 | Z | 53,602,285 | 4E-113 |  |  |
| DV958930.1 |  | tetra- | ACAG | 20 | 100 | 5 | 4 | 15,869,234 | 2.8E-83 | 00000013849 | 5UTR |
| DV958940.1 |  | di- | AC | 20 | 100 | 10 | 1 | 50,335,814 | 2.7E-77 |  |  |
| DV959106.1 |  | penta- | AAAAC | 20 | 100 | 4 |  |  |  |  |  |
| DV959185.1 |  | tetra- | ATCC | 24 | 100 | 6 |  |  |  |  |  |
| DV959246.1 |  | di- | AT | 20 | 100 | 10 | 8 | 25,394,799 | 5.8E-26 |  |  |
| DV959250.1 |  | tetra- | AAAC | 20 | 100 | 5 | 1 | 69,734,575 | 6E-69 |  |  |
| DV959340.1 |  | tetra- | AGGC | 24 | 100 | 6 | 9 | 1,876,887 | 2.8E-37 | 00000001975 | CDS |
| DV959466.1 |  | tri- | AGG | 24 | 95 | 4 |  |  |  |  |  |
| DV959480.1 |  | tri- | AGG | 30 | 100 | 10 | 4 | 2,023,762 | 3.5E-58 | 00000007963 | 3UTR |
| DV959535.1 |  | tri- | AGG | 21 | 100 | 7 | 26 | 493,440 | 1.9E-16 |  |  |
| DV959615.1 |  | tri- | ACC | 21 | 95 | <4 | 16 | 74,093 | 1.3E-29 | 00000021670 | CDS |
| DV959702.1 |  | penta- | CCCGG | 20 | 100 | 4 |  |  |  |  |  |
| DV959736.1 |  | tetra- | CCCG | 20 | 95 | <4 | 1 | 58,984,051 | 8.3E-43 | 00000020949 | 5UTR |
| DV959837.1 |  | penta- | AAAAC | 20 | 100 | 4 | 11 | 1,942,038 | 2.3E-31 |  |  |
| DV959874.1 |  | penta- | AAGGG | 20 | 100 | 4 |  |  |  |  |  |
| DV960049.1 |  | tri- | ACC | 21 | 95 | <4 | 24 | 4,739,190 | 6.4E-90 | 00000023468 | 3UTR |
| DV960138.1 |  | tri- | AAT | 45 | 97 | 7 | 5 | 42,880,050 | 6E-170 |  |  |
| DV960219.1 |  | penta- | AAAAC | 25 | 100 | 5 | 8 | 22,638,097 | 1.2E-91 |  |  |
| DV960219.1 |  | penta- | AAAAC | 20 | 100 | 4 | 8 | 22,638,097 | 1.2E-91 |  |  |
| DV960252.1 |  | tetra- | ACGG | 40 | 97 | 6 |  |  |  |  |  |
| DV960514.1 |  | penta- | AAAAC | 20 | 95 | 3 | 14 | 5,027,832 | 9.4E-34 | 00000007928 | 3UTR |
| DV960694.1 |  | penta- | AGCCG | 20 | 100 | 4 | 11 | 9,060,891 | 2.8E-55 | 00000007137 | 5UTR |
| DV960704.1 |  | di- | AT | 24 | 100 | 12 |  |  |  |  |  |
| DV960867.1 |  | tetra- | ACAT | 20 | 100 | 5 |  |  |  |  |  |
| DV961016.1 |  | tri- | AGC | 51 | 100 | 17 | 11 | 21,546,491 | 2E-136 | 00000001007 | CDS |
| DV961064.1 |  | tri- | AAT | 21 | 100 | 7 | Un_random | 13,866,016 | 4.3E-48 |  |  |
| DV961216.1 |  | penta- | AAATC | 20 | 100 | 4 |  |  |  |  |  |
| DV961338.1 |  | penta- | CCCGG | 20 | 100 | 4 | 8 | 3,723,253 | 1.5E-12 | 00000004058 | 5UTR |
| DV961338.1 |  | tri- | CCG | 21 | 95 | <4 | 8 | 3,723,253 | 1.5E-12 | 00000004058 | 5UTR |
| DV961584.1 |  | tri- | ATC | 21 | 95 | <4 | 12 | 2,104,086 | 3.4E-61 | 00000003768 | 3UTR |
| DV961910.1 |  | tri- | ACC | 21 | 100 | 7 |  |  |  |  |  |
| Contig 1 | CK234218.1 | di- | AT | 34 | 91 | 6 | 1 | 83,429,700 | 7.1E-58 |  |  |
| Contig 2 | CK234443.1 | penta- | CCCGG | 20 | 100 | 4 | 7 | 20,036,105 | 5.4E-43 | 00000015733 | 5UTR |
| Contig 3 | CK234612.1 | di- | AC | 24 | 95 | 7 |  |  |  |  |  |
| Contig 4 | CK234925.1 | di- | AC | 22 | 95 | <6 | 4 | 20,952,561 | 1.1E-23 |  |  |
| Contig 6 | CK235034.1 | di- | AG | 24 | 95 | 7 | 4 | 61,990,507 | 2E-139 |  |  |
| Contig 7 | CK235199.1 | penta- | AAAAT | 20 | 100 | 4 |  |  |  |  |  |
| Contig 8 | CK235244.1 | tri- | AAT | 30 | 100 | 10 | 2 | 14,817,407 | 2E-130 |  |  |
| Contig 8 | CK235244.1 | tetra- | AGAT | 64 | 98 | 9 | 2 | 14,817,407 | 2E-130 |  |  |
| Contig 9 | CK235701.1 | di- | AT | 32 | 100 | 16 | 3 | 62,758,279 | 4.1E-48 |  |  |
| Contig 10 | CK301575.1 | tetra- | ACAG | 20 | 100 | 5 | 4 | 12,465,458 | 0 |  |  |
| Contig 11 | CK301687.1 | tri- | CCG | 27 | 100 | 9 | 4 | 32,698,013 | 8.6E-13 | 00000024864 | 3UTR |
| Contig 12 | CK301921.1 | di- | AT | 22 | 100 | 11 |  |  |  |  |  |
| Contig 12 | CK301921.1 | di- | AT | 26 | 100 | 13 |  |  |  |  |  |
| Contig 13 | CK302017.1 | tri- | CCG | 21 | 95 | <4 |  |  |  | 00000026781 | CDS |
| Contig 14 | CK302615.1 | tetra- | CCCG | 20 | 100 | 5 | 2 | 133,802,136 | 7E-143 | 00000006578 | 3UTR |
| Contig 15 | CK302898.1 | tetra- | ACGC | 20 | 100 | 5 | 1 | 116,341,217 | 7E-66 | 00000026159 | 3UTR |
| Contig 16 | CK302941.1 | tetra- | AAAT | 20 | 100 | 5 | 2 | 76,998,280 | 4E-157 |  |  |
| Contig 17 | CK303018.1 | tri- | CCG | 24 | 100 | 8 | Un_random | 11,351,364 | 6.3E-26 | 00000002352 | 5UTR |
| Contig 18 | CK303134.1 | di- | AT | 20 | 95 | <6 | 1 | 63,017,962 | 4E-104 |  |  |
| Contig 19 | CK303222.1 | di- | AT | 34 | 91 | <6 | 3 | 107,122,790 | 3.4E-58 | 00000026566 | 3UTR |
| Contig 20 | CK303298.1 | tri- | CCG | 24 | 100 | 8 | 2 | 41,380,384 | 4.8E-60 | 00000018788 | 5UTR |
| Contig 21 | CK303607.1 | tetra- | AAGG | 24 | 100 | 6 | 7 | 5,185,720 | 3.6E-58 | 00000006428 | 3UTR |
| Contig 22 | CK304247.1 | tri- | AGG | 36 | 94 | 6 | 20 | 10,509,431 | 1.9E-53 |  |  |
| **Contig 23** | **CK304269.1** | **di-** | **AT** | **28** | **96** | **7** | **7** | **22,761,104** | **3.4E-40** |  |  |
| Contig 24 | CK304284.1 | di- | AT | 20 | 95 | <6 | 1 | 55,403,554 | 3.1E-52 |  |  |
| Contig 25 | CK304371.1 | tri- | AGG | 24 | 95 | 5 |  |  |  |  |  |
| Contig 26 | CK304838.1 | tetra- | ACAG | 20 | 100 | 5 |  |  |  | 00000001640 | 3UTR |
| Contig 27 | CK304878.1 | tri- | ATC | 24 | 95 | 4 | 6 | 34,334,962 | 3.8E-24 | 00000010667 | CDS |
| Contig 28 | CK305011.1 | tri- | AAC | 21 | 100 | 7 | E22C19W28_E50C23 | 48,830 | 1.9E-44 |  |  |
| Contig 29 | CK305127.1 | penta- | AAAAT | 25 | 100 | 5 | 9 | 21,246,618 | 2E-171 | 00000015110 | 3UTR |
| Contig 30 | CK305251.1 | penta- | AAAAT | 20 | 100 | 4 | 27 | 3,924,967 | 6.1E-26 | 00000002420 | 3UTR |
| Contig 31 | CK305333.1 | tri- | AAT | 42 | 97 | 10 |  |  |  |  |  |
| Contig 31 | CK305333.1 | tetra- | ACGG | 28 | 100 | 7 |  |  |  |  |  |
| Contig 32 | CK305580.1 | di- | AC | 24 | 95 | 8 | 18 | 4,041,446 | 3.7E-33 |  |  |
| Contig 33 | CK305872.1 | di- | AT | 24 | 95 | 6 | Z | 18,580,528 | 0 |  |  |
| Contig 34 | CK305939.1 | tri- | AGC | 21 | 100 | 7 |  |  |  |  |  |
| **Contig 35** | **CK305949.1** | **di-** | **AG** | **38** | **100** | **19** | **2** | **104,980,774** | **8.7E-90** | **00000023931** | **3UTR** |
| Contig 35 | CK305949.1 | di- | AT | 30 | 93 | 6 | 2 | 104,980,774 | 8.7E-90 | 00000023931 | 3UTR |
| Contig 36 | CK306720.1 | tri- | AGG | 24 | 95 | 5 |  |  |  |  |  |
| Contig 37 | CK306808.1 | di- | AT | 30 | 93 | <6 | 3 | 93,860,678 | 3E-117 |  |  |
| Contig 38 | CK306810.1 | di- | AT | 38 | 97 | 10 | 4 | 11,964,564 | 0 |  |  |
| Contig 39 | CK306857.1 | di- | AT | 26 | 96 | 7 | 3 | 100,058,541 | 4.7E-54 |  |  |
| Contig 40 | CK307120.1 | tri- | AGC | 24 | 100 | 8 | 9 | 25,210,309 | 8.5E-56 | 00000016915 | 5UTR |
| Contig 41 | CK307510.1 | di- | AT | 20 | 100 | 10 | 9 | 18,878,798 | 1.6E-25 |  |  |
| Contig 42 | CK307560.1 | tri- | AGG | 27 | 100 | 9 | 17 | 8,291,338 | 5.3E-88 | 00000003956 | 5UTR |
| Contig 43 | CK307658.1 | di- | AG | 30 | 100 | 15 | Z | 61,092,726 | 0 |  |  |
| Contig 44 | CK308336.1 | penta- | AGCCG | 20 | 100 | 4 | 14 | 14,155,046 | 2.6E-43 | 00000003157 | 5UTR |
| Contig 45 | CK308379.1 | penta- | AAAAC | 20 | 100 | 4 |  |  |  |  |  |
| Contig 46 | CK308733.1 | di- | AT | 20 | 95 | <6 | 7 | 30,505,162 | 3E-49 | 00000019790 | 3UTR |
| Contig 47 | CK308822.1 | di- | AT | 20 | 95 | <6 | 13 | 17,663,398 | 1.7E-81 |  |  |
| Contig 48 | CK308975.1 | penta- | AGGCG | 20 | 100 | 4 | 1 | 171,696,832 | 1.2E-39 | 00000016922 | 5UTR |
| Contig 49 | CK309067.1 | di- | AG | 28 | 96 | 8 | 4 | 11,862,290 | 3E-176 | 00000012289 | 5UTR |
| Contig 49 | CK309067.1 | di- | AG | 28 | 96 | 7 | 4 | 11,862,290 | 3E-176 | 00000012289 | 5UTR |
| Contig 50 | CK309161.1 | tri- | AGC | 27 | 100 | 9 | 2 | 119,338,034 | 3.7E-55 | 00000024980 | CDS |
| Contig 52 | CK309476.1 | penta- | CCCGG | 20 | 100 | 4 | 20 | 5,064,637 | 1.9E-81 | 00000006237 | 5UTR |
| Contig 53 | CK309496.1 | penta- | AAACC | 20 | 100 | 4 | Z | 42,647,956 | 7E-108 |  |  |
| Contig 53 | CK309496.1 | di- | AC | 20 | 95 | <6 | Z | 42,647,956 | 7E-108 |  |  |
| Contig 54 | CK309830.1 | tri- | CCG | 27 | 100 | 5 | 8 | 26,028,699 | 1.9E-47 | 00000017542 | 5UTR |
| Contig 54 | CK309830.1 | tri- | CCG | 27 | 100 | 9 | 8 | 26,028,699 | 1.9E-47 | 00000017542 | 5UTR |
| Contig 55 | CK310190.1 | di- | AT | 68 | 100 | 34 |  |  |  |  |  |
| Contig 56 | CK310280.1 | tetra- | ATCC | 20 | 100 | 5 |  |  |  |  |  |
| Contig 57 | CK310445.1 | penta- | AAAAT | 20 | 100 | 4 | 2 | 128,388,745 | 7E-143 |  |  |
| Contig 58 | CK310497.1 | tri- | CCG | 27 | 100 | 6 |  |  |  |  |  |
| Contig 58 | CK310497.1 | tri- | CCG | 27 | 100 | 9 |  |  |  |  |  |
| Contig 58 | CK310497.1 | tri- | CCG | 39 | 94 | 9 |  |  |  |  |  |
| Contig 58 | CK310497.1 | di- | AG | 22 | 100 | 11 |  |  |  |  |  |
| Contig 59 | CK310576.1 | tetra- | AAAC | 20 | 100 | 5 | 3 | 70,360,628 | 2E-149 |  |  |
| Contig 60 | CK310581.1 | di- | AC | 22 | 100 | 11 |  |  |  |  |  |
| Contig 61 | CK310662.1 | tri- | AGG | 24 | 95 | 5 |  |  |  |  |  |
| Contig 62 | CK310740.1 | penta- | ACCGC | 45 | 100 | 9 |  |  |  |  |  |
| Contig 63 | CK310877.1 | tri- | AGG | 24 | 95 | 5 | 15 | 11,654,627 | 8.3E-25 | 00000013348 | 3UTR |
| Contig 64 | CK311190.1 | tri- | AGG | 21 | 95 | 4 | 12 | 19,978,357 | 3.4E-12 |  |  |
| Contig 65 | CK311294.1 | di- | AT | 24 | 95 | 6 |  |  |  |  |  |
| Contig 66 | CK311416.1 | tetra- | AGGG | 20 | 100 | 5 | 3 | 18,256,462 | 1E-147 | 00000003584 | 5UTR |
| Contig 68 | CK311793.1 | di- | AT | 42 | 92 | 8 | Z | 13,067,682 | 9.4E-53 |  |  |
| Contig 69 | CK311963.1 | tri- | CCG | 27 | 100 | 9 | E22C19W28_E50C23 | 894,348 | 1.4E-29 |  |  |
| Contig 70 | CK312036.1 | penta- | AGGCG | 20 | 100 | 4 | 1 | 23,864,041 | 1.9E-75 | 00000013517 | 5UTR |
| Contig 71 | CK312232.1 | penta- | AAAAC | 25 | 100 | 5 | 5 | 27,300,239 | 7.4E-16 |  |  |
| Contig 72 | CK312436.1 | tri- | AGG | 30 | 96 | 6 | 24 | 127,574 | 8E-69 | 00000016953 | CDS |
| Contig 72 | CK312436.1 | tri- | AGG | 33 | 96 | 6 | 24 | 127,574 | 8E-69 | 00000016953 | CDS |
| **Contig 73** | **CK312585.1** | **di-** | **AT** | **128** | **96** | **16** | **11** | **21,558,373** | **4.8E-81** |  |  |
| Contig 74 | CK312637.1 | tri- | AGC | 33 | 93 | 6 | 18 | 9,014,963 | 6E-171 | 00000007046 | CDS |
| Contig 75 | CK313186.1 | penta- | ACGGC | 20 | 100 | 4 | Z | 74,124,520 | 2.3E-37 | 00000008610 | 3UTR |
| Contig 76 | CK313266.1 | penta- | ACGGC | 65 | 98 | 8 |  |  |  |  |  |
| Contig 77 | CK313422.1 | di- | AT | 24 | 100 | 12 | 13 | 18,850,515 | 2E-143 |  |  |
| Contig 78 | CK313484.1 | tri- | CCG | 27 | 100 | 9 |  |  |  |  |  |
| Contig 79 | CK313798.1 | tri- | AAG | 21 | 100 | 7 | 1 | 65,501,309 | 3.3E-52 |  |  |
| Contig 82 | CK314041.1 | tri- | AGC | 21 | 95 | 5 | Un_random | 58,688,384 | 3.3E-33 | 00000028926 | CDS |
| Contig 83 | CK314156.1 | di- | AT | 74 | 93 | 8 | 1 | 725,367 | 5.6E-97 | 00000012940 | 5UTR |
| Contig 84 | CK314744.1 | tri- | CCG | 24 | 100 | 8 |  |  |  |  |  |
| Contig 84 | CK314744.1 | tri- | CCG | 27 | 96 | 8 |  |  |  |  |  |
| Contig 86 | CK315331.1 | penta- | AAAAT | 25 | 100 | 5 | 2 | 84,559,508 | 1E-70 | 00000021375 | 3UTR |
| Contig 87 | CK315356.1 | tri- | AGG | 21 | 95 | <4 | 1 | 48,528,719 | 3.5E-92 | 00000018726 | 5UTR |
| Contig 88 | CK315728.1 | di- | AT | 36 | 94 | 8 | 6 | 10,668,318 | 2.4E-87 |  |  |
| Contig 89 | CK315752.1 | tri- | ATC | 24 | 100 | 8 |  |  |  | 00000006883 | 5UTR |
| Contig 90 | CK315827.1 | tri- | AGG | 21 | 95 | <4 | 2 | 17,827,820 | 1E-61 | 00000012838 | CDS |
| Contig 91 | CK316017.1 | tri- | AGC | 21 | 95 | <4 | 15 | 9,701,610 | 2.3E-65 | 00000011717 | 3UTR |
| Contig 92 | CK316281.1 | tetra- | ACGG | 20 | 100 | 5 | Z | 59,286,330 | 1.6E-12 |  |  |
| Contig 93 | CK316939.1 | tri- | CCG | 21 | 100 | 7 | Z | 62,412,818 | 8.3E-22 | 00000025111 | CDS |
| Contig 93 | CK316939.1 | tri- | AGG | 27 | 100 | 9 | Z | 62,412,818 | 8.3E-22 | 00000025111 | 5UTR |
| Contig 94 | CK317294.1 | tri- | ACC | 21 | 100 | 7 |  |  |  |  |  |
| Contig 95 | DV571354.1 | penta- | AGCCC | 20 | 100 | 4 | 23 | 545,391 | 9.9E-12 | 00000000868 | 3UTR |
| Contig 96 | DV571518.1 | tetra- | AAAC | 20 | 100 | 5 |  |  |  |  |  |
| Contig 97 | DV571598.1 | di- | AT | 20 | 95 | <6 | 3 | 98,726,135 | 1.1E-79 |  |  |
| Contig 98 | DV571625.1 | di- | AT | 26 | 100 | 13 | 2 | 1,008,845 | 1.6E-35 |  |  |
| Contig 99 | DV571681.1 | tetra- | AAAC | 20 | 100 | 5 |  |  |  |  |  |
| Contig 100 | DV572455.1 | penta- | AGCTC | 20 | 100 | 4 | 23 | 2,702,284 | 9.5E-20 | 00000034494 | 5UTR |
| Contig 100 | DV572455.1 | penta- | AGCTC | 25 | 100 | 5 | 23 | 2,702,284 | 9.5E-20 | 00000034494 | 3UTR |
| Contig 101 | DV572482.1 | tri- | AGC | 27 | 100 | 9 |  |  |  |  |  |
| Contig 102 | DV572626.1 | tri- | ACC | 21 | 100 | 7 | 6 | 23,536,179 | 1E-107 | 00000012074 | 3UTR |
| Contig 105 | DV573670.1 | di- | AT | 22 | 95 | 6 | 3 | 103,032,357 | 0 |  |  |
| Contig 106 | DV573706.1 | tri- | AAC | 21 | 100 | 7 | 7 | 35,035,944 | 2.1E-59 |  |  |
| Contig 107 | DV574334.1 | di- | AG | 24 | 95 | 7 | 21 | 4,111,909 | 3E-35 |  |  |
| Contig 108 | DV575298.1 | di- | AT | 22 | 100 | 11 | 3 | 2,475,751 | 2E-149 |  |  |
| Contig 109 | DV575511.1 | tri- | AAG | 21 | 100 | 7 | 8 | 25,924,283 | 4E-144 | 00000017502 | 5UTR |
| Contig 110 | DV576233.1 | di- | AT | 28 | 96 | 7 | 1 | 45,170,264 | 1.3E-85 |  |  |
| Contig 111 | DV576362.1 | tetra- | AGGG | 20 | 100 | 5 | 1 | 85,638,460 | 3.5E-16 |  |  |
| Contig 112 | DV576598.1 | tri- | AGG | 30 | 96 | 6 |  |  |  | 00000005919 | 3UTR |
| Contig 113 | DV576859.1 | penta- | AAAAC | 20 | 100 | 4 | 4 | 5,172,799 | 9.6E-68 | 00000010910 | 3UTR |
| Contig 114 | DV577165.1 | tetra- | AAAT | 20 | 100 | 5 |  |  |  |  |  |
| Contig 115 | DV577298.1 | di- | AT | 22 | 100 | 11 |  |  |  |  |  |
| Contig 116 | DV577329.1 | penta- | CCCCG | 20 | 100 | 4 | 14 | 1,147,661 | 3E-160 | 00000033669 | 5UTR |
| Contig 117 | DV577718.1 | penta- | AAAAC | 35 | 97 | 4 | 18 | 5,152,373 | 3E-132 |  |  |
| Contig 118 | DV577903.1 | di- | AT | 28 | 92 | 8 | 17 | 5,649,001 | 3.7E-31 |  |  |
| Contig 119 | DV577981.1 | di- | AT | 20 | 95 | <6 |  |  |  |  |  |
| Contig 120 | DV578015.1 | di- | AC | 30 | 100 | 15 |  |  |  |  |  |
| Contig 122 | DV578303.1 | di- | AC | 20 | 95 | <6 | 4 | 35,574,143 | 9E-173 |  |  |
| Contig 123 | DV578368.1 | di- | AT | 28 | 92 | <6 | 2 | 125,046,628 | 4.1E-70 |  |  |
| Contig 124 | DV578549.1 | di- | AC | 46 | 97 | 13 |  |  |  |  |  |
| Contig 125 | DV578839.1 | di- | AT | 26 | 96 | 7 | 1 | 52,919,347 | 3.8E-65 |  |  |
| Contig 126 | DV579042.1 | tri- | AGC | 45 | 95 | 6 | 10 | 7,689,780 | 8E-59 | 00000006517 | 3UTR |
| Contig 127 | DV579052.1 | penta- | AGCCG | 20 | 100 | 4 |  |  |  |  |  |
| Contig 128 | DV579088.1 | di- | AC | 38 | 100 | 19 |  |  |  |  |  |
| Contig 129 | DV579347.1 | di- | AC | 30 | 100 | 15 | 2 | 90,692,704 | 0 |  |  |
| Contig 130 | DV579359.1 | tri- | CCG | 21 | 100 | 7 |  |  |  |  |  |
| Contig 131 | DV579398.1 | di- | AT | 20 | 95 | <6 |  |  |  | 00000003370 | 3UTR |
| Contig 132 | DV579507.1 | di- | AT | 20 | 100 | 10 | 1 | 126,358,615 | 0 |  |  |
| Contig 133 | DV579613.1 | di- | AC | 22 | 95 | <6 | 4 | 1,974,964 | 7.2E-47 |  |  |
| Contig 134 | DV580054.1 | tri- | AGG | 24 | 100 | 8 | 8 | 26,467,725 | 3E-77 |  |  |
| Contig 138 | DV580668.1 | penta- | AAGGG | 20 | 100 | 4 | 3 | 108,667,563 | 1.5E-32 |  |  |
| Contig 139 | DV580854.1 | penta- | AAAAC | 20 | 100 | 4 | 10 | 983,641 | 7.8E-53 |  |  |
| Contig 140 | DV580905.1 | di- | AT | 26 | 96 | 7 | 9 | 24,935,121 | 1.6E-32 |  |  |
| Contig 141 | DV581548.1 | penta- | AAAAC | 20 | 100 | 4 | 13 | 18,144,764 | 3.4E-18 |  |  |
| Contig 142 | DV582035.1 | di- | AT | 20 | 100 | 10 |  |  |  |  |  |
| Contig 143 | DV582287.1 | penta- | AAAAG | 20 | 100 | 4 | 3 | 88,427,734 | 7E-168 |  |  |
| Contig 144 | DV582581.1 | tetra- | AATC | 20 | 100 | 5 | 3 | 39,661,044 | 2.9E-40 | 00000022570 | 5UTR |
| Contig 145 | DV582684.1 | tri- | AGG | 21 | 100 | 7 | 24 | 1,134,031 | 1.2E-54 | 00000001788 | CDS |
| Contig 146 | DV582742.1 | tri- | AAT | 27 | 100 | 9 |  |  |  |  |  |
| Contig 147 | DV582816.1 | penta- | AAAAC | 20 | 100 | 4 |  |  |  |  |  |
| Contig 148 | DV945059.1 | di- | AT | 34 | 91 | <6 | 1 | 6,076,086 | 7E-114 |  |  |
| Contig 149 | DV945088.1 | di- | AT | 36 | 97 | 14 |  |  |  |  |  |
| Contig 150 | DV945440.1 | di- | AT | 24 | 95 | 7 | 2 | 125,741,769 | 3.8E-94 |  |  |
| Contig 151 | DV945550.1 | di- | AT | 20 | 95 | 6 | 15 | 1,065,200 | 0 |  |  |
| Contig 153 | DV945831.1 | tri- | ATC | 54 | 94 | 9 | 2 | 55,517,564 | 2.5E-74 | 00000020269 | 5UTR |
| Contig 154 | DV945884.1 | penta- | AAAAT | 20 | 100 | 4 | 8 | 21,905,931 | 7E-158 |  |  |
| Contig 155 | DV945932.1 | di- | AC | 26 | 100 | 13 | 2 | 101,591,923 | 6.6E-47 |  |  |
| Contig 156 | DV945954.1 | penta- | AGCCG | 40 | 100 | 8 |  |  |  | 00000013425 | 3UTR |
| Contig 157 | DV945999.1 | penta- | AGCCG | 40 | 95 | 6 | 15 | 11,744,336 | 3.2E-15 | 00000013360 | 5UTR |
| Contig 157 | DV945999.1 | penta- | AACCG | 25 | 100 | 6 | 15 | 11,744,336 | 3.2E-15 | 00000013360 | 5UTR |
| Contig 158 | DV946121.1 | penta- | AGCCC | 20 | 100 | 4 |  |  |  |  |  |
| Contig 159 | DV946248.1 | tri- | AGG | 21 | 95 | 4 |  |  |  |  |  |
| Contig 160 | DV946463.1 | tri- | AGG | 21 | 95 | <4 | 13 | 13,299,762 | 1.1E-48 | 00000009080 | 3UTR |
| Contig 161 | DV946483.1 | tri- | AGG | 24 | 100 | 8 | 27 | 4,014,232 | 7.3E-44 | 00000002527 | 3UTR |
| Contig 162 | DV946615.1 | tri- | AGG | 24 | 100 | 8 |  |  |  | 00000015414 | 5UTR |
| Contig 163 | DV946757.1 | tri- | AAC | 21 | 100 | 7 |  |  |  |  |  |
| Contig 164 | DV947051.1 | tri- | CCG | 27 | 100 | 9 | Un_random | 33,309,793 | 3E-108 | 00000022893 | 5UTR |
| Contig 165 | DV947708.1 | penta- | CCCGG | 20 | 100 | 4 | 8 | 4,816,000 | 1.1E-34 | 00000005156 | 3UTR |
| Contig 165 | DV947708.1 | tri- | AAT | 21 | 100 | 7 | 8 | 4,816,000 | 1.1E-34 | 00000005156 | 5UTR |
| Contig 166 | DV947761.1 | tetra- | AAAG | 20 | 95 | <4 | 20 | 4,645,943 | 8E-118 |  |  |
| Contig 167 | DV947884.1 | penta- | AGCGC | 20 | 100 | 4 | 8 | 26,511,463 | 5.9E-60 |  |  |
| Contig 167 | DV947884.1 | penta- | AGCCG | 30 | 100 | 6 | 8 | 26,511,463 | 5.9E-60 |  |  |
| Contig 168 | DV948069.1 | tetra- | ACAG | 20 | 100 | 5 | 8 | 4,338,696 | 1.3E-55 | 00000004817 | 5UTR |
| Contig 169 | DV948092.1 | tri- | AGC | 30 | 100 | 10 | 15 | 4,520,096 | 2E-143 | 00000004589 | 5UTR |
| Contig 170 | DV948238.1 | di- | AT | 20 | 95 | <6 | 26 | 4,192,512 | 7.1E-13 |  |  |
| Contig 171 | DV948560.1 | tetra- | AATC | 24 | 100 | 6 |  |  |  |  |  |
| Contig 172 | DV948691.1 | di- | AT | 36 | 97 | 11 | 13 | 10,457,270 | 3.2E-95 |  |  |
| Contig 173 | DV948792.1 | di- | AT | 20 | 95 | <6 | 1 | 82,767,537 | 6.4E-35 |  |  |
| Contig 174 | DV949123.1 | tri- | ATC | 24 | 100 | 8 | 1 | 112,603,771 | 2.4E-65 |  |  |
| Contig 175 | DV949152.1 | tri- | AGG | 21 | 100 | 7 | 2 | 92,563,415 | 7.3E-75 |  |  |
| Contig 176 | DV949197.1 | tetra- | ATCC | 20 | 100 | 5 |  |  |  |  |  |
| Contig 177 | DV949288.1 | di- | AT | 22 | 100 | 11 | 3 | 76,759,084 | 1.5E-53 |  |  |
| Contig 178 | DV949379.1 | penta- | ACGGC | 45 | 97 | 5 | 1 | 189,910,066 | 2E-103 | 00000027765 | 5UTR |
| Contig 179 | DV949669.1 | tetra- | ATCC | 24 | 100 | 6 |  |  |  |  |  |
| Contig 180 | DV950141.1 | di- | AG | 20 | 100 | 10 | Un_random | 20,746,103 | 2.7E-11 |  |  |
| Contig 181 | DV950251.1 | penta- | AAAAC | 20 | 100 | 4 | 21 | 2,315,325 | 5E-147 |  |  |
| Contig 182 | DV950317.1 | tri- | AAT | 30 | 96 | 6 | 1 | 94,986,105 | 2.9E-89 |  |  |
| Contig 183 | DV950453.1 | penta- | AAAAC | 20 | 100 | 4 | 1 | 83,232,076 | 1.3E-51 | 00000029840 | 3UTR |
| Contig 184 | DV950591.1 | di- | AG | 30 | 96 | 9 | 4 | 11,963,669 | 1.5E-85 |  |  |
| Contig 185 | DV951288.1 | tetra- | AAAC | 28 | 96 | 5 | 15 | 10,657,612 | 4.4E-91 |  |  |
| Contig 186 | DV952279.1 | tri- | AGG | 33 | 96 | 6 |  |  |  |  |  |
| Contig 187 | DV952582.1 | tri- | AGG | 24 | 95 | 4 | 3 | 29,001,198 | 1.8E-25 | 00000016309 | CDS |
| Contig 188 | DV952773.1 | penta- | AGCCG | 55 | 98 | 8 | 10 | 22,118,455 | 4.1E-33 | 00000013428 | 5UTR |
| Contig 189 | DV953425.1 | tri- | AGG | 21 | 95 | <4 | 12 | 2,471,868 | 8.5E-53 | 00000004634 | CDS |
| Contig 190 | DV953459.1 | tetra- | AAGG | 20 | 95 | 3 | 14 | 4,805,405 | 8.8E-59 | 00000007751 | 3UTR |
| Contig 191 | DV953675.1 | di- | AT | 32 | 96 | 10 | 12 | 15,500,052 | 5.8E-91 | 00000012418 | 3UTR |
| Contig 192 | DV954072.1 | penta- | AAAAC | 30 | 93 | 3 |  |  |  |  |  |
| Contig 193 | DV954258.1 | tri- | AAC | 24 | 95 | 4 | 18 | 1,688,772 | 7E-133 |  |  |
| Contig 194 | DV954285.1 | tetra- | ACAG | 20 | 100 | 5 | 4 | 9,133,705 | 3.7E-76 |  |  |
| Contig 195 | DV954446.1 | di- | AC | 22 | 100 | 11 | 9 | 5,467,408 | 2.7E-33 |  |  |
| Contig 197 | DV955836.1 | tri- | AGC | 21 | 100 | 7 | 17 | 5,837,317 | 5.5E-72 | 00000007476 | 5UTR |
| Contig 197 | DV955836.1 | tri- | AGC | 21 | 100 | 7 | 17 | 5,837,317 | 5.5E-72 | 00000007476 | 5UTR |
| Contig 198 | DV956262.1 | di- | AG | 22 | 95 | 6 | 11 | 20,916,517 | 2.5E-31 | 00000000933 | 3UTR |
| Contig 199 | DV956700.1 | tri- | ATC | 24 | 95 | <4 | 10 | 8,313,724 | 2.5E-43 | 00000006826 | CDS |
| Contig 200 | DV957188.1 | tri- | CCG | 30 | 96 | 6 | Un_random | 49,586,192 | 1.4E-28 |  |  |
| Contig 201 | DV957706.1 | di- | AC | 22 | 100 | 11 | 19 | 6,734,266 | 4.7E-42 |  |  |
| Contig 202 | DV958255.1 | penta- | CCCGG | 20 | 100 | 4 |  |  |  |  |  |
| Contig 202 | DV958255.1 | tetra- | ACCC | 20 | 100 | 5 |  |  |  |  |  |
| Contig 203 | DV958291.1 | di- | AT | 20 | 95 | <6 | 1 | 101,715,059 | 1E-107 |  |  |
| Contig 204 | DV958959.1 | tri- | CCG | 21 | 95 | <4 | 1 | 14,834,734 | 1.2E-42 | 00000013230 | 5UTR |
| Contig 205 | DV958965.1 | tetra- | ACAG | 32 | 96 | 5 | 21 | 1,437,173 | 3.2E-46 | 00000001774 | 5UTR |
| Contig 205 | DV958965.1 | tetra- | ACAG | 32 | 96 | 5 | 21 | 1,437,173 | 3.2E-46 | 00000001774 | 5UTR |
| **Contig 206** | **DV959049.1** | **di-** | **AG** | **62** | **100** | **31** | **Z** | **61,092,726** | **1.1E-79** |  |  |
| Contig 207 | DV959521.1 | tri- | AGG | 27 | 100 | 9 | 9 | 22,163,296 | 1.9E-81 | 00000015413 | 5UTR |
| Contig 207 | DV959521.1 | tri- | AGC | 21 | 100 | 7 | 9 | 22,163,296 | 1.9E-81 | 00000015413 | 5UTR |
| Contig 208 | DV959830.1 | penta- | AAAAT | 35 | 94 | 3 |  |  |  |  |  |
| Contig 209 | DV960302.1 | tri- | CCG | 21 | 95 | 4 |  |  |  |  |  |
| Contig 210 | DV960753.1 | tri- | ATC | 21 | 95 | <4 | 2 | 55,424,473 | 1.2E-57 | 00000020236 | CDS |
| Contig 211 | DV961336.1 | tri- | AGC | 21 | 100 | 7 | 27 | 4,486,525 | 3E-169 |  |  |
| Contig 212 | DV961773.1 | di- | AT | 22 | 100 | 11 | 2 | 56,801,228 | 2.6E-37 |  |  |
