# Supplementary material for: Simple sequence repeats in zebra finch (Taeniopygia guttata) expressed sequence tags: a new resource for evolutionary genetic studies of passerines
Source: BMC Genomics. 2007 Feb 14;8:52. doi: 10.1186/1471-2164-8-52 (PMC1804275; doi:10.1186/1471-2164-8-52)
Supplement: Additional File 2 — Sequences containing uninterrupted microsatellties of at least 5 repeats long. Details of all identified EST-SSRs with a minimum of five consecutive perfect repeat units. GenBank Accession numbers, and motif type and length are reported. Putatively polymorphic loci in zebra finch are indicated. [file 1471-2164-8-52-S2.doc]

**Additional File 2:**

**Sequences containing uninterrupted microsatellties of at least 5 repeats long.**

An alternative search strategy for identifying microsatellties is to identify all ESTs with at least 5 consecutive uninterrupted repeats. Performing this search on the EST dataset identified 1536 sequences with repeats, of which 1097 could be grouped into 229 contigs and the remaining 439 were singletons. Among the contigs, polymorphism can be inferred if the repeat length varies between sequences. For example, Contig 8 comprises 2 sequences, one with a 13 repeat unit microsatellite and the other with a 16 repeat unit microsatellite. In contrast, Contig 1 comprises 2 sequences both with a repeat 6 units long. Contigs where repeat unit lenth polymorphism is detectable *in silico* are shown in grey shaded rows. Different shades of grey are simply used to demark consecutive polymorphic contigs – the intensity of shading carries no other meaning.

91/229 (39.7%) of contigs exhibit repeat unit length polymorphism.

52/87 (59.8%) of contigs constructed from 4 or more overlapping sequences exhibit repeat unit length polymorphism.

Note that contig number in this dataset does not correspond to Contig number in Additional File 1.

| **Locus** | **Contig** | **Repeat Type** | **Motif** | **Length (bp)** | **Length (repeats)** |
| --- | --- | --- | --- | --- | --- |
| CK234218.1 | Contig 1 | Dinucleotide | AT | 12 | 6 |
| CK235066.1 | Contig 1 | Dinucleotide | AT | 12 | 6 |
| CK234930.1 | Contig 2 | Trinucleotide | AGG | 18 | 6 |
| CK311771.1 | Contig 2 | Trinucleotide | AGG | 18 | 6 |
| CK234720.1 | Contig 3 | pentanucleotide | ACGGC | 55 | 11 |
| CK235034.1 | Contig 3 | Dinucleotide | AG | 14 | 7 |
| CK235637.1 | Contig 3 | Dinucleotide | AG | 14 | 7 |
| CK302170.1 | Contig 3 | Dinucleotide | AG | 14 | 7 |
| CK316408.1 | Contig 3 | Dinucleotide | AG | 14 | 7 |
| CK235244.1 | Contig 4 | Trinucleotide | AAT | 21 | 7 |
| CK235244.1 | Contig 4 | Tetranucleotide | AGAT | 64 | 16 |
| CK302330.1 | Contig 4 | Trinucleotide | AAT | 30 | 10 |
| CK302330.1 | Contig 4 | Tetranucleotide | AGAT | 36 | 9 |
| CK235482.1 | Contig 5 | Dinucleotide | AG | 12 | 6 |
| DV954699.1 | Contig 5 | Dinucleotide | AG | 12 | 6 |
| CK235484.1 | Contig 6 | Dinucleotide | AT | 14 | 7 |
| CK235522.1 | Contig 6 | Dinucleotide | AT | 14 | 7 |
| CK235595.1 | Contig 7 | Dinucleotide | AT | 12 | 6 |
| CK313232.1 | Contig 7 | Dinucleotide | AT | 12 | 6 |
| CK235701.1 | Contig 8 | Dinucleotide | AT | 26 | 13 |
| DV950416.1 | Contig 8 | Dinucleotide | AT | 32 | 16 |
| CK235786.1 | Contig 9 | Dinucleotide | AG | 12 | 6 |
| DV945196.1 | Contig 9 | dinucleotide | AG | 12 | 6 |
| CK235829.1 | Contig 10 | dinucleotide | AC | 18 | 9 |
| DV945688.1 | Contig 10 | dinucleotide | AC | 18 | 9 |
| CK301681.1 | Contig 11 | dinucleotide | AG | 18 | 9 |
| DV952810.1 | Contig 11 | dinucleotide | AG | 18 | 9 |
| CK301687.1 | Contig 12 | trinucleotide | CCG | 27 | 9 |
| CK315892.1 | Contig 12 | trinucleotide | CCG | 24 | 8 |
| CK316326.1 | Contig 12 | trinucleotide | CCG | 27 | 9 |
| DV946499.1 | Contig 12 | trinucleotide | CCG | 27 | 9 |
| DV953952.1 | Contig 12 | trinucleotide | CCG | 27 | 9 |
| CK301921.1 | Contig 13 | dinucleotide | AT | 22 | 11 |
| CK301921.1 | Contig 13 | dinucleotide | AT | 16 | 8 |
| CK308629.1 | Contig 13 | dinucleotide | AT | 26 | 13 |
| CK308629.1 | Contig 13 | dinucleotide | AT | 18 | 9 |
| CK302013.1 | Contig 14 | dinucleotide | AT | 14 | 7 |
| CK308028.1 | Contig 14 | dinucleotide | AT | 16 | 8 |
| CK301861.1 | Contig 15 | dinucleotide | AG | 12 | 6 |
| CK302282.1 | Contig 15 | dinucleotide | AG | 12 | 6 |
| CK306157.1 | Contig 15 | dinucleotide | AG | 12 | 6 |
| DV581703.1 | Contig 15 | dinucleotide | AG | 12 | 6 |
| CK302810.1 | Contig 16 | dinucleotide | CG | 18 | 9 |
| CK306189.1 | Contig 16 | dinucleotide | CG | 18 | 9 |
| CK309379.1 | Contig 16 | dinucleotide | CG | 18 | 9 |
| CK310552.1 | Contig 16 | dinucleotide | CG | 18 | 9 |
| CK312366.1 | Contig 16 | dinucleotide | CG | 18 | 9 |
| CK312387.1 | Contig 16 | dinucleotide | CG | 18 | 9 |
| CK312480.1 | Contig 16 | dinucleotide | CG | 18 | 9 |
| CK312769.1 | Contig 16 | dinucleotide | CG | 18 | 9 |
| CK313654.1 | Contig 16 | dinucleotide | CG | 18 | 9 |
| CK315036.1 | Contig 16 | dinucleotide | CG | 20 | 10 |
| CK315047.1 | Contig 16 | dinucleotide | CG | 18 | 9 |
| CK315548.1 | Contig 16 | dinucleotide | CG | 18 | 9 |
| CK316113.1 | Contig 16 | dinucleotide | CG | 18 | 9 |
| CK316179.1 | Contig 16 | dinucleotide | CG | 18 | 9 |
| CK317178.1 | Contig 16 | dinucleotide | CG | 18 | 9 |
| DV945026.1 | Contig 16 | dinucleotide | CG | 18 | 9 |
| DV945682.1 | Contig 16 | dinucleotide | CG | 18 | 9 |
| DV945928.1 | Contig 16 | dinucleotide | CG | 18 | 9 |
| DV946525.1 | Contig 16 | dinucleotide | CG | 18 | 9 |
| DV947521.1 | Contig 16 | dinucleotide | CG | 18 | 9 |
| DV947900.1 | Contig 16 | dinucleotide | CG | 18 | 9 |
| DV948759.1 | Contig 16 | dinucleotide | CG | 18 | 9 |
| DV948866.1 | Contig 16 | dinucleotide | CG | 18 | 9 |
| DV952161.1 | Contig 16 | dinucleotide | CG | 18 | 9 |
| DV952826.1 | Contig 16 | dinucleotide | CG | 18 | 9 |
| DV956748.1 | Contig 16 | dinucleotide | CG | 18 | 9 |
| DV960932.1 | Contig 16 | dinucleotide | CG | 18 | 9 |
| DV961777.1 | Contig 16 | dinucleotide | CG | 18 | 9 |
| CK302852.1 | Contig 17 | dinucleotide | AT | 12 | 6 |
| CK317028.1 | Contig 17 | dinucleotide | AT | 12 | 6 |
| CK303018.1 | Contig 18 | trinucleotide | CCG | 24 | 8 |
| CK314647.1 | Contig 18 | trinucleotide | CCG | 24 | 8 |
| CK303190.1 | Contig 19 | dinucleotide | AC | 12 | 6 |
| CK311802.1 | Contig 19 | dinucleotide | AC | 12 | 6 |
| CK303217.1 | Contig 20 | dinucleotide | AC | 12 | 6 |
| DV572716.1 | Contig 20 | dinucleotide | AC | 12 | 6 |
| DV572717.1 | Contig 20 | dinucleotide | AC | 12 | 6 |
| DV572718.1 | Contig 20 | dinucleotide | AC | 12 | 6 |
| DV572719.1 | Contig 20 | dinucleotide | AC | 12 | 6 |
| DV956593.1 | Contig 20 | dinucleotide | AC | 12 | 6 |
| CK303248.1 | Contig 21 | dinucleotide | AC | 16 | 8 |
| CK306036.1 | Contig 21 | dinucleotide | AC | 18 | 9 |
| CK313941.1 | Contig 21 | dinucleotide | AC | 16 | 8 |
| DV571125.1 | Contig 21 | dinucleotide | AC | 16 | 8 |
| DV571126.1 | Contig 21 | dinucleotide | AC | 16 | 8 |
| DV579636.1 | Contig 21 | dinucleotide | AC | 16 | 8 |
| DV579637.1 | Contig 21 | dinucleotide | AC | 16 | 8 |
| DV582234.1 | Contig 21 | dinucleotide | AC | 16 | 8 |
| DV582235.1 | Contig 21 | dinucleotide | AC | 16 | 8 |
| DV582236.1 | Contig 21 | dinucleotide | AC | 16 | 8 |
| DV582237.1 | Contig 21 | dinucleotide | AC | 16 | 8 |
| DV584054.1 | Contig 21 | dinucleotide | AC | 16 | 8 |
| DV584055.1 | Contig 21 | dinucleotide | AC | 16 | 8 |
| DV584056.1 | Contig 21 | dinucleotide | AC | 16 | 8 |
| DV584057.1 | Contig 21 | dinucleotide | AC | 16 | 8 |
| DV584060.1 | Contig 21 | dinucleotide | AC | 16 | 8 |
| DV584061.1 | Contig 21 | dinucleotide | AC | 16 | 8 |
| CK303298.1 | Contig 22 | trinucleotide | CCG | 24 | 8 |
| DV955893.1 | Contig 22 | trinucleotide | CCG | 30 | 10 |
| CK234347.1 | Contig 23 | dinucleotide | AC | 14 | 7 |
| CK302307.1 | Contig 23 | dinucleotide | AC | 14 | 7 |
| CK303773.1 | Contig 23 | dinucleotide | AC | 14 | 7 |
| CK306284.1 | Contig 23 | dinucleotide | AC | 14 | 7 |
| CK312689.1 | Contig 23 | dinucleotide | AC | 14 | 7 |
| CK314488.1 | Contig 23 | dinucleotide | AC | 14 | 7 |
| CK314490.1 | Contig 23 | dinucleotide | AC | 14 | 7 |
| DV952702.1 | Contig 23 | dinucleotide | AC | 14 | 7 |
| DV953442.1 | Contig 23 | dinucleotide | AC | 14 | 7 |
| DV957829.1 | Contig 23 | dinucleotide | AC | 14 | 7 |
| DV960533.1 | Contig 23 | dinucleotide | AC | 14 | 7 |
| CK303998.1 | Contig 24 | dinucleotide | AT | 14 | 7 |
| CK316684.1 | Contig 24 | dinucleotide | AT | 12 | 6 |
| CK304043.1 | Contig 25 | dinucleotide | AG | 16 | 8 |
| CK313100.1 | Contig 25 | dinucleotide | AG | 16 | 8 |
| CK315367.1 | Contig 25 | dinucleotide | AG | 16 | 8 |
| DV573311.1 | Contig 25 | dinucleotide | AG | 18 | 9 |
| DV573312.1 | Contig 25 | dinucleotide | AG | 18 | 9 |
| DV573313.1 | Contig 25 | dinucleotide | AG | 18 | 9 |
| DV573314.1 | Contig 25 | dinucleotide | AG | 18 | 9 |
| DV573315.1 | Contig 25 | dinucleotide | AG | 16 | 8 |
| DV573316.1 | Contig 25 | dinucleotide | AG | 16 | 8 |
| DV577832.1 | Contig 25 | dinucleotide | AG | 16 | 8 |
| DV577833.1 | Contig 25 | dinucleotide | AG | 16 | 8 |
| DV581340.1 | Contig 25 | dinucleotide | AG | 16 | 8 |
| DV581341.1 | Contig 25 | dinucleotide | AG | 16 | 8 |
| CK304064.1 | Contig 26 | dinucleotide | AT | 14 | 7 |
| DV959730.1 | Contig 26 | dinucleotide | AT | 14 | 7 |
| CK304132.1 | Contig 27 | dinucleotide | AT | 14 | 7 |
| CK306367.1 | Contig 27 | dinucleotide | AT | 14 | 7 |
| CK306703.1 | Contig 27 | dinucleotide | AT | 18 | 9 |
| CK312060.1 | Contig 27 | dinucleotide | AT | 18 | 9 |
| DV579789.1 | Contig 27 | dinucleotide | AT | 18 | 9 |
| DV953876.1 | Contig 27 | dinucleotide | AT | 14 | 7 |
| CK304141.1 | Contig 28 | dinucleotide | AC | 14 | 7 |
| DV949115.1 | Contig 28 | dinucleotide | AC | 14 | 7 |
| CK304193.1 | Contig 29 | trinucleotide | CCG | 30 | 10 |
| CK304193.1 | Contig 29 | trinucleotide | CCG | 18 | 6 |
| DV947955.1 | Contig 29 | trinucleotide | CCG | 18 | 6 |
| DV951769.1 | Contig 29 | trinucleotide | CCG | 18 | 6 |
| DV954180.1 | Contig 29 | trinucleotide | CCG | 18 | 6 |
| CK304260.1 | Contig 30 | dinucleotide | AC | 12 | 6 |
| CK304299.1 | Contig 30 | dinucleotide | AC | 12 | 6 |
| CK304269.1 | Contig 31 | dinucleotide | AT | 14 | 7 |
| CK305865.1 | Contig 31 | dinucleotide | AT | 14 | 7 |
| CK309886.1 | Contig 31 | dinucleotide | AT | 16 | 8 |
| DV952809.1 | Contig 31 | dinucleotide | AT | 14 | 7 |
| CK304603.1 | Contig 32 | dinucleotide | AT | 14 | 7 |
| CK311849.1 | Contig 32 | dinucleotide | AT | 14 | 7 |
| CK304776.1 | Contig 33 | dinucleotide | AT | 16 | 8 |
| DV951101.1 | Contig 33 | dinucleotide | AT | 18 | 9 |
| DV951438.1 | Contig 33 | dinucleotide | AT | 18 | 9 |
| DV952125.1 | Contig 33 | dinucleotide | AT | 20 | 10 |
| CK304986.1 | Contig 34 | dinucleotide | AT | 12 | 6 |
| CK308865.1 | Contig 34 | dinucleotide | AT | 12 | 6 |
| CK316195.1 | Contig 34 | dinucleotide | AT | 12 | 6 |
| DV575071.1 | Contig 34 | dinucleotide | AT | 12 | 6 |
| DV575072.1 | Contig 34 | dinucleotide | AT | 12 | 6 |
| CK305011.1 | Contig 35 | trinucleotide | AAC | 21 | 7 |
| CK310973.1 | Contig 35 | trinucleotide | AAC | 21 | 7 |
| CK305093.1 | Contig 36 | dinucleotide | AC | 18 | 9 |
| CK310581.1 | Contig 36 | dinucleotide | AC | 20 | 10 |
| DV954191.1 | Contig 36 | dinucleotide | AC | 22 | 11 |
| CK305190.1 | Contig 37 | dinucleotide | AC | 14 | 7 |
| CK305568.1 | Contig 37 | dinucleotide | AC | 14 | 7 |
| CK305195.1 | Contig 38 | dinucleotide | AG | 14 | 7 |
| DV952887.1 | Contig 38 | dinucleotide | AG | 14 | 7 |
| CK305333.1 | Contig 39 | trinucleotide | AAT | 21 | 7 |
| CK305333.1 | Contig 39 | tetranucleotide | ACGG | 28 | 7 |
| DV951041.1 | Contig 39 | trinucleotide | AAT | 30 | 10 |
| DV951041.1 | Contig 39 | trinucleotide | AAT | 18 | 6 |
| DV951041.1 | Contig 39 | tetranucleotide | ACGG | 24 | 6 |
| CK305384.1 | Contig 40 | trinucleotide | AAT | 18 | 6 |
| DV948235.1 | Contig 40 | trinucleotide | AAT | 18 | 6 |
| CK305476.1 | Contig 41 | dinucleotide | AT | 14 | 7 |
| CK310572.1 | Contig 41 | dinucleotide | AT | 12 | 6 |
| CK305580.1 | Contig 42 | dinucleotide | AC | 24 | 12 |
| CK315627.1 | Contig 42 | dinucleotide | AC | 16 | 8 |
| CK305872.1 | Contig 43 | dinucleotide | AT | 12 | 6 |
| DV960355.1 | Contig 43 | dinucleotide | AT | 14 | 7 |
| DV960355.1 | Contig 43 | dinucleotide | AT | 12 | 6 |
| CK305939.1 | Contig 44 | trinucleotide | AGC | 21 | 7 |
| DV946208.1 | Contig 44 | trinucleotide | AGC | 21 | 7 |
| DV947697.1 | Contig 44 | trinucleotide | AGC | 21 | 7 |
| DV948375.1 | Contig 44 | trinucleotide | AGC | 18 | 6 |
| DV948492.1 | Contig 44 | trinucleotide | AGC | 18 | 6 |
| DV956415.1 | Contig 44 | trinucleotide | AGC | 18 | 6 |
| DV956906.1 | Contig 44 | trinucleotide | AGC | 18 | 6 |
| CK303710.1 | Contig 45 | dinucleotide | AG | 38 | 19 |
| CK305949.1 | Contig 45 | dinucleotide | AG | 34 | 17 |
| CK307697.1 | Contig 45 | dinucleotide | AG | 42 | 21 |
| CK307697.1 | Contig 45 | dinucleotide | AT | 12 | 6 |
| CK310579.1 | Contig 45 | dinucleotide | AG | 38 | 19 |
| CK310579.1 | Contig 45 | dinucleotide | AT | 12 | 6 |
| DV958771.1 | Contig 45 | dinucleotide | AG | 38 | 19 |
| DV958771.1 | Contig 45 | dinucleotide | AT | 12 | 6 |
| DV961913.1 | Contig 45 | dinucleotide | AT | 12 | 6 |
| CK306162.1 | Contig 46 | dinucleotide | AT | 12 | 6 |
| CK316875.1 | Contig 46 | dinucleotide | AT | 12 | 6 |
| CK306322.1 | Contig 47 | dinucleotide | AC | 12 | 6 |
| CK317073.1 | Contig 47 | dinucleotide | AC | 12 | 6 |
| CK306646.1 | Contig 48 | dinucleotide | AC | 16 | 8 |
| DV950115.1 | Contig 48 | dinucleotide | AC | 14 | 7 |
| CK306757.1 | Contig 49 | dinucleotide | AT | 14 | 7 |
| CK316994.1 | Contig 49 | dinucleotide | AT | 14 | 7 |
| DV947449.1 | Contig 49 | dinucleotide | AT | 14 | 7 |
| DV953969.1 | Contig 49 | dinucleotide | AT | 14 | 7 |
| CK302942.1 | Contig 50 | dinucleotide | AT | 20 | 10 |
| CK302942.1 | Contig 50 | dinucleotide | AT | 16 | 8 |
| CK306810.1 | Contig 50 | dinucleotide | AT | 16 | 8 |
| CK306810.1 | Contig 50 | dinucleotide | AT | 20 | 10 |
| CK309573.1 | Contig 50 | dinucleotide | AT | 20 | 10 |
| CK309573.1 | Contig 50 | dinucleotide | AT | 16 | 8 |
| CK302673.1 | Contig 51 | dinucleotide | AT | 14 | 7 |
| CK302673.1 | Contig 51 | dinucleotide | AT | 14 | 7 |
| CK302673.1 | Contig 51 | dinucleotide | AT | 14 | 7 |
| CK306857.1 | Contig 51 | dinucleotide | AT | 14 | 7 |
| CK306857.1 | Contig 51 | dinucleotide | AT | 14 | 7 |
| DV953111.1 | Contig 51 | dinucleotide | AT | 14 | 7 |
| CK307120.1 | Contig 52 | trinucleotide | AGC | 24 | 8 |
| CK310336.1 | Contig 52 | trinucleotide | AGC | 24 | 8 |
| CK312237.1 | Contig 52 | trinucleotide | AGC | 24 | 8 |
| CK235554.1 | Contig 53 | trinucleotide | CCG | 18 | 6 |
| CK307293.1 | Contig 53 | dinucleotide | AG | 14 | 7 |
| CK308241.1 | Contig 53 | dinucleotide | AG | 12 | 6 |
| CK308241.1 | Contig 53 | trinucleotide | CCG | 18 | 6 |
| DV579443.1 | Contig 53 | trinucleotide | CCG | 24 | 8 |
| DV956262.1 | Contig 53 | dinucleotide | AG | 12 | 6 |
| DV956262.1 | Contig 53 | trinucleotide | CCG | 18 | 6 |
| DV960511.1 | Contig 53 | dinucleotide | AG | 12 | 6 |
| CK307510.1 | Contig 54 | dinucleotide | AT | 20 | 10 |
| DV957683.1 | Contig 54 | dinucleotide | AT | 20 | 10 |
| CK307560.1 | Contig 55 | trinucleotide | AGG | 27 | 9 |
| CK308644.1 | Contig 55 | trinucleotide | AGG | 30 | 10 |
| CK314255.1 | Contig 55 | trinucleotide | AGG | 24 | 8 |
| DV581204.1 | Contig 55 | trinucleotide | AGG | 24 | 8 |
| CK307658.1 | Contig 56 | dinucleotide | AG | 30 | 15 |
| DV946876.1 | Contig 56 | dinucleotide | AG | 28 | 14 |
| CK307777.1 | Contig 57 | dinucleotide | AT | 14 | 7 |
| CK314696.1 | Contig 57 | dinucleotide | AT | 14 | 7 |
| CK307876.1 | Contig 58 | dinucleotide | AC | 14 | 7 |
| DV958648.1 | Contig 58 | dinucleotide | AC | 14 | 7 |
| CK308085.1 | Contig 59 | dinucleotide | AG | 16 | 8 |
| CK310002.1 | Contig 59 | dinucleotide | AG | 16 | 8 |
| CK302027.1 | Contig 60 | dinucleotide | AT | 14 | 7 |
| CK306722.1 | Contig 60 | dinucleotide | AT | 14 | 7 |
| CK308370.1 | Contig 60 | dinucleotide | AT | 14 | 7 |
| CK317358.1 | Contig 60 | dinucleotide | AT | 14 | 7 |
| DV958320.1 | Contig 60 | dinucleotide | AT | 14 | 7 |
| CK308379.1 | Contig 61 | trinucleotide | AGC | 18 | 6 |
| CK314447.1 | Contig 61 | trinucleotide | AGC | 18 | 6 |
| DV956792.1 | Contig 61 | trinucleotide | AAC | 27 | 9 |
| DV956792.1 | Contig 61 | trinucleotide | AGC | 18 | 6 |
| CK308584.1 | Contig 62 | dinucleotide | AT | 12 | 6 |
| DV949423.1 | Contig 62 | dinucleotide | AT | 12 | 6 |
| DV956642.1 | Contig 62 | dinucleotide | AT | 12 | 6 |
| CK308847.1 | Contig 63 | dinucleotide | AC | 14 | 7 |
| CK314290.1 | Contig 63 | dinucleotide | AC | 14 | 7 |
| DV947329.1 | Contig 63 | dinucleotide | AC | 14 | 7 |
| CK308973.1 | Contig 64 | dinucleotide | AT | 12 | 6 |
| CK309748.1 | Contig 64 | dinucleotide | AT | 12 | 6 |
| CK310033.1 | Contig 64 | dinucleotide | AT | 12 | 6 |
| DV951804.1 | Contig 64 | dinucleotide | AT | 12 | 6 |
| CK309002.1 | Contig 65 | dinucleotide | AC | 12 | 6 |
| CK315315.1 | Contig 65 | dinucleotide | AC | 16 | 8 |
| CK308139.1 | Contig 66 | dinucleotide | AG | 12 | 6 |
| CK308139.1 | Contig 66 | dinucleotide | AG | 16 | 8 |
| CK308139.1 | Contig 66 | dinucleotide | AG | 14 | 7 |
| CK309067.1 | Contig 66 | dinucleotide | AG | 16 | 8 |
| CK309067.1 | Contig 66 | dinucleotide | AG | 14 | 7 |
| CK309067.1 | Contig 66 | dinucleotide | AG | 12 | 6 |
| CK312186.1 | Contig 66 | dinucleotide | AG | 14 | 7 |
| CK312186.1 | Contig 66 | dinucleotide | AG | 12 | 6 |
| CK312186.1 | Contig 66 | dinucleotide | AG | 16 | 8 |
| CK309161.1 | Contig 67 | trinucleotide | AGC | 27 | 9 |
| DV953867.1 | Contig 67 | trinucleotide | AGC | 24 | 8 |
| CK309218.1 | Contig 68 | trinucleotide | AGG | 18 | 6 |
| CK309648.1 | Contig 68 | trinucleotide | AGG | 18 | 6 |
| CK311706.1 | Contig 68 | trinucleotide | AGG | 18 | 6 |
| CK313697.1 | Contig 68 | trinucleotide | AGG | 27 | 9 |
| CK314823.1 | Contig 68 | trinucleotide | AGG | 18 | 6 |
| DV952054.1 | Contig 68 | trinucleotide | AGG | 18 | 6 |
| DV956026.1 | Contig 68 | trinucleotide | AGG | 18 | 6 |
| DV958581.1 | Contig 68 | trinucleotide | AGG | 18 | 6 |
| DV959323.1 | Contig 68 | trinucleotide | AGG | 18 | 6 |
| CK309242.1 | Contig 69 | dinucleotide | AT | 14 | 7 |
| CK309433.1 | Contig 69 | dinucleotide | AT | 14 | 7 |
| CK309433.1 | Contig 69 | dinucleotide | AT | 14 | 7 |
| DV959690.1 | Contig 69 | dinucleotide | AT | 14 | 7 |
| CK304824.1 | Contig 70 | dinucleotide | AG | 14 | 7 |
| CK309418.1 | Contig 70 | dinucleotide | AG | 14 | 7 |
| CK309674.1 | Contig 70 | dinucleotide | AG | 14 | 7 |
| CK309476.1 | Contig 71 | trinucleotide | CCG | 18 | 6 |
| CK315583.1 | Contig 71 | trinucleotide | CCG | 18 | 6 |
| CK309696.1 | Contig 72 | dinucleotide | AT | 12 | 6 |
| DV956802.1 | Contig 72 | dinucleotide | AT | 16 | 8 |
| CK309830.1 | Contig 73 | trinucleotide | CCG | 21 | 7 |
| DV946414.1 | Contig 73 | trinucleotide | CCG | 27 | 9 |
| CK303069.1 | Contig 74 | dinucleotide | AT | 66 | 33 |
| CK310190.1 | Contig 74 | dinucleotide | AT | 68 | 34 |
| CK310202.1 | Contig 75 | dinucleotide | AC | 14 | 7 |
| CK310202.1 | Contig 75 | dinucleotide | AC | 12 | 6 |
| DV958885.1 | Contig 75 | dinucleotide | AC | 12 | 6 |
| CK310489.1 | Contig 76 | dinucleotide | AT | 14 | 7 |
| CK315559.1 | Contig 76 | dinucleotide | AT | 14 | 7 |
| CK310497.1 | Contig 77 | dinucleotide | AG | 22 | 11 |
| CK310497.1 | Contig 77 | trinucleotide | CCG | 27 | 9 |
| DV950587.1 | Contig 77 | trinucleotide | CCG | 27 | 9 |
| CK310610.1 | Contig 78 | dinucleotide | AT | 12 | 6 |
| DV959067.1 | Contig 78 | dinucleotide | AT | 12 | 6 |
| CK310774.1 | Contig 79 | dinucleotide | AT | 12 | 6 |
| CK310829.1 | Contig 79 | dinucleotide | AT | 12 | 6 |
| CK234772.1 | Contig 80 | dinucleotide | AT | 12 | 6 |
| CK303418.1 | Contig 80 | dinucleotide | AT | 18 | 9 |
| CK306978.1 | Contig 80 | dinucleotide | AT | 14 | 7 |
| CK311292.1 | Contig 80 | dinucleotide | AT | 14 | 7 |
| CK311752.1 | Contig 80 | dinucleotide | AT | 14 | 7 |
| CK317120.1 | Contig 80 | dinucleotide | AT | 18 | 9 |
| DV574838.1 | Contig 80 | dinucleotide | AT | 16 | 8 |
| DV574839.1 | Contig 80 | dinucleotide | AT | 16 | 8 |
| DV574840.1 | Contig 80 | dinucleotide | AT | 16 | 8 |
| DV574841.1 | Contig 80 | dinucleotide | AT | 16 | 8 |
| DV574845.1 | Contig 80 | dinucleotide | AT | 14 | 7 |
| DV574846.1 | Contig 80 | dinucleotide | AT | 14 | 7 |
| DV574847.1 | Contig 80 | dinucleotide | AT | 14 | 7 |
| DV574848.1 | Contig 80 | dinucleotide | AT | 14 | 7 |
| DV574855.1 | Contig 80 | dinucleotide | AT | 18 | 9 |
| DV574856.1 | Contig 80 | dinucleotide | AT | 18 | 9 |
| DV578557.1 | Contig 80 | dinucleotide | AT | 14 | 7 |
| DV578558.1 | Contig 80 | dinucleotide | AT | 14 | 7 |
| DV578559.1 | Contig 80 | dinucleotide | AT | 14 | 7 |
| DV578560.1 | Contig 80 | dinucleotide | AT | 14 | 7 |
| DV578561.1 | Contig 80 | dinucleotide | AT | 14 | 7 |
| DV578562.1 | Contig 80 | dinucleotide | AT | 14 | 7 |
| DV578563.1 | Contig 80 | dinucleotide | AT | 14 | 7 |
| DV578564.1 | Contig 80 | dinucleotide | AT | 14 | 7 |
| DV578565.1 | Contig 80 | dinucleotide | AT | 14 | 7 |
| DV578566.1 | Contig 80 | dinucleotide | AT | 14 | 7 |
| DV578567.1 | Contig 80 | dinucleotide | AT | 14 | 7 |
| DV578568.1 | Contig 80 | dinucleotide | AT | 14 | 7 |
| DV578569.1 | Contig 80 | dinucleotide | AT | 14 | 7 |
| DV578570.1 | Contig 80 | dinucleotide | AT | 14 | 7 |
| DV578571.1 | Contig 80 | dinucleotide | AT | 14 | 7 |
| DV578572.1 | Contig 80 | dinucleotide | AT | 14 | 7 |
| DV578573.1 | Contig 80 | dinucleotide | AT | 18 | 9 |
| DV578574.1 | Contig 80 | dinucleotide | AT | 18 | 9 |
| DV578575.1 | Contig 80 | dinucleotide | AT | 18 | 9 |
| DV578576.1 | Contig 80 | dinucleotide | AT | 18 | 9 |
| DV578577.1 | Contig 80 | dinucleotide | AT | 14 | 7 |
| DV578578.1 | Contig 80 | dinucleotide | AT | 14 | 7 |
| DV578579.1 | Contig 80 | dinucleotide | AT | 14 | 7 |
| DV578580.1 | Contig 80 | dinucleotide | AT | 14 | 7 |
| DV578581.1 | Contig 80 | dinucleotide | AT | 14 | 7 |
| DV578582.1 | Contig 80 | dinucleotide | AT | 14 | 7 |
| DV578583.1 | Contig 80 | dinucleotide | AT | 18 | 9 |
| DV578584.1 | Contig 80 | dinucleotide | AT | 18 | 9 |
| DV578586.1 | Contig 80 | dinucleotide | AT | 14 | 7 |
| DV578587.1 | Contig 80 | dinucleotide | AT | 14 | 7 |
| DV579605.1 | Contig 80 | dinucleotide | AT | 18 | 9 |
| DV579606.1 | Contig 80 | dinucleotide | AT | 18 | 9 |
| DV579607.1 | Contig 80 | dinucleotide | AT | 14 | 7 |
| DV582029.1 | Contig 80 | dinucleotide | AT | 20 | 10 |
| DV582030.1 | Contig 80 | dinucleotide | AT | 20 | 10 |
| DV582033.1 | Contig 80 | dinucleotide | AT | 18 | 9 |
| DV582034.1 | Contig 80 | dinucleotide | AT | 18 | 9 |
| DV582035.1 | Contig 80 | dinucleotide | AT | 20 | 10 |
| DV582036.1 | Contig 80 | dinucleotide | AT | 20 | 10 |
| DV582038.1 | Contig 80 | dinucleotide | AT | 20 | 10 |
| DV582039.1 | Contig 80 | dinucleotide | AT | 20 | 10 |
| DV582040.1 | Contig 80 | dinucleotide | AT | 20 | 10 |
| DV582041.1 | Contig 80 | dinucleotide | AT | 20 | 10 |
| DV582045.1 | Contig 80 | dinucleotide | AT | 18 | 9 |
| DV582046.1 | Contig 80 | dinucleotide | AT | 18 | 9 |
| DV583865.1 | Contig 80 | dinucleotide | AT | 20 | 10 |
| DV583866.1 | Contig 80 | dinucleotide | AT | 20 | 10 |
| DV954441.1 | Contig 80 | dinucleotide | AT | 18 | 9 |
| DV960676.1 | Contig 80 | dinucleotide | AT | 14 | 7 |
| CK311294.1 | Contig 81 | dinucleotide | AT | 12 | 6 |
| CK316313.1 | Contig 81 | dinucleotide | AT | 12 | 6 |
| CK311496.1 | Contig 82 | trinucleotide | AGC | 18 | 6 |
| CK313522.1 | Contig 82 | trinucleotide | AGC | 18 | 6 |
| CK303840.1 | Contig 83 | dinucleotide | AT | 12 | 6 |
| CK311655.1 | Contig 83 | dinucleotide | AT | 12 | 6 |
| CK311683.1 | Contig 83 | dinucleotide | AT | 12 | 6 |
| CK311905.1 | Contig 83 | dinucleotide | AC | 12 | 6 |
| CK311793.1 | Contig 84 | dinucleotide | AT | 16 | 8 |
| DV580018.1 | Contig 84 | dinucleotide | AT | 16 | 8 |
| DV580019.1 | Contig 84 | dinucleotide | AT | 16 | 8 |
| DV947499.1 | Contig 84 | dinucleotide | AT | 18 | 9 |
| DV952631.1 | Contig 84 | dinucleotide | AT | 16 | 8 |
| DV960761.1 | Contig 84 | dinucleotide | AT | 16 | 8 |
| CK311963.1 | Contig 85 | trinucleotide | CCG | 24 | 8 |
| DV573608.1 | Contig 85 | trinucleotide | CCG | 27 | 9 |
| CK312436.1 | Contig 86 | trinucleotide | AGG | 18 | 6 |
| DV946299.1 | Contig 86 | trinucleotide | AGG | 18 | 6 |
| DV952796.1 | Contig 86 | trinucleotide | AGG | 18 | 6 |
| DV961799.1 | Contig 86 | trinucleotide | AGG | 18 | 6 |
| DV961799.1 | Contig 86 | trinucleotide | AGG | 18 | 6 |
| CK312585.1 | Contig 87 | dinucleotide | AT | 56 | 28 |
| CK312585.1 | Contig 87 | dinucleotide | AT | 32 | 16 |
| CK312585.1 | Contig 87 | dinucleotide | AT | 18 | 9 |
| DV952760.1 | Contig 87 | dinucleotide | AT | 24 | 12 |
| DV952760.1 | Contig 87 | dinucleotide | AT | 28 | 14 |
| DV961886.1 | Contig 87 | dinucleotide | AT | 12 | 6 |
| DV961886.1 | Contig 87 | dinucleotide | AT | 16 | 8 |
| CK312773.1 | Contig 88 | dinucleotide | AT | 12 | 6 |
| DV573594.1 | Contig 88 | dinucleotide | AT | 12 | 6 |
| DV573595.1 | Contig 88 | dinucleotide | AT | 12 | 6 |
| DV573596.1 | Contig 88 | dinucleotide | AT | 12 | 6 |
| DV573597.1 | Contig 88 | dinucleotide | AT | 12 | 6 |
| DV577998.1 | Contig 88 | dinucleotide | AT | 12 | 6 |
| DV577999.1 | Contig 88 | dinucleotide | AT | 12 | 6 |
| DV578002.1 | Contig 88 | dinucleotide | AT | 12 | 6 |
| DV578003.1 | Contig 88 | dinucleotide | AT | 12 | 6 |
| DV579535.1 | Contig 88 | dinucleotide | AT | 12 | 6 |
| DV579536.1 | Contig 88 | dinucleotide | AT | 12 | 6 |
| DV581527.1 | Contig 88 | dinucleotide | AT | 12 | 6 |
| DV581528.1 | Contig 88 | dinucleotide | AT | 12 | 6 |
| DV581529.1 | Contig 88 | dinucleotide | AT | 12 | 6 |
| DV581530.1 | Contig 88 | dinucleotide | AT | 12 | 6 |
| DV581531.1 | Contig 88 | dinucleotide | AT | 12 | 6 |
| DV581532.1 | Contig 88 | dinucleotide | AT | 12 | 6 |
| DV949323.1 | Contig 88 | dinucleotide | AT | 12 | 6 |
| DV954333.1 | Contig 88 | dinucleotide | AT | 12 | 6 |
| CK312865.1 | Contig 89 | dinucleotide | AC | 12 | 6 |
| CK314508.1 | Contig 89 | dinucleotide | AC | 12 | 6 |
| CK312875.1 | Contig 90 | dinucleotide | AC | 18 | 9 |
| DV954234.1 | Contig 90 | dinucleotide | AC | 18 | 9 |
| CK312829.1 | Contig 91 | trinucleotide | AGG | 18 | 6 |
| CK312912.1 | Contig 91 | trinucleotide | AGG | 18 | 6 |
| DV948775.1 | Contig 91 | trinucleotide | AGG | 18 | 6 |
| DV950064.1 | Contig 91 | trinucleotide | AGG | 18 | 6 |
| DV951770.1 | Contig 91 | trinucleotide | AGG | 18 | 6 |
| DV952801.1 | Contig 91 | trinucleotide | AGG | 18 | 6 |
| DV954369.1 | Contig 91 | trinucleotide | AGG | 18 | 6 |
| DV954397.1 | Contig 91 | trinucleotide | AGG | 18 | 6 |
| DV961794.1 | Contig 91 | trinucleotide | AGG | 18 | 6 |
| CK313363.1 | Contig 92 | dinucleotide | AT | 14 | 7 |
| DV581631.1 | Contig 92 | dinucleotide | AT | 14 | 7 |
| DV581632.1 | Contig 92 | dinucleotide | AT | 14 | 7 |
| CK234258.1 | Contig 93 | dinucleotide | AT | 24 | 12 |
| CK313422.1 | Contig 93 | dinucleotide | AT | 24 | 12 |
| DV953620.1 | Contig 93 | dinucleotide | AT | 24 | 12 |
| CK312830.1 | Contig 94 | dinucleotide | AT | 14 | 7 |
| CK313432.1 | Contig 94 | dinucleotide | AT | 14 | 7 |
| DV946935.1 | Contig 94 | dinucleotide | AT | 14 | 7 |
| CK302382.1 | Contig 95 | dinucleotide | AC | 12 | 6 |
| CK306594.1 | Contig 95 | dinucleotide | AC | 12 | 6 |
| CK307576.1 | Contig 95 | dinucleotide | AC | 12 | 6 |
| CK313464.1 | Contig 95 | dinucleotide | AC | 12 | 6 |
| DV579673.1 | Contig 95 | dinucleotide | AC | 12 | 6 |
| DV579674.1 | Contig 95 | dinucleotide | AC | 12 | 6 |
| CK313484.1 | Contig 96 | trinucleotide | CCG | 27 | 9 |
| DV953187.1 | Contig 96 | trinucleotide | CCG | 21 | 7 |
| DV954499.1 | Contig 96 | trinucleotide | CCG | 21 | 7 |
| DV956214.1 | Contig 96 | trinucleotide | CCG | 27 | 9 |
| CK313634.1 | Contig 97 | dinucleotide | AT | 12 | 6 |
| DV945445.1 | Contig 97 | dinucleotide | AT | 12 | 6 |
| CK313798.1 | Contig 98 | trinucleotide | AAG | 18 | 6 |
| DV945566.1 | Contig 98 | trinucleotide | AAG | 21 | 7 |
| CK313835.1 | Contig 99 | trinucleotide | AAT | 18 | 6 |
| DV949645.1 | Contig 99 | trinucleotide | AAT | 18 | 6 |
| DV957623.1 | Contig 99 | trinucleotide | AAT | 18 | 6 |
| CK302901.1 | Contig 100 | trinucleotide | CCG | 18 | 6 |
| CK310767.1 | Contig 100 | trinucleotide | CCG | 18 | 6 |
| CK313897.1 | Contig 100 | trinucleotide | CCG | 18 | 6 |
| DV948265.1 | Contig 100 | trinucleotide | CCG | 18 | 6 |
| DV959166.1 | Contig 100 | trinucleotide | CCG | 18 | 6 |
| CK234612.1 | Contig 101 | dinucleotide | AC | 12 | 6 |
| CK234612.1 | Contig 101 | dinucleotide | AT | 14 | 7 |
| CK310468.1 | Contig 101 | dinucleotide | AT | 14 | 7 |
| CK312740.1 | Contig 101 | dinucleotide | AT | 12 | 6 |
| CK313961.1 | Contig 101 | dinucleotide | AT | 12 | 6 |
| CK314503.1 | Contig 101 | dinucleotide | AC | 12 | 6 |
| CK314503.1 | Contig 101 | dinucleotide | AT | 14 | 7 |
| CK316629.1 | Contig 101 | dinucleotide | AT | 14 | 7 |
| CK314156.1 | Contig 102 | dinucleotide | AT | 16 | 8 |
| CK314156.1 | Contig 102 | dinucleotide | AT | 16 | 8 |
| CK314156.1 | Contig 102 | dinucleotide | AT | 16 | 8 |
| CK315903.1 | Contig 102 | dinucleotide | AT | 14 | 7 |
| CK315903.1 | Contig 102 | dinucleotide | AT | 16 | 8 |
| CK315903.1 | Contig 102 | dinucleotide | AT | 16 | 8 |
| CK314330.1 | Contig 103 | trinucleotide | AGG | 18 | 6 |
| DV945709.1 | Contig 103 | trinucleotide | AGG | 18 | 6 |
| DV948054.1 | Contig 103 | trinucleotide | AGG | 18 | 6 |
| DV950496.1 | Contig 103 | trinucleotide | AGG | 18 | 6 |
| DV956007.1 | Contig 103 | trinucleotide | AGG | 18 | 6 |
| CK314565.1 | Contig 104 | dinucleotide | AT | 14 | 7 |
| DV945793.1 | Contig 104 | dinucleotide | AT | 16 | 8 |
| CK314744.1 | Contig 105 | trinucleotide | AGG | 21 | 7 |
| CK314744.1 | Contig 105 | trinucleotide | CCG | 21 | 7 |
| CK316913.1 | Contig 105 | trinucleotide | AGG | 21 | 7 |
| CK316913.1 | Contig 105 | trinucleotide | CCG | 27 | 9 |
| CK316983.1 | Contig 105 | trinucleotide | CCG | 30 | 10 |
| DV948398.1 | Contig 105 | trinucleotide | AGG | 21 | 7 |
| DV948398.1 | Contig 105 | trinucleotide | CCG | 24 | 8 |
| DV954132.1 | Contig 105 | trinucleotide | AGG | 21 | 7 |
| DV954132.1 | Contig 105 | trinucleotide | CCG | 33 | 11 |
| DV956368.1 | Contig 105 | trinucleotide | AGG | 21 | 7 |
| DV956368.1 | Contig 105 | trinucleotide | CCG | 21 | 7 |
| CK314749.1 | Contig 106 | dinucleotide | AT | 14 | 7 |
| DV577294.1 | Contig 106 | dinucleotide | AT | 16 | 8 |
| DV577295.1 | Contig 106 | dinucleotide | AT | 16 | 8 |
| DV577296.1 | Contig 106 | dinucleotide | AT | 16 | 8 |
| DV577297.1 | Contig 106 | dinucleotide | AT | 16 | 8 |
| DV577298.1 | Contig 106 | dinucleotide | AT | 22 | 11 |
| DV577299.1 | Contig 106 | dinucleotide | AT | 22 | 11 |
| CK315175.1 | Contig 107 | trinucleotide | AGG | 18 | 6 |
| DV946269.1 | Contig 107 | trinucleotide | AGG | 18 | 6 |
| CK311005.1 | Contig 108 | trinucleotide | AAT | 18 | 6 |
| CK315283.1 | Contig 108 | trinucleotide | AAT | 18 | 6 |
| CK316788.1 | Contig 108 | trinucleotide | AAT | 18 | 6 |
| CK234698.1 | Contig 109 | dinucleotide | AT | 12 | 6 |
| CK306709.1 | Contig 109 | dinucleotide | AT | 16 | 8 |
| CK306940.1 | Contig 109 | dinucleotide | AT | 12 | 6 |
| CK308410.1 | Contig 109 | dinucleotide | AT | 16 | 8 |
| CK315728.1 | Contig 109 | dinucleotide | AT | 16 | 8 |
| DV945879.1 | Contig 109 | dinucleotide | AT | 12 | 6 |
| DV946970.1 | Contig 109 | dinucleotide | AT | 12 | 6 |
| DV955634.1 | Contig 109 | dinucleotide | AT | 16 | 8 |
| DV959139.1 | Contig 109 | dinucleotide | AT | 16 | 8 |
| CK315752.1 | Contig 110 | trinucleotide | ATC | 21 | 7 |
| DV961222.1 | Contig 110 | trinucleotide | ATC | 24 | 8 |
| CK315765.1 | Contig 111 | dinucleotide | AC | 14 | 7 |
| DV956445.1 | Contig 111 | dinucleotide | AC | 14 | 7 |
| CK308306.1 | Contig 112 | dinucleotide | AT | 12 | 6 |
| CK308811.1 | Contig 112 | dinucleotide | AT | 14 | 7 |
| CK310397.1 | Contig 112 | dinucleotide | AT | 12 | 6 |
| CK310526.1 | Contig 112 | dinucleotide | AT | 12 | 6 |
| CK315875.1 | Contig 112 | dinucleotide | AT | 12 | 6 |
| CK316914.1 | Contig 112 | dinucleotide | AT | 12 | 6 |
| DV578956.1 | Contig 112 | dinucleotide | AT | 12 | 6 |
| DV578957.1 | Contig 112 | dinucleotide | AT | 12 | 6 |
| DV578958.1 | Contig 112 | dinucleotide | AT | 12 | 6 |
| DV578959.1 | Contig 112 | dinucleotide | AT | 12 | 6 |
| DV578962.1 | Contig 112 | dinucleotide | AT | 12 | 6 |
| DV578963.1 | Contig 112 | dinucleotide | AT | 12 | 6 |
| DV578964.1 | Contig 112 | dinucleotide | AT | 12 | 6 |
| DV578965.1 | Contig 112 | dinucleotide | AT | 12 | 6 |
| DV578966.1 | Contig 112 | dinucleotide | AT | 12 | 6 |
| DV578967.1 | Contig 112 | dinucleotide | AT | 12 | 6 |
| DV584040.1 | Contig 112 | dinucleotide | AT | 12 | 6 |
| DV584041.1 | Contig 112 | dinucleotide | AT | 12 | 6 |
| DV584044.1 | Contig 112 | dinucleotide | AT | 12 | 6 |
| DV584045.1 | Contig 112 | dinucleotide | AT | 12 | 6 |
| CK316418.1 | Contig 113 | dinucleotide | AG | 14 | 7 |
| DV954425.1 | Contig 113 | dinucleotide | AG | 14 | 7 |
| CK304587.1 | Contig 114 | trinucleotide | CCG | 18 | 6 |
| CK316444.1 | Contig 114 | trinucleotide | CCG | 18 | 6 |
| DV952792.1 | Contig 114 | trinucleotide | CCG | 30 | 10 |
| CK316597.1 | Contig 115 | dinucleotide | AT | 14 | 7 |
| DV947108.1 | Contig 115 | dinucleotide | AT | 14 | 7 |
| CK316939.1 | Contig 116 | trinucleotide | AAG | 18 | 6 |
| CK316939.1 | Contig 116 | trinucleotide | AGG | 27 | 9 |
| CK316939.1 | Contig 116 | trinucleotide | CCG | 21 | 7 |
| DV947912.1 | Contig 116 | trinucleotide | AAG | 18 | 6 |
| DV947912.1 | Contig 116 | trinucleotide | AGG | 24 | 8 |
| DV947912.1 | Contig 116 | trinucleotide | CCG | 18 | 6 |
| CK316970.1 | Contig 117 | dinucleotide | AC | 12 | 6 |
| DV956403.1 | Contig 117 | dinucleotide | AC | 12 | 6 |
| CK317294.1 | Contig 118 | trinucleotide | ACC | 21 | 7 |
| DV952137.1 | Contig 118 | trinucleotide | ACC | 18 | 6 |
| DV953687.1 | Contig 118 | trinucleotide | ACC | 21 | 7 |
| DV571030.1 | Contig 119 | dinucleotide | AG | 12 | 6 |
| DV571031.1 | Contig 119 | dinucleotide | AG | 12 | 6 |
| DV571047.1 | Contig 120 | dinucleotide | AT | 14 | 7 |
| DV571048.1 | Contig 120 | dinucleotide | AT | 14 | 7 |
| CK235321.1 | Contig 121 | dinucleotide | AT | 14 | 7 |
| CK305134.1 | Contig 121 | dinucleotide | AT | 12 | 6 |
| CK312659.1 | Contig 121 | dinucleotide | AT | 12 | 6 |
| DV571499.1 | Contig 121 | dinucleotide | AT | 12 | 6 |
| DV958762.1 | Contig 121 | dinucleotide | AT | 12 | 6 |
| DV571625.1 | Contig 122 | dinucleotide | AC | 14 | 7 |
| DV571625.1 | Contig 122 | dinucleotide | AT | 26 | 13 |
| DV571626.1 | Contig 122 | dinucleotide | AC | 14 | 7 |
| DV571626.1 | Contig 122 | dinucleotide | AT | 26 | 13 |
| DV948000.1 | Contig 122 | dinucleotide | AC | 14 | 7 |
| CK235552.1 | Contig 123 | dinucleotide | AC | 14 | 7 |
| CK303063.1 | Contig 123 | dinucleotide | AC | 14 | 7 |
| CK303247.1 | Contig 123 | dinucleotide | AC | 16 | 8 |
| CK303917.1 | Contig 123 | dinucleotide | AC | 14 | 7 |
| DV572132.1 | Contig 123 | dinucleotide | AC | 12 | 6 |
| DV572133.1 | Contig 123 | dinucleotide | AC | 12 | 6 |
| DV572398.1 | Contig 124 | dinucleotide | AT | 12 | 6 |
| DV572399.1 | Contig 124 | dinucleotide | AT | 12 | 6 |
| DV577325.1 | Contig 124 | dinucleotide | AC | 14 | 7 |
| DV577325.1 | Contig 124 | dinucleotide | AT | 12 | 6 |
| DV577326.1 | Contig 124 | dinucleotide | AC | 14 | 7 |
| DV577326.1 | Contig 124 | dinucleotide | AT | 12 | 6 |
| DV572482.1 | Contig 125 | trinucleotide | AGC | 27 | 9 |
| DV572483.1 | Contig 125 | trinucleotide | AGC | 27 | 9 |
| DV954144.1 | Contig 125 | trinucleotide | AGC | 54 | 18 |
| DV572615.1 | Contig 126 | trinucleotide | ACC | 21 | 7 |
| DV572616.1 | Contig 126 | trinucleotide | ACC | 21 | 7 |
| DV572617.1 | Contig 126 | trinucleotide | ACC | 21 | 7 |
| DV572618.1 | Contig 126 | trinucleotide | ACC | 21 | 7 |
| DV572619.1 | Contig 126 | trinucleotide | ACC | 21 | 7 |
| DV572620.1 | Contig 126 | trinucleotide | ACC | 21 | 7 |
| DV572621.1 | Contig 126 | trinucleotide | ACC | 21 | 7 |
| DV572622.1 | Contig 126 | trinucleotide | ACC | 21 | 7 |
| DV572623.1 | Contig 126 | trinucleotide | ACC | 21 | 7 |
| DV572624.1 | Contig 126 | trinucleotide | ACC | 21 | 7 |
| DV572625.1 | Contig 126 | trinucleotide | ACC | 21 | 7 |
| DV572626.1 | Contig 126 | trinucleotide | ACC | 18 | 6 |
| DV572627.1 | Contig 126 | trinucleotide | ACC | 18 | 6 |
| DV572628.1 | Contig 126 | trinucleotide | ACC | 18 | 6 |
| DV572629.1 | Contig 126 | trinucleotide | ACC | 18 | 6 |
| DV572630.1 | Contig 126 | trinucleotide | ACC | 18 | 6 |
| DV572631.1 | Contig 126 | trinucleotide | ACC | 18 | 6 |
| DV572829.1 | Contig 127 | trinucleotide | AGG | 18 | 6 |
| DV572830.1 | Contig 127 | trinucleotide | AGG | 18 | 6 |
| DV572903.1 | Contig 128 | dinucleotide | AC | 12 | 6 |
| DV572904.1 | Contig 128 | dinucleotide | AC | 12 | 6 |
| CK304090.1 | Contig 129 | trinucleotide | AAT | 18 | 6 |
| CK304845.1 | Contig 129 | trinucleotide | AAT | 18 | 6 |
| DV573340.1 | Contig 129 | trinucleotide | AAT | 18 | 6 |
| DV573341.1 | Contig 129 | trinucleotide | AAT | 18 | 6 |
| DV573442.1 | Contig 130 | dinucleotide | AC | 12 | 6 |
| DV573443.1 | Contig 130 | dinucleotide | AC | 12 | 6 |
| DV573444.1 | Contig 130 | dinucleotide | AC | 12 | 6 |
| DV573445.1 | Contig 130 | dinucleotide | AC | 12 | 6 |
| DV573446.1 | Contig 130 | dinucleotide | AC | 12 | 6 |
| DV573447.1 | Contig 130 | dinucleotide | AC | 12 | 6 |
| DV573448.1 | Contig 130 | dinucleotide | AC | 12 | 6 |
| DV573561.1 | Contig 131 | dinucleotide | AT | 16 | 8 |
| DV573562.1 | Contig 131 | dinucleotide | AT | 16 | 8 |
| DV573569.1 | Contig 132 | dinucleotide | AT | 12 | 6 |
| DV573570.1 | Contig 132 | dinucleotide | AT | 12 | 6 |
| CK234560.1 | Contig 133 | dinucleotide | AT | 12 | 6 |
| CK235864.1 | Contig 133 | dinucleotide | AT | 12 | 6 |
| CK315532.1 | Contig 133 | dinucleotide | AT | 12 | 6 |
| DV573662.1 | Contig 133 | dinucleotide | AT | 12 | 6 |
| DV573663.1 | Contig 133 | dinucleotide | AT | 12 | 6 |
| DV573666.1 | Contig 133 | dinucleotide | AT | 12 | 6 |
| DV573667.1 | Contig 133 | dinucleotide | AT | 12 | 6 |
| DV573668.1 | Contig 133 | dinucleotide | AT | 12 | 6 |
| DV573670.1 | Contig 133 | dinucleotide | AT | 12 | 6 |
| DV573671.1 | Contig 133 | dinucleotide | AT | 12 | 6 |
| DV581575.1 | Contig 133 | dinucleotide | AT | 12 | 6 |
| DV581576.1 | Contig 133 | dinucleotide | AT | 12 | 6 |
| DV573706.1 | Contig 134 | trinucleotide | AAC | 21 | 7 |
| DV573706.1 | Contig 134 | dinucleotide | AC | 12 | 6 |
| DV573707.1 | Contig 134 | trinucleotide | AAC | 21 | 7 |
| DV573707.1 | Contig 134 | dinucleotide | AC | 12 | 6 |
| DV579544.1 | Contig 134 | trinucleotide | AAC | 21 | 7 |
| DV579544.1 | Contig 134 | dinucleotide | AC | 12 | 6 |
| DV579545.1 | Contig 134 | trinucleotide | AAC | 21 | 7 |
| DV579545.1 | Contig 134 | dinucleotide | AC | 12 | 6 |
| DV573872.1 | Contig 135 | dinucleotide | AG | 14 | 7 |
| DV573873.1 | Contig 135 | dinucleotide | AG | 14 | 7 |
| CK314682.1 | Contig 136 | dinucleotide | AT | 12 | 6 |
| DV574289.1 | Contig 136 | dinucleotide | AT | 12 | 6 |
| DV574290.1 | Contig 136 | dinucleotide | AT | 12 | 6 |
| DV574303.1 | Contig 136 | dinucleotide | AT | 12 | 6 |
| DV574304.1 | Contig 136 | dinucleotide | AT | 12 | 6 |
| DV579563.1 | Contig 136 | dinucleotide | AT | 12 | 6 |
| DV579564.1 | Contig 136 | dinucleotide | AT | 12 | 6 |
| DV574330.1 | Contig 137 | dinucleotide | AG | 18 | 9 |
| DV574331.1 | Contig 137 | dinucleotide | AG | 18 | 9 |
| DV574332.1 | Contig 137 | dinucleotide | AG | 18 | 9 |
| DV574333.1 | Contig 137 | dinucleotide | AG | 18 | 9 |
| DV574334.1 | Contig 137 | dinucleotide | AG | 14 | 7 |
| DV574335.1 | Contig 137 | dinucleotide | AG | 14 | 7 |
| DV574336.1 | Contig 137 | dinucleotide | AG | 14 | 7 |
| DV574337.1 | Contig 137 | dinucleotide | AG | 14 | 7 |
| DV574859.1 | Contig 138 | trinucleotide | AGC | 18 | 6 |
| DV574860.1 | Contig 138 | trinucleotide | AGC | 18 | 6 |
| DV574861.1 | Contig 138 | trinucleotide | AGC | 18 | 6 |
| DV574862.1 | Contig 138 | trinucleotide | AGC | 18 | 6 |
| DV574863.1 | Contig 138 | trinucleotide | AGC | 18 | 6 |
| DV574864.1 | Contig 138 | trinucleotide | AGC | 18 | 6 |
| DV578590.1 | Contig 138 | trinucleotide | AGC | 18 | 6 |
| DV578591.1 | Contig 138 | trinucleotide | AGC | 18 | 6 |
| DV578592.1 | Contig 138 | trinucleotide | AGC | 18 | 6 |
| DV578593.1 | Contig 138 | trinucleotide | AGC | 18 | 6 |
| DV582059.1 | Contig 138 | trinucleotide | AGC | 18 | 6 |
| DV582060.1 | Contig 138 | trinucleotide | AGC | 18 | 6 |
| DV583873.1 | Contig 138 | trinucleotide | AGC | 18 | 6 |
| DV583874.1 | Contig 138 | trinucleotide | AGC | 18 | 6 |
| CK302880.1 | Contig 139 | dinucleotide | AT | 22 | 11 |
| CK306952.1 | Contig 139 | dinucleotide | AT | 24 | 12 |
| CK315080.1 | Contig 139 | dinucleotide | AT | 20 | 10 |
| CK315372.1 | Contig 139 | dinucleotide | AT | 20 | 10 |
| CK317119.1 | Contig 139 | dinucleotide | AT | 20 | 10 |
| DV575290.1 | Contig 139 | dinucleotide | AT | 22 | 11 |
| DV575291.1 | Contig 139 | dinucleotide | AT | 22 | 11 |
| DV575294.1 | Contig 139 | dinucleotide | AT | 22 | 11 |
| DV575295.1 | Contig 139 | dinucleotide | AT | 22 | 11 |
| DV575296.1 | Contig 139 | dinucleotide | AT | 22 | 11 |
| DV575297.1 | Contig 139 | dinucleotide | AT | 22 | 11 |
| DV575298.1 | Contig 139 | dinucleotide | AT | 24 | 12 |
| DV575299.1 | Contig 139 | dinucleotide | AT | 24 | 12 |
| DV576180.1 | Contig 139 | dinucleotide | AT | 18 | 9 |
| DV576181.1 | Contig 139 | dinucleotide | AT | 18 | 9 |
| DV578933.1 | Contig 139 | dinucleotide | AT | 24 | 12 |
| DV578934.1 | Contig 139 | dinucleotide | AT | 24 | 12 |
| DV578938.1 | Contig 139 | dinucleotide | AT | 24 | 12 |
| DV578939.1 | Contig 139 | dinucleotide | AT | 24 | 12 |
| DV578940.1 | Contig 139 | dinucleotide | AT | 24 | 12 |
| DV578941.1 | Contig 139 | dinucleotide | AT | 24 | 12 |
| DV578944.1 | Contig 139 | dinucleotide | AT | 24 | 12 |
| DV578945.1 | Contig 139 | dinucleotide | AT | 24 | 12 |
| DV578948.1 | Contig 139 | dinucleotide | AT | 22 | 11 |
| DV578949.1 | Contig 139 | dinucleotide | AT | 22 | 11 |
| DV578950.1 | Contig 139 | dinucleotide | AT | 22 | 11 |
| DV578951.1 | Contig 139 | dinucleotide | AT | 22 | 11 |
| DV582220.1 | Contig 139 | dinucleotide | AT | 22 | 11 |
| DV582221.1 | Contig 139 | dinucleotide | AT | 22 | 11 |
| DV950713.1 | Contig 139 | dinucleotide | AT | 24 | 12 |
| DV953919.1 | Contig 139 | dinucleotide | AT | 22 | 11 |
| DV575451.1 | Contig 140 | dinucleotide | AC | 12 | 6 |
| DV575452.1 | Contig 140 | dinucleotide | AC | 12 | 6 |
| DV575453.1 | Contig 140 | dinucleotide | AC | 12 | 6 |
| DV575454.1 | Contig 140 | dinucleotide | AC | 12 | 6 |
| DV575455.1 | Contig 140 | dinucleotide | AC | 12 | 6 |
| DV575456.1 | Contig 140 | dinucleotide | AC | 12 | 6 |
| DV575459.1 | Contig 140 | dinucleotide | AC | 12 | 6 |
| DV575460.1 | Contig 140 | dinucleotide | AC | 12 | 6 |
| DV575461.1 | Contig 140 | dinucleotide | AC | 12 | 6 |
| DV575462.1 | Contig 140 | dinucleotide | AC | 12 | 6 |
| DV579005.1 | Contig 140 | dinucleotide | AC | 12 | 6 |
| DV579006.1 | Contig 140 | dinucleotide | AC | 12 | 6 |
| DV575511.1 | Contig 141 | trinucleotide | AAG | 21 | 7 |
| DV575512.1 | Contig 141 | trinucleotide | AAG | 21 | 7 |
| DV582292.1 | Contig 141 | trinucleotide | AAG | 21 | 7 |
| DV582293.1 | Contig 141 | trinucleotide | AAG | 21 | 7 |
| DV954364.1 | Contig 141 | trinucleotide | AAG | 21 | 7 |
| DV954573.1 | Contig 141 | trinucleotide | AAG | 21 | 7 |
| DV576233.1 | Contig 142 | dinucleotide | AT | 14 | 7 |
| DV576234.1 | Contig 142 | dinucleotide | AT | 14 | 7 |
| CK234451.1 | Contig 143 | dinucleotide | AG | 14 | 7 |
| DV576279.1 | Contig 143 | dinucleotide | AG | 14 | 7 |
| DV576280.1 | Contig 143 | dinucleotide | AG | 14 | 7 |
| DV576507.1 | Contig 144 | dinucleotide | AC | 16 | 8 |
| DV576508.1 | Contig 144 | dinucleotide | AC | 16 | 8 |
| DV576541.1 | Contig 145 | dinucleotide | AC | 18 | 9 |
| DV576542.1 | Contig 145 | dinucleotide | AC | 18 | 9 |
| DV576618.1 | Contig 146 | dinucleotide | AG | 12 | 6 |
| DV576619.1 | Contig 146 | dinucleotide | AG | 12 | 6 |
| DV576690.1 | Contig 147 | dinucleotide | AC | 14 | 7 |
| DV576690.1 | Contig 147 | dinucleotide | AC | 16 | 8 |
| DV576691.1 | Contig 147 | dinucleotide | AC | 16 | 8 |
| DV576691.1 | Contig 147 | dinucleotide | AC | 14 | 7 |
| DV576694.1 | Contig 148 | dinucleotide | AT | 16 | 8 |
| DV576695.1 | Contig 148 | dinucleotide | AT | 16 | 8 |
| DV950581.1 | Contig 148 | dinucleotide | AT | 16 | 8 |
| DV576762.1 | Contig 149 | dinucleotide | AG | 14 | 7 |
| DV576763.1 | Contig 149 | dinucleotide | AG | 14 | 7 |
| DV576805.1 | Contig 150 | dinucleotide | AC | 14 | 7 |
| DV576806.1 | Contig 150 | dinucleotide | AC | 14 | 7 |
| DV572281.1 | Contig 151 | trinucleotide | AGC | 18 | 6 |
| DV572282.1 | Contig 151 | trinucleotide | AGC | 18 | 6 |
| DV577249.1 | Contig 151 | trinucleotide | AGC | 18 | 6 |
| DV577250.1 | Contig 151 | trinucleotide | AGC | 18 | 6 |
| DV961400.1 | Contig 151 | trinucleotide | AGC | 18 | 6 |
| DV577363.1 | Contig 152 | trinucleotide | AAT | 33 | 11 |
| DV952014.1 | Contig 152 | trinucleotide | AAT | 21 | 7 |
| DV577617.1 | Contig 153 | dinucleotide | AG | 14 | 7 |
| DV577618.1 | Contig 153 | dinucleotide | AG | 14 | 7 |
| DV577903.1 | Contig 154 | dinucleotide | AT | 16 | 8 |
| DV577904.1 | Contig 154 | dinucleotide | AT | 16 | 8 |
| DV961283.1 | Contig 154 | dinucleotide | AT | 16 | 8 |
| DV961283.1 | Contig 154 | dinucleotide | AT | 12 | 6 |
| DV578015.1 | Contig 155 | dinucleotide | AC | 30 | 15 |
| DV578016.1 | Contig 155 | dinucleotide | AC | 30 | 15 |
| DV578017.1 | Contig 155 | dinucleotide | AC | 26 | 13 |
| DV578018.1 | Contig 155 | dinucleotide | AC | 26 | 13 |
| CK301847.1 | Contig 156 | trinucleotide | ATC | 18 | 6 |
| DV573660.1 | Contig 156 | trinucleotide | ATC | 18 | 6 |
| DV573661.1 | Contig 156 | trinucleotide | ATC | 18 | 6 |
| DV578031.1 | Contig 156 | trinucleotide | ATC | 18 | 6 |
| DV578032.1 | Contig 156 | trinucleotide | ATC | 18 | 6 |
| DV581571.1 | Contig 156 | trinucleotide | ATC | 18 | 6 |
| DV581572.1 | Contig 156 | trinucleotide | ATC | 18 | 6 |
| DV945042.1 | Contig 156 | trinucleotide | ATC | 18 | 6 |
| DV946460.1 | Contig 156 | trinucleotide | ATC | 18 | 6 |
| DV951022.1 | Contig 156 | trinucleotide | ATC | 18 | 6 |
| DV951161.1 | Contig 156 | trinucleotide | ATC | 18 | 6 |
| DV951724.1 | Contig 156 | trinucleotide | ATC | 18 | 6 |
| DV952814.1 | Contig 156 | trinucleotide | ATC | 18 | 6 |
| DV953668.1 | Contig 156 | trinucleotide | ATC | 18 | 6 |
| DV953998.1 | Contig 156 | trinucleotide | ATC | 18 | 6 |
| DV955363.1 | Contig 156 | trinucleotide | ATC | 18 | 6 |
| DV955421.1 | Contig 156 | trinucleotide | ATC | 18 | 6 |
| DV961345.1 | Contig 156 | trinucleotide | ATC | 18 | 6 |
| CK310590.1 | Contig 157 | dinucleotide | AC | 48 | 24 |
| DV578549.1 | Contig 157 | dinucleotide | AC | 18 | 9 |
| DV578549.1 | Contig 157 | dinucleotide | AC | 26 | 13 |
| DV578550.1 | Contig 157 | dinucleotide | AC | 18 | 9 |
| DV578550.1 | Contig 157 | dinucleotide | AC | 26 | 13 |
| DV960712.1 | Contig 157 | dinucleotide | AC | 22 | 11 |
| CK304731.1 | Contig 158 | dinucleotide | AT | 12 | 6 |
| CK304731.1 | Contig 158 | dinucleotide | AT | 18 | 9 |
| DV578839.1 | Contig 158 | dinucleotide | AT | 12 | 6 |
| DV578840.1 | Contig 158 | dinucleotide | AT | 12 | 6 |
| DV949782.1 | Contig 158 | dinucleotide | AT | 18 | 9 |
| DV949782.1 | Contig 158 | dinucleotide | AT | 12 | 6 |
| DV955329.1 | Contig 158 | dinucleotide | AT | 14 | 7 |
| DV579042.1 | Contig 159 | trinucleotide | AGC | 18 | 6 |
| DV579043.1 | Contig 159 | trinucleotide | AGC | 18 | 6 |
| CK303871.1 | Contig 160 | dinucleotide | AC | 16 | 8 |
| CK303871.1 | Contig 160 | dinucleotide | AC | 16 | 8 |
| DV575558.1 | Contig 160 | dinucleotide | AC | 30 | 15 |
| DV575559.1 | Contig 160 | dinucleotide | AC | 30 | 15 |
| DV579086.1 | Contig 160 | dinucleotide | AC | 40 | 20 |
| DV579087.1 | Contig 160 | dinucleotide | AC | 40 | 20 |
| DV579088.1 | Contig 160 | dinucleotide | AC | 38 | 19 |
| DV579089.1 | Contig 160 | dinucleotide | AC | 38 | 19 |
| CK310240.1 | Contig 161 | dinucleotide | AC | 14 | 7 |
| CK310240.1 | Contig 161 | dinucleotide | AC | 14 | 7 |
| CK315109.1 | Contig 161 | dinucleotide | AC | 14 | 7 |
| DV575826.1 | Contig 161 | dinucleotide | AC | 12 | 6 |
| DV575827.1 | Contig 161 | dinucleotide | AC | 12 | 6 |
| DV579180.1 | Contig 161 | dinucleotide | AC | 12 | 6 |
| DV579181.1 | Contig 161 | dinucleotide | AC | 12 | 6 |
| DV579182.1 | Contig 161 | dinucleotide | AC | 14 | 7 |
| DV579183.1 | Contig 161 | dinucleotide | AC | 14 | 7 |
| DV579184.1 | Contig 161 | dinucleotide | AC | 12 | 6 |
| DV579185.1 | Contig 161 | dinucleotide | AC | 12 | 6 |
| DV579186.1 | Contig 161 | dinucleotide | AC | 12 | 6 |
| DV579187.1 | Contig 161 | dinucleotide | AC | 12 | 6 |
| DV579188.1 | Contig 161 | dinucleotide | AC | 12 | 6 |
| DV579650.1 | Contig 161 | dinucleotide | AC | 14 | 7 |
| DV579651.1 | Contig 161 | dinucleotide | AC | 14 | 7 |
| DV582386.1 | Contig 161 | dinucleotide | AC | 12 | 6 |
| DV582387.1 | Contig 161 | dinucleotide | AC | 12 | 6 |
| DV946886.1 | Contig 161 | dinucleotide | AC | 14 | 7 |
| DV947949.1 | Contig 161 | dinucleotide | AC | 12 | 6 |
| DV947949.1 | Contig 161 | dinucleotide | AC | 14 | 7 |
| DV958200.1 | Contig 161 | dinucleotide | AC | 12 | 6 |
| DV579347.1 | Contig 162 | dinucleotide | AC | 30 | 15 |
| DV579348.1 | Contig 162 | dinucleotide | AC | 30 | 15 |
| DV579359.1 | Contig 163 | trinucleotide | CCG | 21 | 7 |
| DV579360.1 | Contig 163 | trinucleotide | CCG | 21 | 7 |
| CK306731.1 | Contig 164 | dinucleotide | AC | 12 | 6 |
| CK312176.1 | Contig 164 | dinucleotide | AT | 14 | 7 |
| CK313815.1 | Contig 164 | dinucleotide | AT | 18 | 9 |
| DV573136.1 | Contig 164 | dinucleotide | AT | 20 | 10 |
| DV573137.1 | Contig 164 | dinucleotide | AT | 20 | 10 |
| DV577767.1 | Contig 164 | dinucleotide | AT | 18 | 9 |
| DV577768.1 | Contig 164 | dinucleotide | AT | 18 | 9 |
| DV577769.1 | Contig 164 | dinucleotide | AT | 18 | 9 |
| DV577770.1 | Contig 164 | dinucleotide | AT | 18 | 9 |
| DV579507.1 | Contig 164 | dinucleotide | AT | 20 | 10 |
| DV579508.1 | Contig 164 | dinucleotide | AT | 20 | 10 |
| DV581212.1 | Contig 164 | dinucleotide | AT | 18 | 9 |
| DV581213.1 | Contig 164 | dinucleotide | AT | 18 | 9 |
| DV581214.1 | Contig 164 | dinucleotide | AT | 20 | 10 |
| DV581215.1 | Contig 164 | dinucleotide | AT | 20 | 10 |
| DV581216.1 | Contig 164 | dinucleotide | AT | 20 | 10 |
| DV952192.1 | Contig 164 | dinucleotide | AC | 12 | 6 |
| DV960643.1 | Contig 164 | dinucleotide | AT | 18 | 9 |
| DV580054.1 | Contig 165 | trinucleotide | AGG | 21 | 7 |
| DV580055.1 | Contig 165 | trinucleotide | AGG | 21 | 7 |
| DV945077.1 | Contig 165 | trinucleotide | AGG | 24 | 8 |
| DV580292.1 | Contig 166 | dinucleotide | AT | 18 | 9 |
| DV580293.1 | Contig 166 | dinucleotide | AT | 18 | 9 |
| DV580316.1 | Contig 167 | trinucleotide | AGC | 18 | 6 |
| DV957385.1 | Contig 167 | trinucleotide | AGC | 18 | 6 |
| DV580366.1 | Contig 168 | dinucleotide | AT | 14 | 7 |
| DV580367.1 | Contig 168 | dinucleotide | AT | 14 | 7 |
| CK308029.1 | Contig 169 | trinucleotide | AAT | 18 | 6 |
| CK315257.1 | Contig 169 | trinucleotide | AAT | 18 | 6 |
| DV580602.1 | Contig 169 | trinucleotide | AAT | 21 | 7 |
| DV580603.1 | Contig 169 | trinucleotide | AAT | 21 | 7 |
| DV580604.1 | Contig 169 | trinucleotide | AAT | 18 | 6 |
| DV580605.1 | Contig 169 | trinucleotide | AAT | 18 | 6 |
| DV583067.1 | Contig 169 | trinucleotide | AAT | 18 | 6 |
| DV583068.1 | Contig 169 | trinucleotide | AAT | 18 | 6 |
| DV955751.1 | Contig 169 | trinucleotide | AAT | 18 | 6 |
| DV580669.1 | Contig 170 | dinucleotide | AT | 16 | 8 |
| DV580670.1 | Contig 170 | dinucleotide | AT | 16 | 8 |
| CK309944.1 | Contig 171 | dinucleotide | AT | 16 | 8 |
| DV580854.1 | Contig 171 | dinucleotide | AT | 16 | 8 |
| DV580855.1 | Contig 171 | dinucleotide | AT | 16 | 8 |
| DV580905.1 | Contig 172 | dinucleotide | AT | 14 | 7 |
| DV580906.1 | Contig 172 | dinucleotide | AT | 14 | 7 |
| DV580907.1 | Contig 172 | dinucleotide | AT | 14 | 7 |
| DV580908.1 | Contig 172 | dinucleotide | AT | 14 | 7 |
| DV955571.1 | Contig 172 | dinucleotide | AT | 14 | 7 |
| CK305918.1 | Contig 173 | dinucleotide | AT | 14 | 7 |
| DV577605.1 | Contig 173 | dinucleotide | AT | 14 | 7 |
| DV580936.1 | Contig 173 | dinucleotide | AT | 14 | 7 |
| DV580937.1 | Contig 173 | dinucleotide | AT | 14 | 7 |
| DV580938.1 | Contig 173 | dinucleotide | AT | 14 | 7 |
| DV580939.1 | Contig 173 | dinucleotide | AT | 14 | 7 |
| DV580940.1 | Contig 173 | dinucleotide | AT | 14 | 7 |
| DV580941.1 | Contig 173 | dinucleotide | AT | 14 | 7 |
| DV581064.1 | Contig 174 | dinucleotide | AT | 12 | 6 |
| DV581065.1 | Contig 174 | dinucleotide | AT | 12 | 6 |
| DV583357.1 | Contig 174 | dinucleotide | AT | 12 | 6 |
| DV581985.1 | Contig 175 | dinucleotide | AC | 12 | 6 |
| DV581986.1 | Contig 175 | dinucleotide | AC | 12 | 6 |
| DV582571.1 | Contig 176 | dinucleotide | AT | 16 | 8 |
| DV582572.1 | Contig 176 | dinucleotide | AT | 16 | 8 |
| DV582684.1 | Contig 177 | trinucleotide | AGG | 21 | 7 |
| DV582685.1 | Contig 177 | trinucleotide | AGG | 21 | 7 |
| DV582742.1 | Contig 178 | trinucleotide | AAT | 27 | 9 |
| DV582743.1 | Contig 178 | trinucleotide | AAT | 27 | 9 |
| CK305413.1 | Contig 179 | dinucleotide | AT | 16 | 8 |
| CK305413.1 | Contig 179 | dinucleotide | AT | 26 | 13 |
| CK306085.1 | Contig 179 | dinucleotide | AT | 28 | 14 |
| CK306085.1 | Contig 179 | dinucleotide | AT | 16 | 8 |
| CK312456.1 | Contig 179 | dinucleotide | AT | 16 | 8 |
| CK312456.1 | Contig 179 | dinucleotide | AT | 22 | 11 |
| CK315017.1 | Contig 179 | dinucleotide | AT | 34 | 17 |
| DV945088.1 | Contig 179 | dinucleotide | AT | 28 | 14 |
| DV950192.1 | Contig 179 | dinucleotide | AT | 28 | 14 |
| DV955875.1 | Contig 179 | dinucleotide | AT | 28 | 14 |
| DV958344.1 | Contig 179 | dinucleotide | AT | 26 | 13 |
| DV945102.1 | Contig 180 | dinucleotide | AG | 14 | 7 |
| DV949546.1 | Contig 180 | dinucleotide | AG | 14 | 7 |
| DV956252.1 | Contig 180 | dinucleotide | AG | 16 | 8 |
| DV945161.1 | Contig 181 | dinucleotide | AT | 12 | 6 |
| DV959987.1 | Contig 181 | dinucleotide | AT | 14 | 7 |
| DV945440.1 | Contig 182 | dinucleotide | AT | 14 | 7 |
| DV947291.1 | Contig 182 | dinucleotide | AT | 14 | 7 |
| DV945550.1 | Contig 183 | dinucleotide | AT | 12 | 6 |
| DV958919.1 | Contig 183 | dinucleotide | AT | 12 | 6 |
| DV945831.1 | Contig 184 | trinucleotide | ATC | 27 | 9 |
| DV946993.1 | Contig 184 | trinucleotide | ATC | 18 | 6 |
| DV945932.1 | Contig 185 | dinucleotide | AC | 26 | 13 |
| DV951969.1 | Contig 185 | dinucleotide | AC | 26 | 13 |
| CK234300.1 | Contig 186 | dinucleotide | AT | 16 | 8 |
| CK304724.1 | Contig 186 | dinucleotide | AT | 16 | 8 |
| CK306229.1 | Contig 186 | dinucleotide | AT | 18 | 9 |
| DV945940.1 | Contig 186 | dinucleotide | AT | 14 | 7 |
| DV581760.1 | Contig 187 | trinucleotide | CCG | 18 | 6 |
| DV946330.1 | Contig 187 | trinucleotide | CCG | 18 | 6 |
| DV959091.1 | Contig 187 | trinucleotide | CCG | 18 | 6 |
| CK308969.1 | Contig 188 | trinucleotide | AGG | 21 | 7 |
| CK317042.1 | Contig 188 | trinucleotide | AGG | 18 | 6 |
| DV946483.1 | Contig 188 | trinucleotide | AGG | 27 | 9 |
| DV948294.1 | Contig 188 | trinucleotide | AGG | 21 | 7 |
| DV948657.1 | Contig 188 | trinucleotide | AGG | 27 | 9 |
| DV950621.1 | Contig 188 | trinucleotide | AGG | 24 | 8 |
| CK308425.1 | Contig 189 | trinucleotide | AGG | 24 | 8 |
| DV946615.1 | Contig 189 | trinucleotide | AGG | 24 | 8 |
| DV949956.1 | Contig 189 | trinucleotide | AGG | 18 | 6 |
| DV946757.1 | Contig 190 | trinucleotide | AAC | 21 | 7 |
| DV952153.1 | Contig 190 | trinucleotide | AAC | 21 | 7 |
| DV946860.1 | Contig 191 | trinucleotide | AGC | 18 | 6 |
| DV953832.1 | Contig 191 | trinucleotide | AGC | 18 | 6 |
| DV959257.1 | Contig 191 | trinucleotide | AGC | 18 | 6 |
| CK314748.1 | Contig 192 | trinucleotide | CCG | 24 | 8 |
| DV945197.1 | Contig 192 | trinucleotide | CCG | 27 | 9 |
| DV947051.1 | Contig 192 | trinucleotide | CCG | 27 | 9 |
| DV947250.1 | Contig 192 | trinucleotide | CCG | 18 | 6 |
| DV948614.1 | Contig 192 | trinucleotide | CCG | 27 | 9 |
| DV948812.1 | Contig 192 | trinucleotide | CCG | 30 | 10 |
| DV950436.1 | Contig 192 | trinucleotide | CCG | 24 | 8 |
| DV948092.1 | Contig 193 | trinucleotide | AGC | 30 | 10 |
| DV948326.1 | Contig 193 | trinucleotide | AGC | 30 | 10 |
| DV948103.1 | Contig 194 | dinucleotide | AG | 12 | 6 |
| DV949934.1 | Contig 194 | dinucleotide | AG | 12 | 6 |
| DV948212.1 | Contig 195 | dinucleotide | AT | 12 | 6 |
| DV953240.1 | Contig 195 | dinucleotide | AT | 12 | 6 |
| DV948513.1 | Contig 196 | dinucleotide | AC | 14 | 7 |
| DV957491.1 | Contig 196 | dinucleotide | AC | 14 | 7 |
| CK307067.1 | Contig 197 | dinucleotide | AT | 12 | 6 |
| CK307067.1 | Contig 197 | dinucleotide | AT | 22 | 11 |
| DV948691.1 | Contig 197 | dinucleotide | AT | 12 | 6 |
| DV948691.1 | Contig 197 | dinucleotide | AT | 24 | 12 |
| DV960382.1 | Contig 197 | dinucleotide | AT | 12 | 6 |
| DV960382.1 | Contig 197 | dinucleotide | AT | 22 | 11 |
| CK305955.1 | Contig 198 | dinucleotide | AT | 12 | 6 |
| DV948919.1 | Contig 198 | dinucleotide | AT | 12 | 6 |
| DV950527.1 | Contig 198 | dinucleotide | AT | 12 | 6 |
| DV949123.1 | Contig 199 | trinucleotide | ATC | 24 | 8 |
| DV958442.1 | Contig 199 | trinucleotide | ATC | 24 | 8 |
| DV947661.1 | Contig 200 | trinucleotide | AGG | 21 | 7 |
| DV949152.1 | Contig 200 | trinucleotide | AGG | 21 | 7 |
| DV953865.1 | Contig 200 | trinucleotide | AGG | 21 | 7 |
| DV955271.1 | Contig 200 | trinucleotide | AGG | 21 | 7 |
| CK234352.1 | Contig 201 | trinucleotide | AGG | 18 | 6 |
| CK234490.1 | Contig 201 | trinucleotide | AGG | 18 | 6 |
| CK234778.1 | Contig 201 | trinucleotide | AGG | 18 | 6 |
| CK235050.1 | Contig 201 | trinucleotide | AGG | 18 | 6 |
| CK235480.1 | Contig 201 | trinucleotide | AGG | 18 | 6 |
| CK315276.1 | Contig 201 | trinucleotide | AGG | 18 | 6 |
| DV949171.1 | Contig 201 | trinucleotide | AGG | 18 | 6 |
| DV950369.1 | Contig 201 | trinucleotide | AGG | 18 | 6 |
| DV956795.1 | Contig 201 | trinucleotide | AGG | 21 | 7 |
| DV956889.1 | Contig 201 | trinucleotide | AGG | 18 | 6 |
| DV959086.1 | Contig 201 | trinucleotide | AGG | 18 | 6 |
| DV949288.1 | Contig 202 | dinucleotide | AT | 22 | 11 |
| DV950074.1 | Contig 202 | dinucleotide | AT | 20 | 10 |
| DV949413.1 | Contig 203 | dinucleotide | AG | 16 | 8 |
| DV961082.1 | Contig 203 | dinucleotide | AG | 16 | 8 |
| CK305839.1 | Contig 204 | tetranucleotide | ATCC | 24 | 6 |
| DV573598.1 | Contig 204 | tetranucleotide | ATCC | 24 | 6 |
| DV573599.1 | Contig 204 | tetranucleotide | ATCC | 24 | 6 |
| DV949669.1 | Contig 204 | tetranucleotide | ATCC | 24 | 6 |
| DV960079.1 | Contig 204 | tetranucleotide | ATCC | 24 | 6 |
| DV950014.1 | Contig 205 | dinucleotide | AT | 12 | 6 |
| DV951709.1 | Contig 205 | dinucleotide | AT | 12 | 6 |
| DV950120.1 | Contig 206 | dinucleotide | AT | 16 | 8 |
| DV960140.1 | Contig 206 | dinucleotide | AT | 16 | 8 |
| DV950306.1 | Contig 207 | pentanucleotide | AGCCG | 30 | 6 |
| DV951562.1 | Contig 207 | pentanucleotide | AGCCG | 30 | 6 |
| DV950317.1 | Contig 208 | trinucleotide | AAT | 18 | 6 |
| DV952590.1 | Contig 208 | trinucleotide | AAT | 18 | 6 |
| DV956590.1 | Contig 208 | trinucleotide | AAT | 18 | 6 |
| DV959518.1 | Contig 208 | trinucleotide | AAT | 18 | 6 |
| CK314992.1 | Contig 209 | dinucleotide | AC | 12 | 6 |
| DV946336.1 | Contig 209 | dinucleotide | AC | 12 | 6 |
| DV946336.1 | Contig 209 | dinucleotide | AG | 12 | 6 |
| DV950459.1 | Contig 209 | dinucleotide | AC | 12 | 6 |
| DV950459.1 | Contig 209 | dinucleotide | AG | 12 | 6 |
| DV954534.1 | Contig 209 | dinucleotide | AC | 12 | 6 |
| DV954534.1 | Contig 209 | dinucleotide | AG | 12 | 6 |
| DV956907.1 | Contig 209 | dinucleotide | AC | 12 | 6 |
| DV956907.1 | Contig 209 | dinucleotide | AG | 12 | 6 |
| CK305664.1 | Contig 210 | dinucleotide | AG | 18 | 9 |
| CK305698.1 | Contig 210 | dinucleotide | AG | 16 | 8 |
| DV948984.1 | Contig 210 | dinucleotide | AG | 18 | 9 |
| DV949656.1 | Contig 210 | dinucleotide | AG | 18 | 9 |
| DV950591.1 | Contig 210 | dinucleotide | AG | 18 | 9 |
| DV951030.1 | Contig 210 | dinucleotide | AG | 18 | 9 |
| DV958389.1 | Contig 210 | dinucleotide | AG | 16 | 8 |
| CK235688.1 | Contig 211 | dinucleotide | AG | 12 | 6 |
| CK235688.1 | Contig 211 | dinucleotide | AG | 12 | 6 |
| CK314425.1 | Contig 211 | dinucleotide | AG | 12 | 6 |
| CK314425.1 | Contig 211 | dinucleotide | AT | 12 | 6 |
| CK314425.1 | Contig 211 | dinucleotide | AT | 16 | 8 |
| DV950718.1 | Contig 211 | dinucleotide | AG | 12 | 6 |
| DV953959.1 | Contig 211 | dinucleotide | AG | 12 | 6 |
| DV951334.1 | Contig 212 | dinucleotide | AC | 14 | 7 |
| DV958300.1 | Contig 212 | dinucleotide | AC | 16 | 8 |
| DV951441.1 | Contig 213 | dinucleotide | AT | 16 | 8 |
| DV956860.1 | Contig 213 | dinucleotide | AT | 16 | 8 |
| DV952574.1 | Contig 214 | dinucleotide | AC | 14 | 7 |
| DV955431.1 | Contig 214 | dinucleotide | AC | 14 | 7 |
| DV953354.1 | Contig 215 | trinucleotide | AGC | 18 | 6 |
| DV955836.1 | Contig 215 | trinucleotide | AGC | 21 | 7 |
| DV956098.1 | Contig 215 | trinucleotide | AGC | 21 | 7 |
| DV953675.1 | Contig 216 | dinucleotide | AT | 20 | 10 |
| DV954809.1 | Contig 216 | dinucleotide | AT | 20 | 10 |
| DV955629.1 | Contig 216 | dinucleotide | AT | 20 | 10 |
| DV954197.1 | Contig 217 | dinucleotide | AG | 12 | 6 |
| DV955966.1 | Contig 217 | dinucleotide | AG | 12 | 6 |
| DV954446.1 | Contig 218 | dinucleotide | AC | 22 | 11 |
| DV959507.1 | Contig 218 | dinucleotide | AC | 20 | 10 |
| CK313352.1 | Contig 219 | trinucleotide | AGG | 18 | 6 |
| DV951099.1 | Contig 219 | trinucleotide | AGG | 18 | 6 |
| DV953186.1 | Contig 219 | trinucleotide | AGG | 18 | 6 |
| DV954468.1 | Contig 219 | trinucleotide | AGG | 18 | 6 |
| DV960516.1 | Contig 219 | trinucleotide | AGG | 18 | 6 |
| DV954558.1 | Contig 220 | dinucleotide | AC | 14 | 7 |
| DV957706.1 | Contig 220 | dinucleotide | AC | 22 | 11 |
| DV960729.1 | Contig 220 | dinucleotide | AC | 20 | 10 |
| DV955307.1 | Contig 221 | dinucleotide | AT | 14 | 7 |
| DV955885.1 | Contig 221 | dinucleotide | AT | 14 | 7 |
| CK317138.1 | Contig 222 | dinucleotide | AG | 14 | 7 |
| DV955731.1 | Contig 222 | dinucleotide | AG | 14 | 7 |
| DV956449.1 | Contig 222 | dinucleotide | AG | 14 | 7 |
| DV960930.1 | Contig 222 | dinucleotide | AG | 14 | 7 |
| CK302996.1 | Contig 223 | dinucleotide | AT | 14 | 7 |
| DV946897.1 | Contig 223 | dinucleotide | AT | 14 | 7 |
| DV947218.1 | Contig 223 | dinucleotide | AT | 14 | 7 |
| DV951805.1 | Contig 223 | dinucleotide | AT | 14 | 7 |
| DV955165.1 | Contig 223 | dinucleotide | AT | 14 | 7 |
| DV956226.1 | Contig 223 | dinucleotide | AT | 14 | 7 |
| DV960813.1 | Contig 223 | dinucleotide | AT | 14 | 7 |
| DV950141.1 | Contig 224 | trinucleotide | AAT | 18 | 6 |
| DV950141.1 | Contig 224 | dinucleotide | AG | 20 | 10 |
| DV956156.1 | Contig 224 | trinucleotide | AAT | 18 | 6 |
| DV956156.1 | Contig 224 | dinucleotide | AG | 14 | 7 |
| DV956933.1 | Contig 224 | trinucleotide | AAT | 18 | 6 |
| DV956933.1 | Contig 224 | dinucleotide | AG | 16 | 8 |
| DV957036.1 | Contig 224 | trinucleotide | AAT | 18 | 6 |
| DV957036.1 | Contig 224 | dinucleotide | AG | 20 | 10 |
| DV957767.1 | Contig 224 | trinucleotide | AAT | 18 | 6 |
| DV957767.1 | Contig 224 | dinucleotide | AG | 16 | 8 |
| DV959306.1 | Contig 224 | trinucleotide | AAT | 18 | 6 |
| DV959306.1 | Contig 224 | dinucleotide | AG | 16 | 8 |
| DV959465.1 | Contig 224 | trinucleotide | AAT | 18 | 6 |
| DV959465.1 | Contig 224 | dinucleotide | AG | 16 | 8 |
| DV958073.1 | Contig 225 | dinucleotide | AT | 16 | 8 |
| DV960422.1 | Contig 225 | dinucleotide | AT | 16 | 8 |
| CK302011.1 | Contig 226 | dinucleotide | AG | 34 | 17 |
| DV959049.1 | Contig 226 | dinucleotide | AG | 62 | 31 |
| CK309179.1 | Contig 227 | trinucleotide | AGC | 21 | 7 |
| CK309179.1 | Contig 227 | trinucleotide | AGG | 24 | 8 |
| DV949490.1 | Contig 227 | trinucleotide | AGC | 21 | 7 |
| DV949490.1 | Contig 227 | trinucleotide | AGG | 21 | 7 |
| DV959081.1 | Contig 227 | trinucleotide | AGC | 18 | 6 |
| DV959521.1 | Contig 227 | trinucleotide | AGC | 21 | 7 |
| DV960493.1 | Contig 227 | trinucleotide | AGC | 18 | 6 |
| DV960493.1 | Contig 227 | trinucleotide | AGG | 27 | 9 |
| DV579090.1 | Contig 228 | trinucleotide | AGC | 39 | 13 |
| DV946589.1 | Contig 228 | trinucleotide | AGC | 24 | 8 |
| DV961336.1 | Contig 228 | trinucleotide | AGC | 21 | 7 |
| CK234979.1 | Contig 229 | dinucleotide | AT | 22 | 11 |
| DV948266.1 | Contig 229 | dinucleotide | AT | 22 | 11 |
| DV961773.1 | Contig 229 | dinucleotide | AT | 20 | 10 |
| CK234172.1 |  | dinucleotide | AC | 16 | 8 |
| CK234172.1 |  | dinucleotide | AT | 16 | 8 |
| CK234324.1 |  | trinucleotide | AGG | 21 | 7 |
| CK234814.1 |  | trinucleotide | AGC | 33 | 11 |
| CK234877.1 |  | tetranucleotide | AAAT | 24 | 6 |
| CK234904.1 |  | dinucleotide | AT | 12 | 6 |
| CK234971.1 |  | dinucleotide | AC | 12 | 6 |
| CK235042.1 |  | dinucleotide | AT | 14 | 7 |
| CK235217.1 |  | trinucleotide | AGG | 18 | 6 |
| CK235395.1 |  | trinucleotide | CCG | 21 | 7 |
| CK235504.1 |  | dinucleotide | AT | 12 | 6 |
| CK235723.1 |  | dinucleotide | AG | 12 | 6 |
| CK235805.1 |  | trinucleotide | AAT | 18 | 6 |
| CK235832.1 |  | dinucleotide | AT | 12 | 6 |
| CK301218.1 |  | trinucleotide | AGG | 24 | 8 |
| CK301219.1 |  | pentanucleotide | ACGGC | 45 | 9 |
| CK301253.1 |  | dinucleotide | AT | 12 | 6 |
| CK301254.1 |  | trinucleotide | AAG | 18 | 6 |
| CK301303.1 |  | trinucleotide | CCG | 18 | 6 |
| CK301303.1 |  | dinucleotide | CG | 12 | 6 |
| CK301390.1 |  | dinucleotide | AT | 22 | 11 |
| CK301458.1 |  | tetranucleotide | AGAT | 56 | 14 |
| CK301512.1 |  | dinucleotide | AT | 16 | 8 |
| CK301536.1 |  | pentanucleotide | AAGGG | 30 | 6 |
| CK301552.1 |  | dinucleotide | AT | 14 | 7 |
| CK301583.1 |  | dinucleotide | AT | 12 | 6 |
| CK301759.1 |  | dinucleotide | AC | 14 | 7 |
| CK301777.1 |  | dinucleotide | AT | 12 | 6 |
| CK302040.1 |  | trinucleotide | CCG | 21 | 7 |
| CK302100.1 |  | dinucleotide | AT | 12 | 6 |
| CK302120.1 |  | dinucleotide | AG | 14 | 7 |
| CK302199.1 |  | trinucleotide | CCG | 18 | 6 |
| CK302333.1 |  | trinucleotide | AGC | 21 | 7 |
| CK302478.1 |  | pentanucleotide | AAAAT | 40 | 8 |
| CK302671.1 |  | trinucleotide | AGG | 24 | 8 |
| CK302794.1 |  | dinucleotide | AT | 20 | 10 |
| CK302822.1 |  | dinucleotide | AC | 16 | 8 |
| CK302859.1 |  | trinucleotide | AAT | 51 | 17 |
| CK302873.1 |  | trinucleotide | CCG | 21 | 7 |
| CK302997.1 |  | dinucleotide | AT | 14 | 7 |
| CK302998.1 |  | dinucleotide | AT | 14 | 7 |
| CK303135.1 |  | dinucleotide | AT | 12 | 6 |
| CK303492.1 |  | trinucleotide | AGC | 27 | 9 |
| CK303575.1 |  | trinucleotide | AGC | 33 | 11 |
| CK303607.1 |  | tetranucleotide | AAGG | 24 | 6 |
| CK303727.1 |  | pentanucleotide | AAAAT | 115 | 23 |
| CK303727.1 |  | pentanucleotide | AAATG | 35 | 7 |
| CK303735.1 |  | dinucleotide | AC | 14 | 7 |
| CK303735.1 |  | tetranucleotide | ACAG | 24 | 6 |
| CK304087.1 |  | trinucleotide | ACC | 24 | 8 |
| CK304151.1 |  | dinucleotide | AT | 18 | 9 |
| CK304156.1 |  | dinucleotide | AT | 12 | 6 |
| CK304156.1 |  | dinucleotide | AT | 18 | 9 |
| CK304183.1 |  | dinucleotide | AT | 12 | 6 |
| CK304368.1 |  | dinucleotide | AT | 12 | 6 |
| CK304524.1 |  | pentanucleotide | AATCC | 60 | 12 |
| CK304560.1 |  | dinucleotide | AT | 14 | 7 |
| CK304841.1 |  | dinucleotide | AT | 24 | 12 |
| CK304920.1 |  | trinucleotide | CCG | 21 | 7 |
| CK304956.1 |  | dinucleotide | AT | 16 | 8 |
| CK305005.1 |  | dinucleotide | AT | 26 | 13 |
| CK305147.1 |  | trinucleotide | AAT | 18 | 6 |
| CK305147.1 |  | dinucleotide | AC | 20 | 10 |
| CK305233.1 |  | dinucleotide | AT | 14 | 7 |
| CK305293.1 |  | dinucleotide | AT | 18 | 9 |
| CK305293.1 |  | dinucleotide | AT | 12 | 6 |
| CK305320.1 |  | trinucleotide | AGG | 18 | 6 |
| CK305403.1 |  | dinucleotide | AT | 18 | 9 |
| CK305407.1 |  | trinucleotide | AGG | 18 | 6 |
| CK305719.1 |  | trinucleotide | ACG | 18 | 6 |
| CK305909.1 |  | dinucleotide | AT | 12 | 6 |
| CK306028.1 |  | dinucleotide | AC | 14 | 7 |
| CK306159.1 |  | dinucleotide | AT | 24 | 12 |
| CK306182.1 |  | dinucleotide | AC | 12 | 6 |
| CK306250.1 |  | trinucleotide | AGC | 24 | 8 |
| CK306319.1 |  | dinucleotide | AT | 12 | 6 |
| CK306323.1 |  | trinucleotide | AGG | 21 | 7 |
| CK306359.1 |  | dinucleotide | AC | 14 | 7 |
| CK306467.1 |  | trinucleotide | AGG | 27 | 9 |
| CK306520.1 |  | tetranucleotide | AAAG | 24 | 6 |
| CK306569.1 |  | tetranucleotide | AAAC | 24 | 6 |
| CK306636.1 |  | dinucleotide | AT | 18 | 9 |
| CK306636.1 |  | dinucleotide | AT | 14 | 7 |
| CK306693.1 |  | dinucleotide | AT | 12 | 6 |
| CK306771.1 |  | trinucleotide | CCG | 18 | 6 |
| CK306840.1 |  | trinucleotide | AAC | 18 | 6 |
| CK306870.1 |  | dinucleotide | AG | 12 | 6 |
| CK306873.1 |  | trinucleotide | AAT | 18 | 6 |
| CK306903.1 |  | dinucleotide | AT | 14 | 7 |
| CK306996.1 |  | dinucleotide | AT | 14 | 7 |
| CK307029.1 |  | dinucleotide | AC | 16 | 8 |
| CK307643.1 |  | dinucleotide | AT | 12 | 6 |
| CK307877.1 |  | trinucleotide | AGC | 24 | 8 |
| CK308096.1 |  | dinucleotide | AT | 12 | 6 |
| CK308186.1 |  | trinucleotide | AGG | 18 | 6 |
| CK308599.1 |  | dinucleotide | AT | 14 | 7 |
| CK308620.1 |  | dinucleotide | AT | 12 | 6 |
| CK308661.1 |  | trinucleotide | AAC | 18 | 6 |
| CK308723.1 |  | trinucleotide | AAT | 123 | 41 |
| CK308912.1 |  | tetranucleotide | ACAG | 24 | 6 |
| CK308923.1 |  | trinucleotide | CCG | 27 | 9 |
| CK309076.1 |  | trinucleotide | AGC | 21 | 7 |
| CK309186.1 |  | trinucleotide | ATC | 18 | 6 |
| CK309291.1 |  | trinucleotide | CCG | 21 | 7 |
| CK309340.1 |  | dinucleotide | AT | 12 | 6 |
| CK309486.1 |  | dinucleotide | AC | 14 | 7 |
| CK309535.1 |  | trinucleotide | AAG | 21 | 7 |
| CK309563.1 |  | trinucleotide | CCG | 18 | 6 |
| CK309611.1 |  | dinucleotide | AT | 14 | 7 |
| CK309654.1 |  | dinucleotide | AT | 14 | 7 |
| CK309717.1 |  | trinucleotide | AGC | 18 | 6 |
| CK309775.1 |  | trinucleotide | AAG | 21 | 7 |
| CK309909.1 |  | dinucleotide | AT | 22 | 11 |
| CK310020.1 |  | pentanucleotide | AAAAT | 75 | 15 |
| CK310080.1 |  | dinucleotide | AC | 16 | 8 |
| CK310080.1 |  | dinucleotide | AT | 14 | 7 |
| CK310118.1 |  | trinucleotide | AGC | 18 | 6 |
| CK310425.1 |  | dinucleotide | AT | 12 | 6 |
| CK310425.1 |  | dinucleotide | AT | 12 | 6 |
| CK310740.1 |  | pentanucleotide | ACCGC | 45 | 9 |
| CK310986.1 |  | dinucleotide | AC | 18 | 9 |
| CK311191.1 |  | dinucleotide | AT | 14 | 7 |
| CK311255.1 |  | dinucleotide | AT | 16 | 8 |
| CK311260.1 |  | dinucleotide | AT | 12 | 6 |
| CK311260.1 |  | dinucleotide | AT | 14 | 7 |
| CK311267.1 |  | dinucleotide | AT | 12 | 6 |
| CK311336.1 |  | pentanucleotide | ACGGC | 30 | 6 |
| CK311387.1 |  | dinucleotide | AT | 14 | 7 |
| CK311554.1 |  | tetranucleotide | ACAG | 24 | 6 |
| CK311588.1 |  | dinucleotide | AG | 12 | 6 |
| CK311857.1 |  | dinucleotide | AC | 14 | 7 |
| CK311952.1 |  | dinucleotide | AC | 14 | 7 |
| CK312070.1 |  | dinucleotide | AC | 12 | 6 |
| CK312132.1 |  | trinucleotide | CCG | 21 | 7 |
| CK312252.1 |  | dinucleotide | AC | 12 | 6 |
| CK312294.1 |  | dinucleotide | AC | 16 | 8 |
| CK312317.1 |  | dinucleotide | AT | 12 | 6 |
| CK312347.1 |  | pentanucleotide | AAAGG | 145 | 29 |
| CK312353.1 |  | dinucleotide | AT | 14 | 7 |
| CK312472.1 |  | dinucleotide | AT | 14 | 7 |
| CK312587.1 |  | dinucleotide | AT | 24 | 12 |
| CK312607.1 |  | trinucleotide | AGC | 27 | 9 |
| CK312608.1 |  | dinucleotide | AT | 14 | 7 |
| CK312608.1 |  | dinucleotide | AT | 30 | 15 |
| CK312612.1 |  | dinucleotide | AT | 14 | 7 |
| CK312668.1 |  | dinucleotide | AT | 20 | 10 |
| CK312674.1 |  | trinucleotide | AGG | 18 | 6 |
| CK312725.1 |  | dinucleotide | AT | 14 | 7 |
| CK312948.1 |  | trinucleotide | AGC | 18 | 6 |
| CK312970.1 |  | dinucleotide | AC | 14 | 7 |
| CK312995.1 |  | trinucleotide | CCG | 18 | 6 |
| CK313006.1 |  | dinucleotide | AC | 14 | 7 |
| CK313022.1 |  | dinucleotide | AT | 20 | 10 |
| CK313229.1 |  | trinucleotide | AGG | 18 | 6 |
| CK313415.1 |  | dinucleotide | AC | 18 | 9 |
| CK313435.1 |  | trinucleotide | AGG | 21 | 7 |
| CK313757.1 |  | dinucleotide | AC | 12 | 6 |
| CK313807.1 |  | dinucleotide | AT | 12 | 6 |
| CK313808.1 |  | trinucleotide | AGG | 18 | 6 |
| CK313891.1 |  | trinucleotide | AGC | 24 | 8 |
| CK313919.1 |  | dinucleotide | AT | 14 | 7 |
| CK313967.1 |  | dinucleotide | AG | 12 | 6 |
| CK314074.1 |  | trinucleotide | AGC | 18 | 6 |
| CK314089.1 |  | dinucleotide | AG | 12 | 6 |
| CK314212.1 |  | trinucleotide | ACC | 21 | 7 |
| CK314276.1 |  | dinucleotide | AT | 12 | 6 |
| CK314428.1 |  | dinucleotide | AT | 12 | 6 |
| CK314474.1 |  | dinucleotide | AC | 14 | 7 |
| CK314678.1 |  | dinucleotide | AT | 14 | 7 |
| CK314824.1 |  | trinucleotide | AGC | 21 | 7 |
| CK314884.1 |  | trinucleotide | AGG | 21 | 7 |
| CK314986.1 |  | trinucleotide | AGG | 18 | 6 |
| CK314995.1 |  | trinucleotide | AGG | 18 | 6 |
| CK315053.1 |  | dinucleotide | AG | 20 | 10 |
| CK315131.1 |  | trinucleotide | AGG | 27 | 9 |
| CK315168.1 |  | dinucleotide | AT | 14 | 7 |
| CK315344.1 |  | dinucleotide | AT | 22 | 11 |
| CK315491.1 |  | dinucleotide | AT | 38 | 19 |
| CK315515.1 |  | dinucleotide | AT | 12 | 6 |
| CK315649.1 |  | dinucleotide | AT | 14 | 7 |
| CK315695.1 |  | dinucleotide | AT | 14 | 7 |
| CK315726.1 |  | trinucleotide | CCG | 30 | 10 |
| CK315950.1 |  | trinucleotide | CCG | 18 | 6 |
| CK315984.1 |  | trinucleotide | CCG | 21 | 7 |
| CK316015.1 |  | dinucleotide | AG | 14 | 7 |
| CK316158.1 |  | dinucleotide | AG | 12 | 6 |
| CK316159.1 |  | dinucleotide | AC | 12 | 6 |
| CK316314.1 |  | dinucleotide | AC | 22 | 11 |
| CK316407.1 |  | dinucleotide | AT | 12 | 6 |
| CK316468.1 |  | trinucleotide | AGC | 18 | 6 |
| CK316523.1 |  | dinucleotide | AT | 18 | 9 |
| CK316749.1 |  | dinucleotide | AT | 14 | 7 |
| CK317200.1 |  | pentanucleotide | AAAAC | 35 | 7 |
| CK317333.1 |  | dinucleotide | AT | 20 | 10 |
| CK317471.1 |  | dinucleotide | AC | 14 | 7 |
| CK317536.1 |  | trinucleotide | AAC | 24 | 8 |
| CK317544.1 |  | dinucleotide | AG | 14 | 7 |
| CK317545.1 |  | trinucleotide | AAT | 75 | 25 |
| CK317547.1 |  | dinucleotide | AG | 12 | 6 |
| DV570808.1 |  | trinucleotide | AGG | 30 | 10 |
| DV571585.1 |  | trinucleotide | AGG | 18 | 6 |
| DV571740.1 |  | trinucleotide | CCG | 21 | 7 |
| DV571901.1 |  | dinucleotide | AC | 14 | 7 |
| DV573065.1 |  | trinucleotide | AGG | 18 | 6 |
| DV576383.1 |  | dinucleotide | AC | 12 | 6 |
| DV576713.1 |  | trinucleotide | ATC | 75 | 25 |
| DV578095.1 |  | trinucleotide | AGG | 21 | 7 |
| DV579380.1 |  | dinucleotide | AC | 24 | 12 |
| DV579388.1 |  | trinucleotide | AGG | 21 | 7 |
| DV579436.1 |  | trinucleotide | CCG | 18 | 6 |
| DV579597.1 |  | trinucleotide | AGG | 21 | 7 |
| DV579905.1 |  | trinucleotide | AGC | 36 | 12 |
| DV580387.1 |  | tetranucleotide | ACAG | 24 | 6 |
| DV582822.1 |  | trinucleotide | AGC | 18 | 6 |
| DV944981.1 |  | dinucleotide | AT | 16 | 8 |
| DV944981.1 |  | dinucleotide | AT | 18 | 9 |
| DV944998.1 |  | dinucleotide | AT | 14 | 7 |
| DV945031.1 |  | trinucleotide | AGG | 18 | 6 |
| DV945041.1 |  | dinucleotide | AC | 32 | 16 |
| DV945041.1 |  | dinucleotide | AG | 12 | 6 |
| DV945082.1 |  | trinucleotide | AGG | 21 | 7 |
| DV945345.1 |  | trinucleotide | AGG | 18 | 6 |
| DV945380.1 |  | trinucleotide | AAT | 42 | 14 |
| DV945417.1 |  | trinucleotide | CCG | 24 | 8 |
| DV945503.1 |  | dinucleotide | AT | 18 | 9 |
| DV945581.1 |  | dinucleotide | AC | 14 | 7 |
| DV945624.1 |  | dinucleotide | AT | 14 | 7 |
| DV945670.1 |  | dinucleotide | AT | 24 | 12 |
| DV945695.1 |  | dinucleotide | AT | 12 | 6 |
| DV945699.1 |  | dinucleotide | AT | 14 | 7 |
| DV945713.1 |  | tetranucleotide | AAAT | 24 | 6 |
| DV945733.1 |  | trinucleotide | AGG | 18 | 6 |
| DV945744.1 |  | dinucleotide | AC | 30 | 15 |
| DV945818.1 |  | trinucleotide | AGG | 21 | 7 |
| DV945892.1 |  | dinucleotide | AT | 12 | 6 |
| DV945985.1 |  | pentanucleotide | ACGGC | 75 | 15 |
| DV946067.1 |  | dinucleotide | AC | 16 | 8 |
| DV946206.1 |  | trinucleotide | CCG | 18 | 6 |
| DV946225.1 |  | trinucleotide | CCG | 18 | 6 |
| DV946288.1 |  | dinucleotide | AT | 12 | 6 |
| DV946288.1 |  | dinucleotide | AT | 14 | 7 |
| DV946369.1 |  | pentanucleotide | AGATG | 100 | 20 |
| DV946462.1 |  | trinucleotide | AGG | 24 | 8 |
| DV946590.1 |  | dinucleotide | AC | 14 | 7 |
| DV946644.1 |  | dinucleotide | AG | 18 | 9 |
| DV946651.1 |  | dinucleotide | AT | 16 | 8 |
| DV946652.1 |  | dinucleotide | AC | 20 | 10 |
| DV946731.1 |  | dinucleotide | AC | 14 | 7 |
| DV946811.1 |  | dinucleotide | AT | 14 | 7 |
| DV946835.1 |  | dinucleotide | AG | 20 | 10 |
| DV946840.1 |  | dinucleotide | AC | 16 | 8 |
| DV946946.1 |  | dinucleotide | AC | 12 | 6 |
| DV947101.1 |  | dinucleotide | AT | 12 | 6 |
| DV947267.1 |  | pentanucleotide | AGCCG | 40 | 8 |
| DV947577.1 |  | dinucleotide | AG | 20 | 10 |
| DV947627.1 |  | trinucleotide | AGG | 18 | 6 |
| DV947629.1 |  | trinucleotide | CCG | 21 | 7 |
| DV947640.1 |  | dinucleotide | AT | 12 | 6 |
| DV947660.1 |  | trinucleotide | AGG | 27 | 9 |
| DV947669.1 |  | dinucleotide | AT | 20 | 10 |
| DV947723.1 |  | dinucleotide | AT | 12 | 6 |
| DV947923.1 |  | trinucleotide | CCG | 18 | 6 |
| DV947938.1 |  | trinucleotide | AGG | 21 | 7 |
| DV948031.1 |  | dinucleotide | AC | 12 | 6 |
| DV948064.1 |  | trinucleotide | AAG | 18 | 6 |
| DV948146.1 |  | dinucleotide | AC | 14 | 7 |
| DV948204.1 |  | dinucleotide | AG | 12 | 6 |
| DV948210.1 |  | dinucleotide | AC | 12 | 6 |
| DV948210.1 |  | dinucleotide | AT | 20 | 10 |
| DV948303.1 |  | dinucleotide | AC | 28 | 14 |
| DV948303.1 |  | dinucleotide | AC | 14 | 7 |
| DV948378.1 |  | trinucleotide | AAT | 18 | 6 |
| DV948378.1 |  | trinucleotide | AAT | 21 | 7 |
| DV948467.1 |  | dinucleotide | AT | 14 | 7 |
| DV948584.1 |  | trinucleotide | CCG | 21 | 7 |
| DV948632.1 |  | trinucleotide | CCG | 21 | 7 |
| DV948799.1 |  | trinucleotide | CCG | 18 | 6 |
| DV948825.1 |  | dinucleotide | AG | 12 | 6 |
| DV948966.1 |  | dinucleotide | AG | 12 | 6 |
| DV948966.1 |  | dinucleotide | AG | 14 | 7 |
| DV949023.1 |  | trinucleotide | AGG | 21 | 7 |
| DV949035.1 |  | dinucleotide | AT | 14 | 7 |
| DV949035.1 |  | dinucleotide | AT | 16 | 8 |
| DV949035.1 |  | dinucleotide | AT | 12 | 6 |
| DV949035.1 |  | dinucleotide | AT | 12 | 6 |
| DV949044.1 |  | dinucleotide | AT | 16 | 8 |
| DV949158.1 |  | trinucleotide | AAT | 27 | 9 |
| DV949246.1 |  | trinucleotide | CCG | 27 | 9 |
| DV949408.1 |  | trinucleotide | AAT | 18 | 6 |
| DV949447.1 |  | dinucleotide | AT | 20 | 10 |
| DV949483.1 |  | dinucleotide | AC | 14 | 7 |
| DV949483.1 |  | dinucleotide | AT | 12 | 6 |
| DV949822.1 |  | trinucleotide | AGC | 21 | 7 |
| DV949861.1 |  | dinucleotide | AC | 16 | 8 |
| DV949974.1 |  | dinucleotide | AT | 14 | 7 |
| DV950042.1 |  | dinucleotide | AC | 14 | 7 |
| DV950046.1 |  | dinucleotide | AC | 14 | 7 |
| DV950070.1 |  | dinucleotide | AC | 18 | 9 |
| DV950134.1 |  | dinucleotide | AC | 16 | 8 |
| DV950155.1 |  | pentanucleotide | AAACC | 35 | 7 |
| DV950249.1 |  | trinucleotide | AGG | 27 | 9 |
| DV950285.1 |  | dinucleotide | AT | 14 | 7 |
| DV950329.1 |  | dinucleotide | AC | 14 | 7 |
| DV950391.1 |  | dinucleotide | AT | 18 | 9 |
| DV950668.1 |  | dinucleotide | AC | 14 | 7 |
| DV950733.1 |  | tetranucleotide | AAAT | 24 | 6 |
| DV950873.1 |  | dinucleotide | AT | 12 | 6 |
| DV950884.1 |  | trinucleotide | AGC | 24 | 8 |
| DV951489.1 |  | dinucleotide | AC | 16 | 8 |
| DV951508.1 |  | trinucleotide | CCG | 18 | 6 |
| DV951550.1 |  | tetranucleotide | ATCC | 36 | 9 |
| DV951593.1 |  | trinucleotide | CCG | 18 | 6 |
| DV951730.1 |  | dinucleotide | AC | 14 | 7 |
| DV951916.1 |  | dinucleotide | AC | 20 | 10 |
| DV952012.1 |  | dinucleotide | AG | 12 | 6 |
| DV952025.1 |  | trinucleotide | AAT | 21 | 7 |
| DV952112.1 |  | trinucleotide | AGC | 18 | 6 |
| DV952150.1 |  | dinucleotide | AC | 14 | 7 |
| DV952298.1 |  | dinucleotide | AC | 16 | 8 |
| DV952331.1 |  | dinucleotide | AG | 14 | 7 |
| DV952431.1 |  | dinucleotide | AT | 12 | 6 |
| DV952490.1 |  | trinucleotide | AAT | 30 | 10 |
| DV952512.1 |  | dinucleotide | AG | 12 | 6 |
| DV952581.1 |  | dinucleotide | AG | 22 | 11 |
| DV952619.1 |  | trinucleotide | AGG | 24 | 8 |
| DV952645.1 |  | trinucleotide | AAG | 18 | 6 |
| DV952757.1 |  | dinucleotide | AC | 14 | 7 |
| DV952886.1 |  | pentanucleotide | AAAAG | 45 | 9 |
| DV952886.1 |  | pentanucleotide | AAAAG | 30 | 6 |
| DV952960.1 |  | dinucleotide | AG | 14 | 7 |
| DV953002.1 |  | trinucleotide | AGC | 18 | 6 |
| DV953081.1 |  | dinucleotide | AT | 16 | 8 |
| DV953125.1 |  | dinucleotide | AT | 16 | 8 |
| DV953516.1 |  | dinucleotide | AC | 14 | 7 |
| DV953560.1 |  | dinucleotide | AT | 18 | 9 |
| DV953823.1 |  | trinucleotide | CCG | 18 | 6 |
| DV953824.1 |  | trinucleotide | AGC | 18 | 6 |
| DV953928.1 |  | trinucleotide | AGC | 21 | 7 |
| DV954033.1 |  | trinucleotide | AGG | 30 | 10 |
| DV954070.1 |  | dinucleotide | AG | 16 | 8 |
| DV954174.1 |  | trinucleotide | CCG | 18 | 6 |
| DV954184.1 |  | dinucleotide | AC | 14 | 7 |
| DV954184.1 |  | dinucleotide | AT | 14 | 7 |
| DV954238.1 |  | trinucleotide | AGG | 27 | 9 |
| DV954286.1 |  | dinucleotide | AG | 14 | 7 |
| DV954320.1 |  | tetranucleotide | AGAT | 60 | 15 |
| DV954349.1 |  | trinucleotide | CCG | 21 | 7 |
| DV954445.1 |  | trinucleotide | AGG | 24 | 8 |
| DV954489.1 |  | trinucleotide | AGG | 21 | 7 |
| DV954533.1 |  | dinucleotide | AG | 14 | 7 |
| DV954563.1 |  | trinucleotide | AGC | 18 | 6 |
| DV954568.1 |  | trinucleotide | AGG | 21 | 7 |
| DV954659.1 |  | dinucleotide | AT | 12 | 6 |
| DV954855.1 |  | dinucleotide | AC | 12 | 6 |
| DV954971.1 |  | dinucleotide | AT | 16 | 8 |
| DV954989.1 |  | trinucleotide | CCG | 18 | 6 |
| DV955012.1 |  | dinucleotide | AC | 22 | 11 |
| DV955036.1 |  | dinucleotide | AG | 14 | 7 |
| DV955050.1 |  | dinucleotide | AT | 12 | 6 |
| DV955061.1 |  | dinucleotide | AT | 18 | 9 |
| DV955253.1 |  | dinucleotide | AG | 22 | 11 |
| DV955666.1 |  | dinucleotide | AC | 12 | 6 |
| DV956003.1 |  | trinucleotide | AAT | 18 | 6 |
| DV956013.1 |  | trinucleotide | AAG | 21 | 7 |
| DV956088.1 |  | dinucleotide | AC | 18 | 9 |
| DV956088.1 |  | dinucleotide | AC | 16 | 8 |
| DV956106.1 |  | tetranucleotide | AAAC | 28 | 7 |
| DV956129.1 |  | dinucleotide | AC | 16 | 8 |
| DV956171.1 |  | trinucleotide | CCG | 18 | 6 |
| DV956212.1 |  | dinucleotide | AT | 22 | 11 |
| DV956331.1 |  | pentanucleotide | ACGGC | 50 | 10 |
| DV956346.1 |  | trinucleotide | CCG | 21 | 7 |
| DV956359.1 |  | dinucleotide | AT | 12 | 6 |
| DV956391.1 |  | dinucleotide | AC | 36 | 18 |
| DV956461.1 |  | dinucleotide | CG | 14 | 7 |
| DV956499.1 |  | trinucleotide | AGC | 21 | 7 |
| DV956516.1 |  | trinucleotide | AGG | 27 | 9 |
| DV956755.1 |  | trinucleotide | AGC | 18 | 6 |
| DV956814.1 |  | trinucleotide | AGC | 18 | 6 |
| DV956834.1 |  | dinucleotide | AC | 12 | 6 |
| DV956958.1 |  | dinucleotide | AG | 12 | 6 |
| DV957359.1 |  | dinucleotide | AT | 18 | 9 |
| DV957402.1 |  | trinucleotide | AGC | 18 | 6 |
| DV957432.1 |  | dinucleotide | CG | 12 | 6 |
| DV957464.1 |  | dinucleotide | AT | 14 | 7 |
| DV957488.1 |  | trinucleotide | CCG | 18 | 6 |
| DV957622.1 |  | trinucleotide | CCG | 21 | 7 |
| DV957671.1 |  | dinucleotide | AT | 12 | 6 |
| DV957674.1 |  | dinucleotide | AG | 18 | 9 |
| DV957674.1 |  | dinucleotide | AG | 20 | 10 |
| DV957774.1 |  | dinucleotide | AT | 14 | 7 |
| DV958074.1 |  | dinucleotide | AG | 14 | 7 |
| DV958100.1 |  | dinucleotide | AC | 12 | 6 |
| DV958114.1 |  | dinucleotide | AT | 12 | 6 |
| DV958291.1 |  | dinucleotide | AT | 12 | 6 |
| DV958353.1 |  | dinucleotide | AC | 14 | 7 |
| DV958359.1 |  | dinucleotide | AC | 18 | 9 |
| DV958521.1 |  | trinucleotide | AGG | 24 | 8 |
| DV958593.1 |  | dinucleotide | AT | 32 | 16 |
| DV958695.1 |  | trinucleotide | AAT | 18 | 6 |
| DV958821.1 |  | dinucleotide | AG | 12 | 6 |
| DV958877.1 |  | dinucleotide | AC | 22 | 11 |
| DV958940.1 |  | dinucleotide | AC | 20 | 10 |
| DV959146.1 |  | dinucleotide | AT | 16 | 8 |
| DV959171.1 |  | dinucleotide | AT | 14 | 7 |
| DV959185.1 |  | tetranucleotide | ATCC | 24 | 6 |
| DV959246.1 |  | dinucleotide | AT | 20 | 10 |
| DV959397.1 |  | dinucleotide | AC | 16 | 8 |
| DV959460.1 |  | dinucleotide | AT | 12 | 6 |
| DV959480.1 |  | trinucleotide | AGG | 30 | 10 |
| DV959535.1 |  | trinucleotide | AGG | 21 | 7 |
| DV959539.1 |  | dinucleotide | AT | 16 | 8 |
| DV959600.1 |  | dinucleotide | AT | 12 | 6 |
| DV959679.1 |  | dinucleotide | AT | 12 | 6 |
| DV959812.1 |  | dinucleotide | AC | 16 | 8 |
| DV959820.1 |  | dinucleotide | AT | 14 | 7 |
| DV959937.1 |  | dinucleotide | AC | 12 | 6 |
| DV959997.1 |  | dinucleotide | AC | 18 | 9 |
| DV959997.1 |  | dinucleotide | AT | 12 | 6 |
| DV960076.1 |  | dinucleotide | AT | 16 | 8 |
| DV960130.1 |  | trinucleotide | AGG | 18 | 6 |
| DV960131.1 |  | dinucleotide | AT | 14 | 7 |
| DV960138.1 |  | trinucleotide | AAT | 21 | 7 |
| DV960138.1 |  | trinucleotide | AAT | 21 | 7 |
| DV960224.1 |  | dinucleotide | AT | 16 | 8 |
| DV960227.1 |  | trinucleotide | AGG | 18 | 6 |
| DV960231.1 |  | dinucleotide | AT | 12 | 6 |
| DV960252.1 |  | tetranucleotide | ACGG | 24 | 6 |
| DV960503.1 |  | dinucleotide | AT | 14 | 7 |
| DV960704.1 |  | dinucleotide | AT | 24 | 12 |
| DV960964.1 |  | dinucleotide | AT | 12 | 6 |
| DV961016.1 |  | trinucleotide | AGC | 51 | 17 |
| DV961039.1 |  | dinucleotide | AC | 12 | 6 |
| DV961064.1 |  | trinucleotide | AAT | 21 | 7 |
| DV961081.1 |  | dinucleotide | AC | 12 | 6 |
| DV961169.1 |  | dinucleotide | AG | 12 | 6 |
| DV961369.1 |  | dinucleotide | AT | 16 | 8 |
| DV961910.1 |  | trinucleotide | ACC | 21 | 7 |
| DV961990.1 |  | dinucleotide | AT | 12 | 6 |
