# Supplementary material for: Simple sequence repeats in zebra finch (Taeniopygia guttata) expressed sequence tags: a new resource for evolutionary genetic studies of passerines
Source: BMC Genomics. 2007 Feb 14;8:52. doi: 10.1186/1471-2164-8-52 (PMC1804275; doi:10.1186/1471-2164-8-52)
Supplement: Additional File 3 — Predicted location of orthologs of zebra finch EST-SSRs in the chicken genome. Map indicating location of orthologs on zebra finch EST-SSRs on Assembly 2.1 of the chicken genome. [file 1471-2164-8-52-S3.pdf]

Additional File 3: Predicted location of orthologs of zebra finch EST-SSRs in the chicken genome.

SSRs of different repeat unit length are colour coded: black = dinucleotide, red = trinucleotide, green = tetranucleotide, purple = pentanucleotide. Uninterrupted SSRs are underlined. Positions are in million base pairs (Mbp).

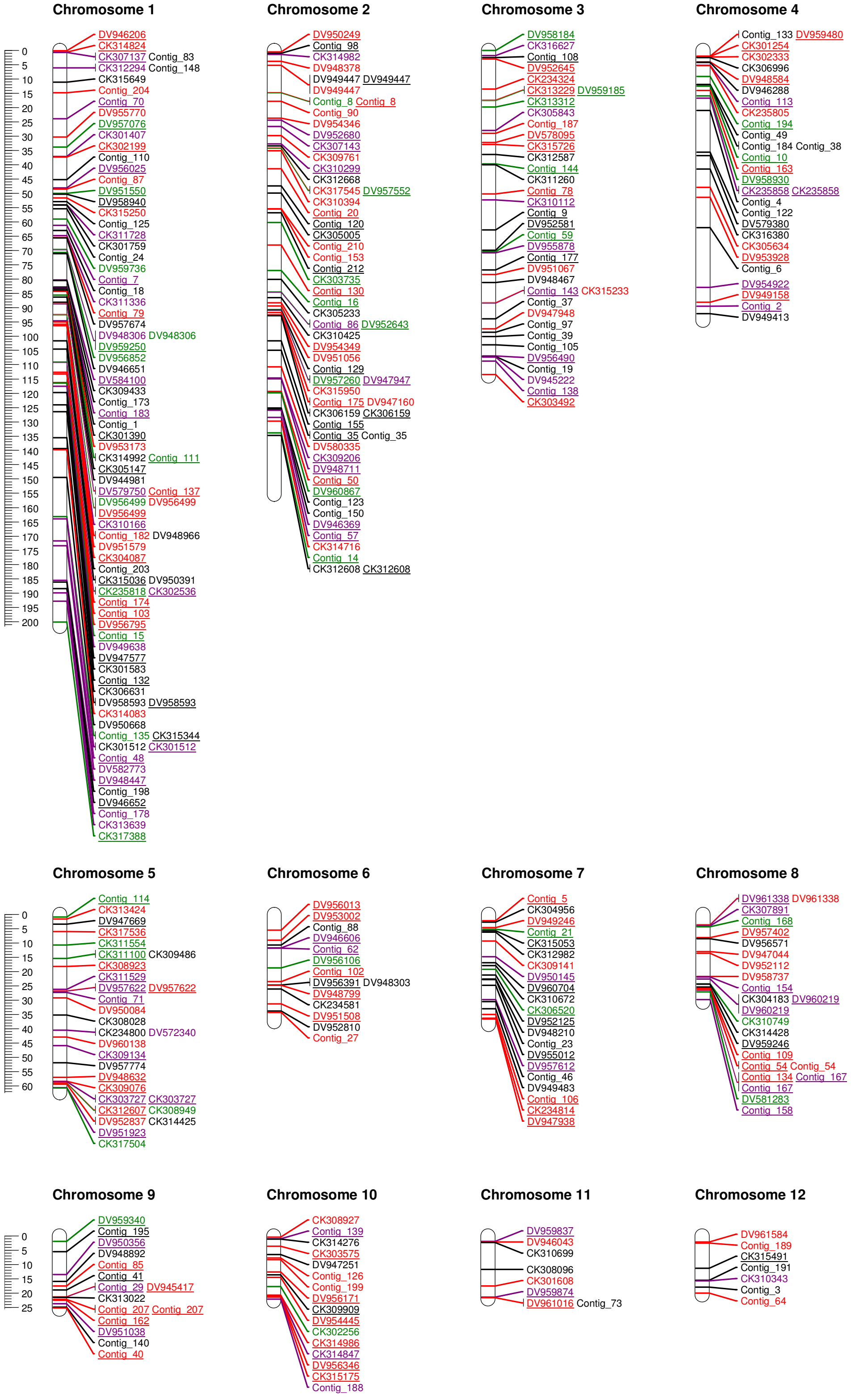

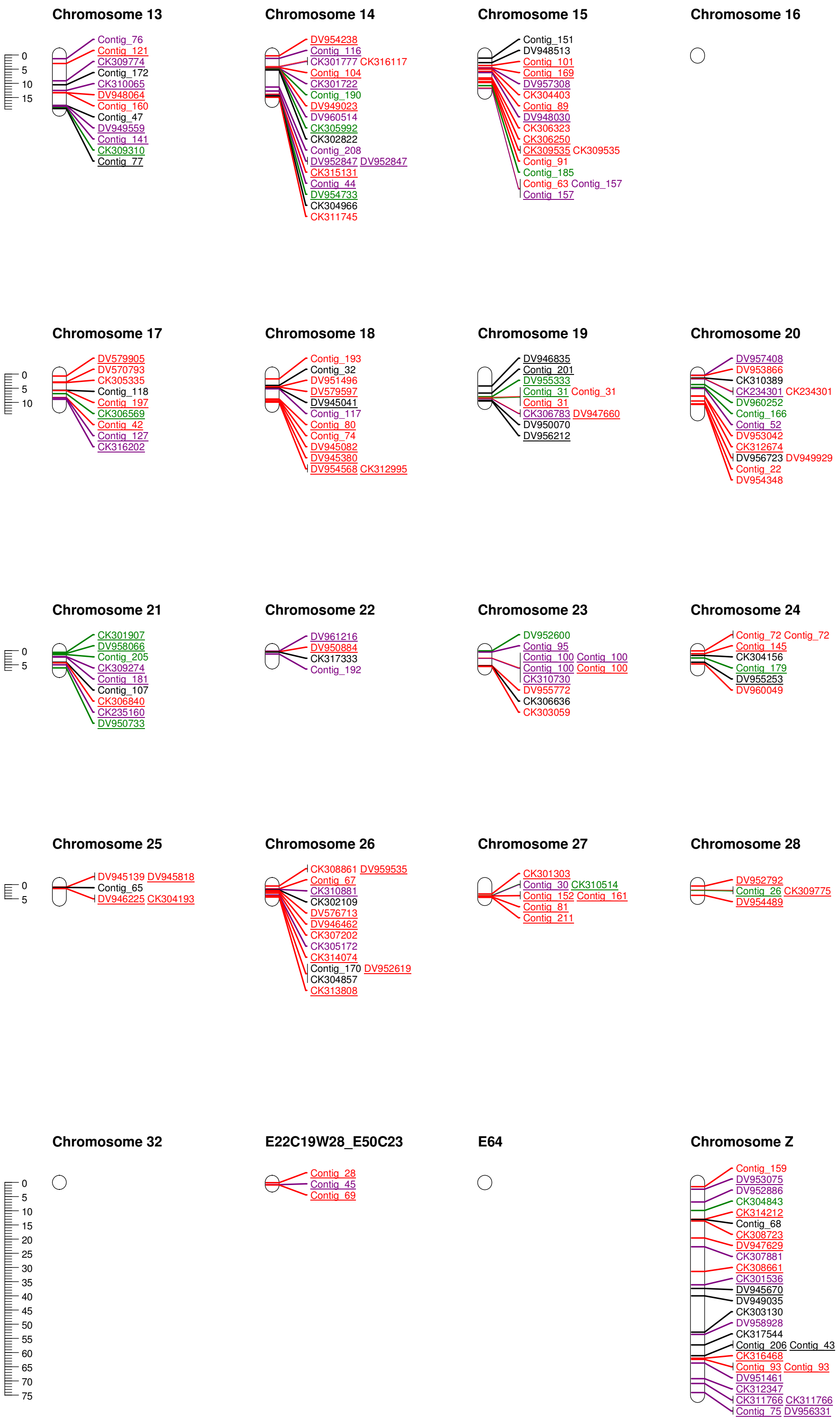

Chromosome 13
Contig_76
Contig_121
CK309774
Contig_172
CK310065
DV948064
Contig_160
Contig_47
DV949559
Contig_141
CK309310
Contig_77
0
5
10
15
Chromosome 14
DV954238
Contig_116
CK301777 CK316117
Contig_104
CK301722
Contig_190
DV949023
DV960514
CK305992
CK302822
Contig_208
DV952847 DV952847
CK315131
Contig_44
DV954733
CK304966
CK311745
Chromosome 15
Contig_151
DV948513
Contig_101
Contig_169
DV957308
CK304403
Contig_89
DV948030
CK306323
CK306250
CK309535 CK309535
Contig_91
Contig_185
Contig_63 Contig_157
Contig_157
Chromosome 16
Chromosome 17
DV579905
DV570793
CK305335
Contig_118
Contig_197
CK306569
Contig_42
Contig_127
CK316202
0
5
10
Chromosome 18
Contig_193
Contig_32
DV951496
DV579597
DV945041
Contig_117
Contig_80
Contig_74
DV945082
DV945380
DV954568 CK312995
Chromosome 19
DV946835
Contig_201
DV955333
Contig_31 Contig_31
Contig_31
CK306783 DV947660
DV950070
DV956212
Chromosome 20
DV957408
DV953866
CK310389
CK234301 CK234301
DV960252
Contig_166
Contig_52
DV953042
CK312674
DV956723 DV949929
Contig_22
DV954348
Chromosome 21
CK301907
DV958066
Contig_205
CK309274
Contig_181
Contig_107
CK306840
CK235160
DV950733
0
5
Chromosome 22
DV961216
DV950884
CK317333
Contig_192
Chromosome 23
DV952600
Contig_95
Contig_100 Contig_100
Contig_100 Contig_100
CK310730
DV955772
CK306636
CK303059
Chromosome 24
Contig_72 Contig_72
Contig_145
CK304156
Contig_179
DV955253
DV960049
Chromosome 25
DV945139 DV945818
Contig_65
DV946225 CK304193
0
5
Chromosome 26
CK308861 DV959535
Contig_67
CK310881
CK302109
DV576713
DV946462
CK307202
CK305172
CK314074
Contig_170 DV952619
CK304857
CK313808
Chromosome 27
CK301303
Contig_30 CK310514
Contig_152 Contig_161
Contig_81
Contig_211
Chromosome 28
DV952792
Contig_26 CK309775
DV954489
Chromosome 32
0
5
10
15
20
25
30
35
40
45
50
55
60
65
70
75
E22C19W28_E50C23
Contig_28
Contig_45
Contig_69
E64
Chromosome Z
Contig_159
DV953075
DV952886
CK304843
CK314212
Contig_68
CK308723
DV947629
CK307881
CK308661
CK301536
DV945670
DV949035
CK303130
DV958928
CK317544
Contig_206 Contig_43
CK316468
Contig_93 Contig_93
DV951461
CK312347
CK311766 CK311766
Contig_75 DV956331
